# Bacterial pathogen deploys iminosugar galactosyrin to manipulate plant glycobiology

**DOI:** 10.1101/2025.02.13.638044

**Published:** 2025-02-14

**Authors:** Nattapong Sanguankiattichai, Balakumaran Chandrasekar, Yuewen Sheng, Nathan Hardenbrook, Werner W. A. Tabak, Daniel Krahn, Margit Drapal, Pierre Buscaill, Suzuka Yamamoto, Atsushi Kato, Robert Nash, George Fleet, Paul Fraser, Markus Kaiser, Peijun Zhang, Gail M. Preston, Renier A. L. van der Hoorn

**Affiliations:** 1 Department of Biology, University of Oxford; Oxford, United Kingdom.; 2 Diamond Light Source, Harwell Science and Innovation Campus; Didcot, United Kingdom.; 3 Division of Structural Biology, Wellcome Trust Centre for Human Genetics, University of Oxford; Oxford, United Kingdom.; 4 ZMB Chemical Biology, Faculty of Biology, University of Duisburg-Essen; Essen, Germany.; 5 Leibniz Institut für analytische Wissenschaften ISAS e.V.; Dortmund, Germany.; 6 Department of Biological Sciences, Royal Holloway University of London; Egham, United Kingdom.; 7 Department of Hospital Pharmacy, University of Toyama; Toyama, Japan.; 8 Institute of Biological, Environmental and Rural Sciences/Phytoquest Limited; Aberystwyth, United Kingdom.; 9 Chemistry Research Laboratory, Department of Chemistry, University of Oxford; Oxford, United Kingdom.

## Abstract

The extracellular space (apoplast) of plants is an important molecular battleground during infection by many pathogens. We previously found that a plant-secreted β-galactosidase BGAL1 acts in immunity by facilitating the release of immunogenic peptides from bacterial flagellin and that *Pseudomonas syringae* suppresses this enzyme by producing a small molecule inhibitor called galactosyrin. Here, we elucidated the structure and biosynthesis of galactosyrin and uncovered its multifunctional roles during infection. Structural elucidation by cryo-EM and chemical synthesis revealed that galactosyrin is an iminosugar featuring a unique geminal diol attached to the pyrrolidine moiety that mimics galactose binding to the β-galactosidase active site. Galactosyrin biosynthesis branches off from purine biosynthesis and involves three enzymes of which the first is a reductase that is unique in iminosugar biosynthesis. Besides inhibiting BGAL1 to avoid detection, galactosyrin also changes the glycoproteome and metabolome of the apoplast. The manipulation of host glycobiology may be common to plant-associated bacteria that carry putative iminosugar biosynthesis clusters.

## INTRODUCTION

The extracellular space in plant tissues (the apoplast) is an important molecular battleground during plant-pathogen interactions (1). This microenvironment is colonized by bacteria, fungi and oomycetes that must have evolved various strategies to avoid recognition, suppress immune responses and manipulate host physiology. Yet most of these apoplastic plant-pathogen interactions remain to be elucidated. Our previous work on the interaction between *Nicotiana benthamiana* plants and the model bacterial pathogen *Pseudomonas syringae* revealed the role of plant apoplastic β-galactosidase BGAL1 in plant immunity (2). BGAL1 initiates the hydrolytic release of immunogenic peptides from glycosylated flagella of *P. syringae* that activate plant defences (2). Interestingly, we also found that during infection, *P. syringae* pv. *tomato* DC3000 produces a small molecule inhibitor of BGAL1, which we named galactosyrin (2). In this work, we report the molecular structure of galactosyrin and its full biosynthesis pathway. This molecule represents a novel iminosugar class and has multifunctional roles in manipulating the extracellular glycoproteome and metabolome during infection.

## RESULTS

### Galactosyrin biosynthesis gene cluster expression is controlled by virulence gene regulators

**Fig. 1.**
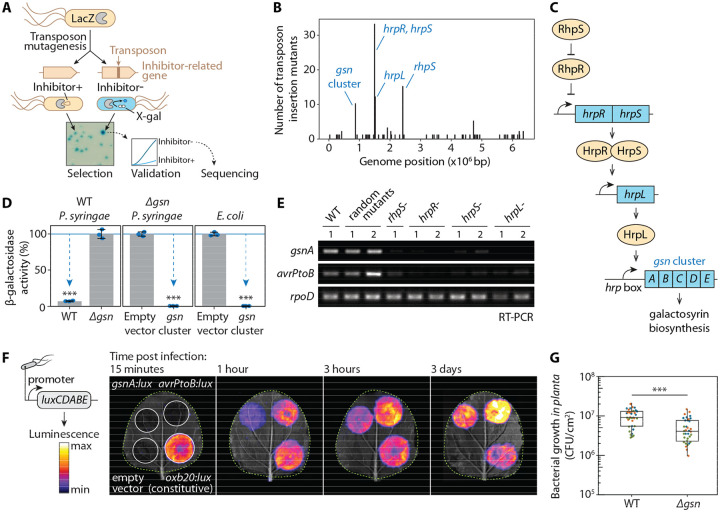
Galactosyrin biosynthesis gene cluster and its regulators identified by forward genetics. **(A)** Genetic screen for galactosyrin-deficient mutants. *P. syringae* expressing LacZ β-galactosidase was used to create a random transposon insertion mutant library. When plated onto a virulence-inducing medium supplemented with X-gal, galactosyrin-deficient mutants cannot inhibit LacZ, resulting in a darker blue colour. These candidate mutants were validated in an enzymatic assay for the inability to produce galactosyrin. Confirmed mutants were sequenced to identify transposon insertion sites. **(B)** Histogram with number of transposon insertion sites identified from galactosyrin mutants along the position within the genome, showing four hotspots corresponding to the *gsn* gene cluster and virulence regulators *hrpR*, *hrpS*, *hrpL* and *rhpS*. **(C)** Summary of the roles of genes required for galactosyrin production. The *gsn* cluster (containing five genes *gsnABCDE*, PSPTO0834-8) confers galactosyrin biosynthesis. The expression of the *gsn* cluster is controlled by a regulatory cascade of type III secretion system regulators (RhpS, HrpR, HrpS and HrpL), which controls virulence gene induction during infection. The promotor of the *gsn* cluster contains the binding site of the HrpL transcriptional activator (*hrp* box). **(D)** The *gsn* cluster confers galactosyrin biosynthesis in *P. syringae* and *E. coli*. Bacterial strains were grown in virulence-inducing medium and the supernatant was tested for LacZ inhibition using purified LacZ and substrate FDG (Fluorescein di(-β-D-Galactopyranoside). β-galactosidase activity is reported as a percentage of the activity relative to the mean of the no-inhibitor-control (*Δgsn* or empty vector). Arrows highlight significant inhibition. Error bars represent standard deviation from 3 replicates. Asterisks indicate statistically significant difference compared to no-inhibitor-control (P < 0.001) using Welch’s t-test. **(E)** Expression of the *gsn* cluster is dependent on *hrpR*, *hrpS*, *hrpL* and *rhpS*. Bacterial strains were grown in virulence-inducing medium, then total RNA was extracted for reverse transcription polymerase chain reaction (RT-PCR) to monitor transcript levels of *gsnA, avrPtoB* (type III secreted effector gene) and *rpoD* (reference gene). **(F)** The *gsn* cluster is transcribed during infection. Bacteria carrying various promoter:*luxCDABE* reporter fusion constructs were infiltrated into *N. benthamiana* leaves and luminescence was imaged at different time points after infection. Signals displayed are scaled to the maximum and minimum within each image. Leaves are outlined with dashed lines. **(G)** *gsn* cluster contributes to virulence. Bacterial strains were spray-inoculated on *N. benthamiana* leaves then bacterial growth (number of bacterial colony forming units (CFU) per cm^2^ of leaf) was quantified at 3 days post infection. Results from 3 independent experiments with 12 replicates each are plotted in different colours. Asterisks indicate statistically significant difference between strains (P < 0.001) using two-way ANOVA with experiments as blocks.

To identify genes required for galactosyrin biosynthesis, we transformed *P. syringae* pv. *tomato* DC3000 Δ*hopQ1–1* (3) (called wild-type for galactosyrin (WT) in this work) with *lacZ* encoding the β-galactosidase from *Escherichia coli*, which is routinely used for blue staining with X-gal (5-bromo-4-chloro-3-indoyl-β-D-galactopyranoside). We then performed Tn5-transposon mutagenesis and selection on virulence-inducing medium containing X-gal to identify darker blue colonies of mutants that cannot produce galactosyrin to inhibit LacZ (Fig. 1A). The loss of galactosyrin was confirmed in activity assays with purified LacZ and a fluorogenic substrate (Fig. S1) and transposon insertion sites were identified for 140 galactosyrin-deficient mutants (Table S1). These Tn5 insertion sites concentrated in four virulence gene regulators (*hrpR, hrpS, hrpL* and *rhpS*) and one putative galactosyrin biosynthesis gene cluster (*gsn*, locus tags PSPTO_0834–8, new locus tags PSPTO_RS04425-RS04445, Fig. 1B).

The *gsn* cluster contains five genes encoding three biosynthesis enzymes (GsnA/B/C), a protein of unknown function (GsnD), and a transporter (GsnE) (Fig. 1C). The deletion mutant lacking the *gsn* cluster (*Δgsn*) is unable to produce the inhibitor, and transformation of this mutant with a plasmid carrying the *gsn* cluster restores inhibitor production (Fig. 1D). Galactosyrin production was also established in *E. coli* upon transformation with the plasmid carrying the *gsn* cluster (Fig. 1D). These results confirm that the *gsn* gene cluster is necessary and sufficient for galactosyrin production in bacteria.

The promoter of the *gsn* gene cluster contains the *hrp* box, a conserved binding site for transcription factor HrpL (4), which is transcriptionally regulated by HrpR/S and RhpS (5) (Fig. 1C), RhpS, HrpR/S and HrpL are master regulators of virulence genes including type-III effectors such as *avrPtoB* (5). Indeed, expression of the *gsn* cluster is impaired in *rhpS, hrpR/S* and *hrpL* mutants, like *avrPtoB* (Fig. 1E), clarifying why these mutants are galactosyrin deficient. Consequently, as demonstrated with a *gsn:lux* reporter strain, the *gsn* cluster is transcribed from the initial to late stages of infection (Fig. 1F), consistent with inhibitor production during infection (2). When compared to WT bacteria, the *Δgsn* mutant has reduced growth in *N. benthamiana* (Fig. 1G) but not *in vitro* (Fig. S2), indicating that the *gsn* cluster produces a virulence factor during infection. This is also consistent with reduced virulence described earlier for a *gsnA* mutant in *Arabidopsis thaliana* (4).

The *gsn* cluster is present in various strains across the major phylogroups of *P. syringae* (Fig. S3A), but the phylogeny of *gsnA* is incongruent with that of *P. syringae* (Fig. S3B). The *gsn* cluster is also flanked by transposable elements located downstream of tRNA^Lys^ loci (Fig. S3A, C), which are typical for integrase sites (6). Together with the fact that the *gsn* cluster has lower GC content than its neighboring regions and the genomic average (Fig. S3D), these data indicate that the *gsn* cluster has been distributed in *P. syringae* through horizontal gene transfer. Furthermore, GsnA homologs (aldehyde dehydrogenases, ADHs) are present in diverse bacterial species in different gene clusters with similar gene functions (Fig. S4), some of which are known to produce distinct iminosugars, potent glycosidase inhibitors with sugar-like structures containing a nitrogen instead of oxygen in the ring (Fig. S4) (7–9). However, unlike previously characterised gene clusters, the *gsn* cluster forms a distinct clade that also encodes GsnB (homolog of reductase RibD) (Fig. S4), suggesting that galactosyrin could be a novel iminosugar produced by a yet unknown metabolic pathway.

### Galactosyrin structure and inhibition mechanism resolved by cryo-EM

**Fig. 2.**
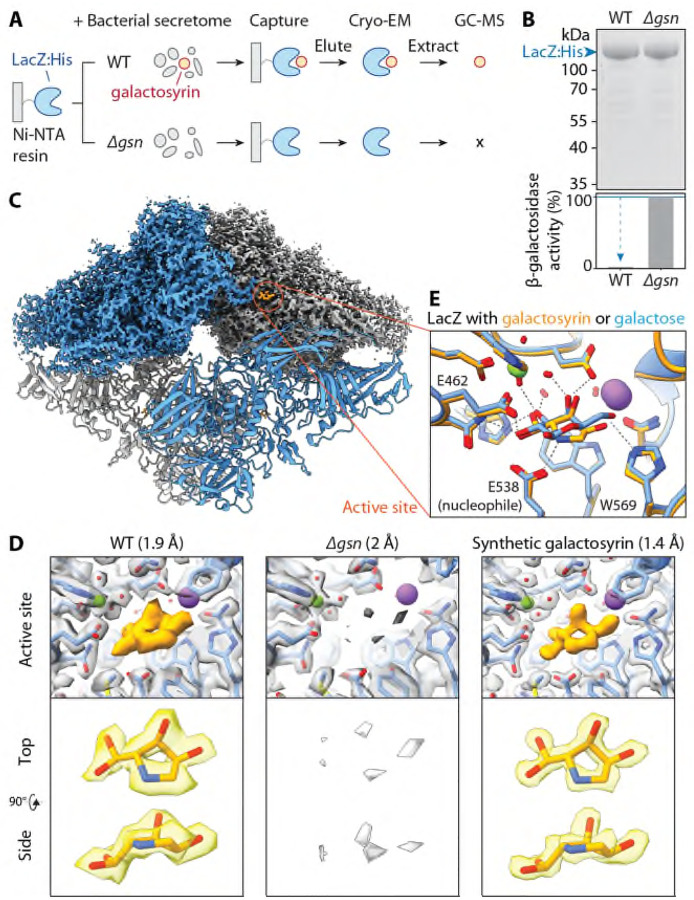
Galactosyrin is a hydrated pyrrolidine of a novel iminosugar class. **(A)** LacZ-galactosyrin complex capture and downstream analyses. A Histidine-tagged β-galactosidase enzyme from *E. coli* (LacZ:His) immobilised on Ni-NTA beads was used to capture galactosyrin inhibitor from crude bacterial secretome of galactosyrin-producing *P. syringae* (WT) or the galactosyrin-deficient mutant (*Δgsn*, negative control). After washing, the complex was eluted and used for cryo-electron microscopy (Cryo-EM), and soluble metabolites were extracted for analysis by gas chromatography-mass spectrometry (GC-MS). **(B)** Captured LacZ is saturated with galactosyrin. (Top) Total protein stain of eluted samples separated on SDS-PAGE. (Bottom) β-galactosidase activity of each sample measured by FDG assay showing inhibition of WT sample compared to *Δgsn*. **(C)** Structure of LacZ-galactosyrin complex from Cryo-EM. The density map is shown for the top half of the structure and a fitted model is shown for the bottom half. Each monomer of LacZ tetramer is coloured differently. **(D)** Structure of galactosyrin revealed by Cryo-EM. Top: structures of LacZ-galactosyrin complex capture from WT or *Δgsn* strains and of LacZ incubated with synthetic galactosyrin. Density maps with fitted protein structures show the enzyme active site with the presence and absence of galactosyrin (orange). The resolution of each structure is shown in brackets. Bottom: extracted density map with fitted structure of galactosyrin from top and side view. **(E)** Galactosyrin mimics galactose binding in the active site. Overlay of structures of the LacZ active site and interacting residues in complex with galactosyrin (orange) or galactose (blue) showing similarity of overall binding pose and positioning of hydroxyl groups. The positive charge on the likely protonated amine nitrogen of galactosyrin can introduce extra electrostatic interaction with the negatively charged catalytic glutamic acid (E538) and cation-pi interaction with the aromatic tryptophan (W569). The stick representation of the molecular structure is coloured by heteroatoms (red:oxygen, blue:nitrogen) while hydrogen is not shown. The green sphere represents Mg2^+^ and the purple sphere represents Na^+^. Dashed lines represent hydrogen bonds.

To elucidate the molecular structure of galactosyrin, we used His-tagged LacZ immobilised on a metal affinity resin to capture galactosyrin from the crude secretome of the WT strain until LacZ saturation. Subsequent washing and elution with imidazole yielded a LacZ-galactosyrin complex with a high degree of inhibitor saturation (Fig 2A, B). Using cryo-electron microscopy (cryo-EM), we resolved the structure of the LacZ-galactosyrin complex at 1.9 Å resolution and detected an electron density in the active site that was absent in the negative control generated using the secretome of the Δ*gsn* mutant (Fig. 2C, D, S5). This density revealed that galactosyrin consists of a five-membered ring with three chiral centers: two with putative hydroxyls and one with a putative branching geminal diol group, which likely forms by hydration of an aldehyde group (Fig. 2D). We next chemically synthesised this molecule (Fig. S13) and obtained an unprecedented 1.4 Å resolution structure of its complex with LacZ, which is identical to the native galactosyrin (Fig. 2D, S5), confirming the structure of galactosyrin. To the best of our knowledge, this iminosugar has not been observed and characterized before and illustrates that cryo-EM can be used to elucidate structures of novel natural products bound to their targets at atomic resolution.

To further validate the structure, we analysed soluble metabolites extracted from the captured LacZ-galactosyrin complex with gas chromatography-mass spectrometry (GC-MS) after chemical modifications to enable carbohydrate analysis. The peaks of the synthetic galactosyrin standard are identical to those detected in native galactosyrin and absent in the *Δgsn*-derived sample (Fig. S6A). These mass spectra are consistent with the identified structure (Fig. S6A, S9). We also detected the same MS signals in apoplastic fluid extracted from *N. benthamiana* leaves infected with WT but not *Δgsn* mutant *P. syringae* (Fig. S6B).

The LacZ-galactosyrin complex structure also revealed the inhibition mechanism. Galactosyrin binds to the enzyme active site and closely mimics the orientation of hydroxyl groups of galactose, the natural target of LacZ (Fig. 2E)(10). Remarkably, the branching geminal diol group allows the five-membered galactosyrin ring to mimic the conformation of the six-membered galactose ring. Additionally, the nitrogen of galactosyrin is likely protonated, resulting in a positive charge that electrostatically interacts with the catalytic glutamic acid (E538), and establishes a cation-pi interaction with the aromatic tryptophan (W569) (Fig. 2E). Indeed, the synthetic galactosyrin is a potent inhibitor of both LacZ from *E. coli* and BGAL1 from *N. benthamiana*, with an IC_50_ below that of 1-deoxy-galactonojirimycin and similar to galactostatin, two well-known iminosugars with 6-membered rings (Fig. S7).

### Galactosyrin is produced from a purine pathway intermediate through three enzymes and chemical conversion

To resolve the biosynthesis pathway of galactosyrin, we considered its structure and the putative functions of the three biosynthesis enzymes encoded by the *gsn* cluster. We first focused on GsnB as it is unique to the *gsn* cluster in our comparative genomics analysis (Fig. S4). GsnB is homologous to RibD reductase, which functions in the riboflavin synthesis pathway (Fig. S8A). The Alphafold2-predicted structure of GsnB contains conserved active site pockets similar to those in the crystal structures of RibD in complex with the substrate analog ribose-5-phosphate (R5P) and cofactor NADPH (11) (Fig. S8B), suggesting that GsnB could act on a similar substrate. This GsnB substrate likely also contains an amine group since the *gsn* cluster lacks an aminotransferase, unlike other clusters containing GsnA homologs (Fig. S4).

**Fig. 3.**
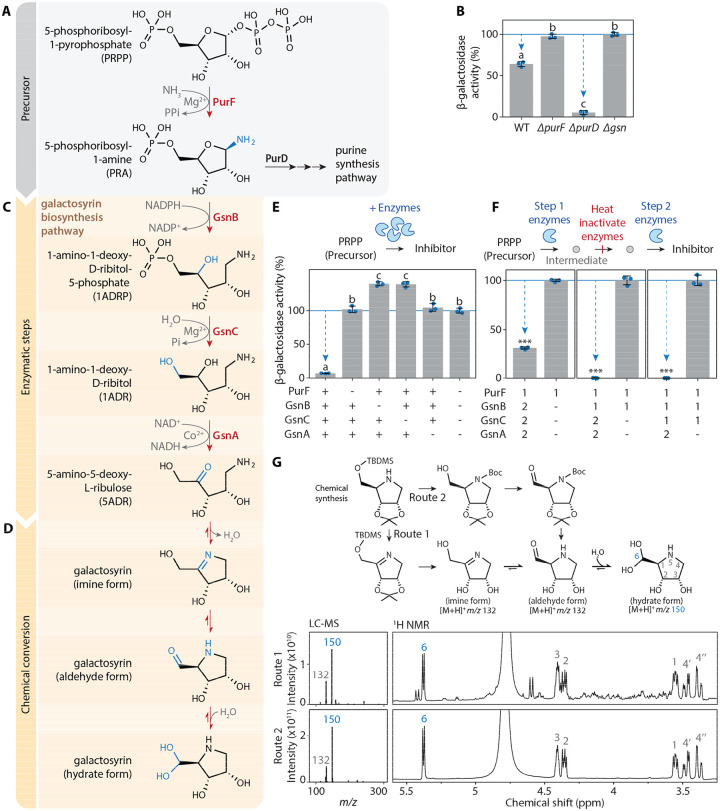
Galactosyrin biosynthesis branches off from purine biosynthesis by enzymatic and chemical conversion. **(A)** Galactosyrin biosynthesis branches off from the purine biosynthesis pathway. **(B)** *purF* but not *purD* is required for galactosyrin biosynthesis. Bacterial strains (WT or knockout mutants *ΔpurF*, *ΔpurD*, *Δgsn*) were grown in virulence-inducing MG medium containing purines overnight then the supernatant was tested for inhibitor production. **(C)** Three *gsn*-encoded enzymes convert PRA into 5ADR. **(D)** A plausible 3-step chemical conversion pathway of 5ADR into the final hydrate form of galactosyrin. **(E)** PurF, GsnB, GsnC and GsnA are required and sufficient for the biosynthesis of galactosyrin from PRPP *in vitro*. Galactosyrin biosynthesis was reconstructed by mixing PRPP precursor with purified enzymes and their cofactors. For different mixtures, + indicates added enzymes, while - indicates omitted enzymes. **(F)** PurF, GsnB, GsnC and GsnA act consecutively in galactosyrin biosynthesis. Biosynthesis of galactosyrin was reconstructed by mixing PRPP precursor with purified enzymes and their cofactors in 2 separate steps: [1] enzymes added in the first step to produce an intermediate before heat inactivation of the enzymes, then [2] enzymes added in the second step to complete galactosyrin biosynthesis. **(B, E, F)** Inhibitor production was tested in an enzyme activity assay with FDG substrate and LacZ enzyme. β-galactosidase activity is reported as a percentage of the activity relative to the mean of no-inhibitor-control (*Δgsn* for B, all enzymes omitted for E, enzyme 2 omitted for F). Arrows highlight inhibition. Error bars represent standard deviation from 3 replicates. Different letters indicate different groups with statistically significant difference (P < 0.001) using one-way ANOVA and post-hoc Tukey HSD test (for B, E). Asterisks indicate statistically significant difference (P < 0.001) using Welch’s t-test (for F). **(G)** Both imine and aldehyde forms of galactosyrin spontaneously convert into the hydrate form in water. (Top) Chemical synthesis of galactosyrin using two routes, via imine or aldehyde forms. (Bottom) Products were analysed with liquid chromatography-mass spectrometry (LC-MS) (left) and H^1^-NMR (right), showing the spectra that correspond to the spontaneously formed hydrate form. Positions within the structure of the hydrate form are numbered and labelled on the corresponding signals in NMR spectra.

Considering that galactosyrin is a 5-carbon sugar-like molecule, we hypothesised that 5-phosphoribosyl-1-amine (PRA) might be a substrate of GsnB (Fig. 3A, S8C). PRA is produced by PurF from 5-phosphoribosyl-1-pyrophosphate (PRPP) and is used by PurD in purine synthesis (12). Indeed, when grown on purines to complement for purine deficiency, the *ΔpurF* mutant is unable to produce galactosyrin, unlike the *ΔpurD* mutant (Fig. 3B). The *ΔpurD* mutant possibly produces even more galactosyrin than WT bacteria because this mutation prevents PRA conversion through PurD (Fig. 3A, B). These findings establish PRA as the precursor for galactosyrin biosynthesis.

Given the reductase activity of RibD, we speculated that GsnB could similarly reduce PRA into 1-amino-1-deoxy-D-ribitol-5-phosphate (1ADRP) using cofactor NADPH (Fig. 3C). Subsequently, the putative phosphatase GsnC might remove the phosphate of 1ADRP to produce 1-amino-1-deoxy-D-ribitol (1ADR). Finally, the putative oxidase GsnA might oxidise the secondary hydroxyl in 1ADR to produce the ketose 5-amino-5-deoxy-L-ribulose (5ADR). 5ADR can spontaneously convert into the detected hydrated galactosyrin (Fig. 3D), as explained below.

To confirm enzymatic steps of galactosyrin biosynthesis, we produced purified enzymes (PurF, GsnB, GsnC and GsnA) and incubated them with PRPP precursor and cofactors. Galactosyrin was produced from the mixture with all four enzymes, demonstrating that these components are sufficient to produce galactosyrin *in vitro* (Fig. 3E). Omission of any of the four enzymes blocked galactosyrin production (Fig. 3E), demonstrating that each enzyme is required for galactosyrin biosynthesis.

To verify the order of these reactions, we first produced intermediates from each enzymatic step *in vitro* and heat-inactivated the enzymes. We were then able to produce galactosyrin from these intermediates by adding the subsequent enzymes and cofactors in the expected order (Fig. 3F). Furthermore, the intermediates (PRA, 1ADRP and 1ADR) were detected after each step by GC-MS and these intermediates were depleted upon the addition of subsequent enzymes (Fig. S10). To confirm the final enzymatic step, we also detected galactosyrin formation from synthetic 1ADR by GsnA (Fig. S11, S12B), an NAD^+^-dependent oxidase (Fig. S12C). Notably, the pink color of purified GsnA indicated that cobalt ion is a preferred cofactor, confirmed *in vitro* (Fig. S12D), unlike other alcohol dehydrogenases of Pfam family PF00107, which are zinc-dependent (13). Taken together, these results confirmed the biosynthesis pathway of galactosyrin (Fig. 3A).

Finally, we propose a plausible chemical conversion pathway for the final steps of galactosyrin formation (Fig. 3D). First, imine formation by the amine and ketone in 5ADR produced the imine form of galactosyrin. This imine substructure is known to be unstable in aqueous environment and we propose that it will undergo spontaneous chemical conversions, including Heyn’s rearrangement (14) that results in the aldehyde form of galactosyrin. This aldehyde can then be hydrated to yield the hydrate form (Fig. 3D). To confirm that this pathway occurs spontaneously, we chemically synthesised both the imine and aldehyde derivatives of galactosyrin (Fig. S13) and found that, upon protecting group cleavage in water, they both spontaneously convert into the hydrate form detectable by both LC-MS and NMR as the major product (Fig. 3G, S14). In addition, GC-MS analysis of galactosyrin produced *in vitro* by GsnA from 1ADR detected both imine and aldehyde forms of galactosyrin because GC-MS was performed in anhydrous conditions (Fig. S11). These results demonstrate that 5ADR undergoes spontaneous chemical conversions that ultimately yield the hydrate form of galactosyrin discovered in LacZ by Cryo-EM.

### Galactosyrin triggers the accumulation of extracellular glycoproteins and glycosides

**Fig. 4.**
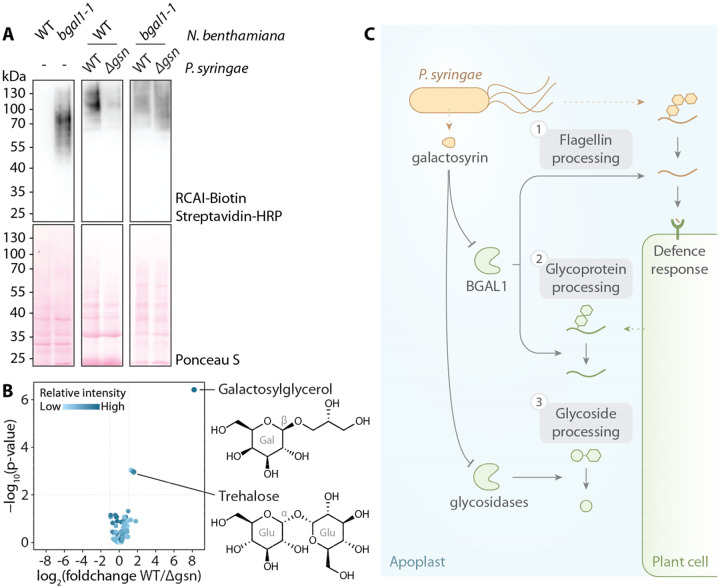
Galactosyrin manipulates multiple aspects of plant apoplast glycobiology. **(A)** Accumulation of RCAI-positive glycoproteins in the apoplast upon infection is dependent on BGAL1 and galactosyrin production. Proteins were extracted by acetone precipitation of apoplastic fluids from *N. benthamiana* (wild-type (WT) or BGAL1 knockout mutant (*bgal1-1*) with or without infection by *P. syringae* (WT or *Δgsn*), then separated on SDS-PAGE, blotted and probed with RCAI lectin targeting galactose. **(B)** Galactosylglycerol and trehalose accumulate in the apoplast during infection dependent on galactosyrin production. Volcano plot of soluble metabolites detected by GC-MS of apoplastic fluids from leaves infected with WT or *Δgsn* mutant (Table S2). Glucoside components are shown with galactose (Gal), Glucose (Glu) and bond configuration (α or β). **(C)** *P*. syringae produces galactosyrin to manipulate multiple aspects of glycobiology inside host plants by inhibiting plant glycosidases in the apoplast. [1] One major target of galactosyrin is the β-galactosidase BGAL1 previously shown to play a role in the processing of glycosylated flagellin to release plant defence elicitor (2). [2] Inhibition of BGAL1 by galactosyrin also interrupts apoplastic glycoprotein processing resulting in the accumulation of galactose-containing glycoproteins. [3] Galactosyrin also disrupts processing of glycosides by glycosidases other than BGAL1, resulting in the accumulation of galactosylglycerol and trehalose in the apoplast.

The originally identified target of galactosyrin is BGAL1, which acts in plant defence by facilitating the release of immunogenic peptides from glycosylated flagellin protein (2). We recently found that BGAL1 also removes terminal β-D-galactose from *N-* and *O*-glycans of transiently expressed recombinant proteins (15). Here, we found that BGAL1 can also process endogenous plant glycoproteins using RCAI, a terminal β-D-galactose-specific lectin, to probe the apoplastic proteome (Fig. 4A). Consequently, these RCAI-positive glycoproteins accumulated upon infection with WT *P. syringae* but not the *Δgsn* mutant in WT plants, while no differential accumulation of glycoproteins was observed in *bgal1-1* mutant plants (Fig. 4A), indicating that this modification of the host glycoproteome occurs through BGAL1 inhibition by galactosyrin. Moreover, untargeted metabolomics of apoplastic fluid from plants infected with WT and *Δgsn P. syringae* revealed that galactosyrin also triggers the accumulation of 873 ± 150 μM galactosylglycerol and 46 ± 12 μM trehalose in the apoplast of WT *P. syringae*-infected leaves (Fig. 4B, S15). The accumulation of these metabolites was independent of BGAL1 (Fig. S15A), suggesting that galactosyrin also targets other glycosidases that are involved in glycoside processing. Indeed, galactosyrin can inhibit several other glycosidases, including other β-galactosidases in *N. benthamiana* (Fig. S16) and α- and β-glucosidases (Fig. S17). Taken together, these findings demonstrate that galactosyrin is a multifunctional novel iminosugar produced by *P. syringae* to manipulate different plant glycosidases to influence various aspects of glycobiology in the plant apoplast during infection (Fig. 4C).

## DISCUSSION

We have elucidated the structure and biosynthesis pathway of galactosyrin, a novel iminosugar secreted by the model plant pathogen *P. syringae*. We discovered galactosyrin structure and its mode of action by solving the cryo-EM structure of inhibitor-bound β-galactosidase complex at an unprecedented atomic resolution (Fig. 2). Besides inhibiting BGAL1 to avoid the release of immunogenic flagellin fragments, we discovered that galactosyrin also manipulates the glycoproteome and metabolome in the apoplast of infected plants.

The biosynthesis pathway of galactosyrin is unique among iminosugars because it does not involve an aminotransferase but instead coopts an intermediate from the purine biosynthesis pathway using the NADPH-dependent reductase GsnB. Biosynthesis of 1-deoxynojirimycin (DNJ), nectrisine and 1,4-dideoxy-1,4-imino-arabinitol (DAB-1) all start with an aminotransferase acting on a sugar-phosphate precursor (8, 9, 16, 17). However, all known iminosugar biosynthesis pathways involve an oxidase (GsnA homolog) to convert a hydroxyl group into a carbonyl group that then reacts with an amine group to form the iminosugar ring. Comparative genomic analysis of GsnA (Fig. S4) identified several biosynthesis gene clusters in bacterial species that are likely to produce novel iminosugars that remain to be characterized. Although hundreds of iminosugars isolated as natural products and thousands of synthetic analogues have been studied (7), galactosyrin is the first iminosugar with an aldehyde substituent attached to the heterocyclic ring. The hydrated form of the aldehyde is stable and constitutes a new class of iminosugars. We propose that this unique structure of galactosyrin is formed from an imine bond, which is then rearranged into an aldehyde form, followed by its hydration, thereby forming a branching geminal diol group, which is uniquely stable, even in water (Fig. 3). This property allows galactosyrin to accomodate a configuration that efficiently mimics galactose when bound to the active site of β-galactosidase. This discovery expands the diversity of iminosugars and initiates the exploration of a new class of galactosyrin-like iminosugars with different specificity or affinity. These new iminosugars might include important future pharmaceuticals because of their affinity to a wide range of carbohydrate active enzymes. Miglitol and DGJ, for instance, are iminosugars used to treat type-II diabetes and Fabry disease, respectively (18, 19).

Galactosyrin is a multifunctional virulence factor produced by *P. syringae* during infection. First, galactosyrin inhibits BGAL1, which was previously shown to function in plant immunity by promoting the hydrolytic release of immunogenic fragments from glycosylated bacterial flagellin (2). Second, we discovered that the inhibition of BGAL1 also results in an accumulation of galactose-containing glycoproteins during infection with *P. syringae*. Alterations of glycoproteomes during infection by *P. syringae* have also been reported (20, 21). We previously found that BGAL1 removes the terminal β-D-galactose residues of both *N*- and *O*-glycans on recombinant glycoproteins (15). Galactose is also a common monosaccharide in endogenous glycans of *N*- and *O*-glycosylated proteins such as cell surface receptor kinases and defence-related arabinogalactan proteins (AGPs) (22). At this stage, it is unclear how the manipulated glycoproteome could affect the bacterial colonization but the loss of apoplastic β-galactosidase activity was also reported to impact cell wall glycan processing and functions, such as interactions and hydration properties (23–25).

We also discovered that galactosyrin induces an accumulation of galactosylglycerol and trehalose in the apoplast of infected plants, which is independent from BGAL1 and possibly results from galactosyrin inhibiting other glycosidases. Degradation of glycosides by glycosidases could therefore be another mechanism to modulate solute levels in the apoplast. Although pathogens often induce accumulation of sugars and metabolites in the apoplast through production or efflux to provide nutrient sources (26, 27), trehalose and galactosylglycerol are not consumed by *P. syringae in vitro* (Fig. S15D). On the other hand, these glycosides are well-known osmolytes (28, 29) and may therefore contribute to the establishment of aqueous apoplast conditions that promotes virulence (30, 31). Accumulation of glycosides might also influence bacterial colonisation in different ways. Elevated trehalose levels, for instance, dampens plant defence responses and promotes *P. syringae* infection (32). In addition, by inhibiting β-glucosidases, galactosyrin may also prevent the activation of glucosides that act in plant defence and signaling (33).

The presence of homologous iminosugar biosynthesis gene clusters in plant pathogens such as *Acidovorax* and *Erwinia*, and plant-associated bacteria such as *Kosakonia*, *Bacillus* and *Paenibacillus* indicates that the use of iminosugars to manipulate the glycobiology of the host plant might be a common strategy used by plant-associated bacterial pathogens and symbionts.

## Supplementary Material

1

## Data Availability

All data are available in the manuscript, supplementary materials and cited references. The cryoEM density maps and corresponding atomic models have been deposited in the EMDB and PDB, respectively. The accession codes are: for LacZ with native inhibitor (WT), EMDB-19182 and PDB 8RI7; for LacZ with *Δgsn* negative control, EMDB-19181 and PDB 8RI6; for LacZ with synthetic galactosyrin EMDB-19183 and PDB 8RI8.

## References

[1] Doehlemann G., Hemetsberger C., Apoplastic immunity and its suppression by filamentous plant pathogens. New Phytol. 198, 1001–1016 (2013). 23594392 10.1111/nph.12277

[2] Buscaill P., Chandrasekar B., Sanguankiattichai N., Kourelis J., Kaschani F., Thomas E. L., Morimoto K., Kaiser M., Preston G. M., Ichinose Y., van der Hoorn R. A. L., Glycosidase and glycan polymorphism control hydrolytic release of immunogenic flagellin peptides. Science (80-.). 364 (2019). 10.1126/science.aav0748 30975858

[3] Wei C., Kvitko B. H., Shimizu R., Crabill E., Alfano J. R., Lin N., Martin G. B., Huang H., Collmer A., A *Pseudomonas syringae* pv. *tomato* DC3000 mutant lacking the type III effector HopQ1–1 is able to cause disease in the model plant Nicotiana benthamiana. Plant J. 51, 32–46 (2007). 17559511 10.1111/j.1365-313X.2007.03126.x

[4] Vencato M., Tian F., Alfano J. R., Buell C. R., Cartinhour S., DeClerck G. A., Guttman D. S., Stavrinides J., Joardar V., Lindeberg M., Bronstein P. A., Mansfield J. W., Myers C. R., Collmer A., Schneider D. J., Bioinformatics-enabled identification of the HrpL regulon and type III secretion system effector proteins of *Pseudomonas syringae* pv. *phaseolicola* 1448A. Mol. Plant-Microbe Interact. 19, 1193–1206 (2006). 17073302 10.1094/MPMI-19-1193

[5] Xie Y., Shao X., Deng X., Regulation of type III secretion system in *Pseudomonas syringae*. Environ. Microbiol. 21, 4465–4477 (2019). 31408268 10.1111/1462-2920.14779

[6] Williams K. P., Integration sites for genetic elements in prokaryotic tRNA and tmRNA genes: Sublocation preference of integrase subfamilies. Nucleic Acids Res. 30, 866–875 (2002). 11842097 10.1093/nar/30.4.866 PMC100330

[7] Watson A. A., Fleet G. W. J., Asano N., Molyneux R. J., Nash R. J., Polyhydroxylated alkaloids - Natural occurrence and therapeutic applications. Phytochemistry 56, 265–295 (2001). 11243453 10.1016/s0031-9422(00)00451-9

[8] Nuñez C., Horenstein N. A., Functional analysis of a gene cluster from *Chitinophaga pinensis* involved in biosynthesis of the pyrrolidine azasugar DAB-1. J. Nat. Prod. 82, 3401–3409 (2019). 31793783 10.1021/acs.jnatprod.9b00758

[9] Clark L. F., Johnson J. V., Horenstein N. A., Identification of a gene cluster that initiates azasugar biosynthesis in *Bacillus amyloliquefaciens*. ChemBioChem 12, 2147–2150 (2011). 21786380 10.1002/cbic.201100347

[10] Juers D. H., Heightman T. D., Vasella A., McCarter J. D., Mackenzie L., Withers S. G., Matthews B. W., A structural view of the action of *Escherichia coli* (lacZ) β-galactosidase. Biochemistry 40, 14781–14794 (2001). 11732897 10.1021/bi011727i

[11] Stenmark P., Moche M., Gurmu D., Nordlund P., The crystal structure of the bifunctional deaminase/reductase RibD of the riboflavin biosynthetic pathway in *Escherichia coli*: implications for the reductive mechanism. J. Mol. Biol. 373, 48–64 (2007). 17765262 10.1016/j.jmb.2006.12.009

[12] Zhang Y., Morar M., Ealick S. E., Structural biology of the purine biosynthetic pathway. Cell. Mol. Life Sci. 65, 3699–3724 (2008). 18712276 10.1007/s00018-008-8295-8 PMC2596281

[13] Mistry J., Chuguransky S., Williams L., Qureshi M., Salazar G. A., Sonnhammer E. L. L., Tosatto S. C. E., Paladin L., Raj S., Richardson L. J., Finn R. D., Bateman A., Pfam: The protein families database in 2021. Nucleic Acids Res. 49, D412–D419 (2021). 33125078 10.1093/nar/gkaa913 PMC7779014

[14] Wang Z., “Heyns Rearrangement” in Comprehensive Organic Name Reactions and Reagents (2010).

[15] Kriechbaum R., Ziaee E., Grünwald-Gruber C., Buscaill P., van der Hoorn R. A. L., Castilho A., BGAL1 depletion boosts the level of β-galactosylation of N- and O-glycans in *N. benthamiana*. Plant Biotechnol. J. 18, 1537–1549 (2020). 31837192 10.1111/pbi.13316 PMC7292537

[16] Miyauchi R., Ono C., Ohnuki T., Shiba Y., Nectrisine biosynthesis genes in *Thelonectria discophora* SANK 18292: Identification and functional analysis. Appl. Environ. Microbiol. 82, 6414–6422 (2016). 27565616 10.1128/AEM.01709-16 PMC5066358

[17] Beal H. E., Horenstein N. A., Comparative genomic analysis of azasugar biosynthesis. AMB Express 11 (2021). 10.1186/s13568-021-01279-5 PMC8382821 34424396

[18] Nash R. J., Kato A., Yu C. Y., Fleet G. W., Iminosugars as therapeutic agents: Recent advances and promising trends. Future Med. Chem. 3, 1513–1521 (2011). 21882944 10.4155/fmc.11.117

[19] Winchester B. G., Iminosugars: from botanical curiosities to licensed drugs. Tetrahedron Asymmetry 20, 645–651 (2009).

[20] Kim S. J., Bhandari D. D., Sokoloski R., Brandizzi F., Immune activation during *Pseudomonas* infection causes local cell wall remodeling and alters AGP accumulation. Plant J. 116, 541–557 (2023). 37496362 10.1111/tpj.16393

[21] Beihammer G., Romero-Pérez A., Maresch D., Figl R., Mócsai R., Grünwald-Gruber C., Altmann F., Van Damme E. J. M., Strasser R., *Pseudomonas syringae* DC3000 infection increases glucosylated N-glycans in Arabidopsis thaliana. Glycoconj. J. 40, 97–108 (2023). 36269466 10.1007/s10719-022-10084-6 PMC9925501

[22] Nguema-Ona E., Vicré-Gibouin M., Gotté M., Plancot B., Lerouge P., Bardor M., Driouich A., Cell wall O-glycoproteins and N-glycoproteins: Aspects of biosynthesis and function. Front. Plant Sci. 5, 1–12 (2014). 10.3389/fpls.2014.00499 PMC4183102 25324850

[23] Sampedro J., Gianzo C., Iglesias N., Guitián E., Revilla G., Zarra I., AtBGAL10 is the main xyloglucan β-galactosidase in arabidopsis, and its absence results in unusual xyloglucan subunits and growth defects. Plant Physiol. 158, 1146–1157 (2012). 22267505 10.1104/pp.111.192195 PMC3291251

[24] Kotake T., Dina S., Konishi T., Molecular cloning of a β-galactosidase from radish that specifically hydrolyzes β-(1-> 3)-and β-(1->6)-galactosyl residues of arabinogalactan protein. Plant Physiol. 138, 1563–1576 (2005). 15980190 10.1104/pp.105.062562 PMC1176426

[25] Dean G. H., Zheng H., Tewari J., Huang J., Young D. S., Yeen T. H., Western T. L., Carpita N. C., McCann M. C., Mansfield S. D., Haughn G. W., The Arabidopsis MUM2 gene encodes a β-galactosidase required for the production of seed coat mucilage with correct hydration properties. Plant Cell 19, 4007–4021 (2007). 18165329 10.1105/tpc.107.050609 PMC2217648

[26] El Kasmi F., Horvath D., Lahaye T., Microbial effectors and the role of water and sugar in the infection battle ground. Curr. Opin. Plant Biol. 44, 98–107 (2018). 29597139 10.1016/j.pbi.2018.02.011

[27] Zhu X., Fang D., Li D., Zhang J., Jiang H., Guo L., He Q., Zhang T., Macho A. P., Wang E., Shen Q. H., Wang Y., Zhou J. M., Ma W., Qiao Y., *Phytophthora sojae* boosts host trehalose accumulation to acquire carbon and initiate infection. Nat. Microbiol. 8, 1561–1573 (2023). 37386076 10.1038/s41564-023-01420-z

[28] Freeman B. C., Chen C., Beattie G. A., Identification of the trehalose biosynthetic loci of *Pseudomonas syringae* and their contribution to fitness in the phyllosphere. Environ. Microbiol. 12, 1486–1497 (2010). 20192963 10.1111/j.1462-2920.2010.02171.x

[29] Pade N., Linka N., Ruth W., Weber A. P. M., Hagemann M., Floridoside and isofloridoside are synthesized by trehalose 6-phosphate synthase-like enzymes in the red alga *Galdieria sulphuraria*. New Phytol. 205, 1227–1238 (2015). 25323590 10.1111/nph.13108

[30] Roussin-Léveillée C., Mackey D., Ekanayake G., Gohmann R., Moffett P., Extracellular niche establishment by plant pathogens. Nat. Rev. Microbiol., doi: 10.1038/s41579-023-00999-8 (2024). PMC11593749 38191847

[31] Xin X. F., Kvitko B., He S. Y., *Pseudomonas syringae*: What it takes to be a pathogen. Nat. Rev. Microbiol. 16, 316–328 (2018). 29479077 10.1038/nrmicro.2018.17 PMC5972017

[32] Wang X., Du Y., Yu D., Trehalose phosphate synthase 5-dependent trehalose metabolism modulates basal defense responses in *Arabidopsis thaliana*. J. Integr. Plant Biol. 61, 509–527 (2019). 30058771 10.1111/jipb.12704

[33] Morant A. V., Jørgensen K., Jørgensen C., Paquette S. M., Sánchez-Pérez R., Møller B. L., Bak S., β-Glucosidases as detonators of plant chemical defense. Phytochemistry 69, 1795–1813 (2008). 18472115 10.1016/j.phytochem.2008.03.006

[34] Fiehn O., Metabolomics by Gas Chromatography–Mass Spectrometry: Combined Targeted and Untargeted Profiling. Curr. Protoc. Mol. Biol. 114 (2016). 10.1002/0471142727.mb3004s114 PMC4829120 27038389

[35] Lai Z., Fiehn O., Mass spectral fragmentation of trimethylsilylated small molecules. Mass Spectrom. Rev. 37, 245–257 (2018). 27580014 10.1002/mas.21518

[36] Harvey D. J., Vouros P., Mass spectrometric fragmentation of trimethylsilyl and related alkylsilyl derivatives. Mass Spectrom. Rev. 39, 105–211 (2020). 31808199 10.1002/mas.21590

[37] Kamath V. P., Xue J., Juarez-Brambila J. J., Morris C. B., Ganorkar R., Morris P. E., Synthesis of analogs of forodesine HCl, a human purine nucleoside phosphorylase inhibitor—Part I. Bioorg. Med. Chem. Lett. 19, 2624–2626 (2009). 19386498 10.1016/j.bmcl.2009.04.017

[38] Bronstein P. A., Filiatrault M. J., Myers C. R., Rutzke M., Schneider D. J., Cartinhour S. W., Global transcriptional responses of *Pseudomonas syringae* DC3000 to changes in iron bioavailability *in vitro*. BMC Microbiol. 8, 1–15 (2008). 19055731 10.1186/1471-2180-8-209 PMC2613906

[39] Winsor G. L., Griffiths E. J., Lo R., Dhillon B. K., Shay J. A., Brinkman F. S. L., Enhanced annotations and features for comparing thousands of *Pseudomonas* genomes in the *Pseudomonas* genome database. Nucleic Acids Res. 44, D646–D653 (2016). 26578582 10.1093/nar/gkv1227 PMC4702867

[40] Schindelin J., Arganda-Carreras I., Frise E., Kaynig V., Longair M., Pietzsch T., Preibisch S., Rueden C., Saalfeld S., Schmid B., Tinevez J.-Y., White D. J., Hartenstein V., Eliceiri K., Tomancak P., Cardona A., Fiji: an open-source platform for biological-image analysis. Nat. Methods 9, 676–682 (2012). 22743772 10.1038/nmeth.2019 PMC3855844

[41] Kvitko B. H., Collmer A., “Construction of *Pseudomonas syringae* pv. *tomato* DC3000 mutant and polymutant strains” in Methods in Molecular Biology (Clifton, N.J.) (2011), pp. 109–128. 10.1007/978-1-61737-998-7_10 21359804

[42] Schäfer A., Tauch A., Jäger W., Kalinowski J., Thierbach G., Pühler A., Small mobilizable multi-purpose cloning vectors derived from the *Escherichia coli* plasmids pK18 and pK19: selection of defined deletions in the chromosome of *Corynebacterium glutamicum*. Gene 145, 69–73 (1994). 8045426 10.1016/0378-1119(94)90324-7

[43] Obranić S., Babić F., Maravić-Vlahoviček G., Improvement of pBBR1MCS plasmids, a very useful series of broad-host-range cloning vectors. Plasmid 70, 263–267 (2013). 23583732 10.1016/j.plasmid.2013.04.001

[44] Soldan R., Sanguankiattichai N., Bach-Pages M., Bervoets I., Huang W. E., Preston G. M., From macro to micro: a combined bioluminescence-fluorescence approach to monitor bacterial localization. Environ. Microbiol. 23, 2070–2085 (2021). 33103833 10.1111/1462-2920.15296 PMC8614114

[45] Katoh K., Standley D. M., MAFFT multiple sequence alignment software version 7: Improvements in performance and usability. Mol. Biol. Evol. 30, 772–780 (2013). 23329690 10.1093/molbev/mst010 PMC3603318

[46] Minh B. Q., Schmidt H. A., Chernomor O., Schrempf D., Woodhams M. D., von Haeseler A., Lanfear R., IQ-TREE 2: new models and efficient methods for phylogenetic inference in the genomic era. Mol. Biol. Evol. 37, 1530–1534 (2020). 32011700 10.1093/molbev/msaa015 PMC7182206

[47] Kalyaanamoorthy S., Minh B. Q., Wong T. K. F., von Haeseler A., Jermiin L. S., ModelFinder: fast model selection for accurate phylogenetic estimates. Nat. Methods 14, 587–589 (2017). 28481363 10.1038/nmeth.4285 PMC5453245

[48] Letunic I., Bork P., Interactive Tree Of Life (iTOL) v5: an online tool for phylogenetic tree display and annotation. Nucleic Acids Res. 49, W293–W296 (2021). 33885785 10.1093/nar/gkab301 PMC8265157

[49] Camacho C., Coulouris G., Avagyan V., Ma N., Papadopoulos J., Bealer K., Madden T. L., BLAST+: architecture and applications. BMC Bioinformatics 10, 421 (2009). 20003500 10.1186/1471-2105-10-421 PMC2803857

[50] Agarwala R., Barrett T., Beck J., Benson D. A., Bollin C., Bolton E., Bourexis D., Brister J. R., Bryant S. H., Canese K., Charowhas C., Clark K., Dicuccio M., Dondoshansky I., Federhen S., Feolo M., Funk K., Geer L. Y., Gorelenkov V., Hoeppner M., Holmes B., Johnson M., Khotomlianski V., Kimchi A., Kimelman M., Kitts P., Klimke W., Krasnov S., Kuznetsov A., Landrum M. J., Landsman D., Lee J. M., Lipman D. J., Lu Z., Madden T. L., Madej T., Marchler-Bauer A., Karsch-Mizrachi I., Murphy T., Orris R., Ostell J., O’sullivan C., Panchenko A., Phan L., Preuss D., Pruitt K. D., Rodarmer K., Rubinstein W., Sayers E., Schneider V., Schuler G. D., Sherry S. T., Sirotkin K., Siyan K., Slotta D., Soboleva A., Soussov V., Starchenko G., Tatusova T. A., Todorov K., Trawick B. W., Vakatov D., Wang Y., Ward M., Wilbur W. J., Yaschenko E., Zbicz K., Database resources of the National Center for Biotechnology Information. Nucleic Acids Res. 44, D7–D19 (2016). 26615191 10.1093/nar/gkv1290 PMC4702911

[51] Thakur S., Weir B. S., Guttman D., Phytopathogen genome announcement: Draft genome sequences of 62 Pseudomonas syringae type and pathotype strains. Mol. Plant-Microbe Interact. 29, MPMI-01-16-0013-TA (2016). 10.1094/MPMI-01-16-0013-TA 26883489

[52] Baltrus D. A., McCann H. C., Guttman D. S., Evolution, genomics and epidemiology of *Pseudomonas syringae*: Challenges in Bacterial Molecular Plant Pathology. Mol. Plant Pathol. 18, 152–168 (2017). 27798954 10.1111/mpp.12506 PMC6638251

[53] O’Leary N. A., Wright M. W., Brister J. R., Ciufo S., Haddad D., McVeigh R., Rajput B., Robbertse B., Smith-White B., Ako-Adjei D., Astashyn A., Badretdin A., Bao Y., Blinkova O., Brover V., Chetvernin V., Choi J., Cox E., Ermolaeva O., Farrell C. M., Goldfarb T., Gupta T., Haft D., Hatcher E., Hlavina W., Joardar V. S., Kodali V. K., Li W., Maglott D., Masterson P., McGarvey K. M., Murphy M. R., O’Neill K., Pujar S., Rangwala S. H., Rausch D., Riddick L. D., Schoch C., Shkeda A., Storz S. S., Sun H., Thibaud-Nissen F., Tolstoy I., Tully R. E., Vatsan A. R., Wallin C., Webb D., Wu W., Landrum M. J., Kimchi A., Tatusova T., DiCuccio M., Kitts P., Murphy T. D., Pruitt K. D., Reference sequence (RefSeq) database at NCBI: current status, taxonomic expansion, and functional annotation. Nucleic Acids Res. 44, D733–D745 (2016). 26553804 10.1093/nar/gkv1189 PMC4702849

[54] Lu S., Wang J., Chitsaz F., Derbyshire M. K., Geer R. C., Gonzales N. R., Gwadz M., Hurwitz D. I., Marchler G. H., Song J. S., Thanki N., Yamashita R. A., Yang M., Zhang D., Zheng C., Lanczycki C. J., Marchler-Bauer A., CDD/SPARCLE: the conserved domain database in 2020. Nucleic Acids Res. 48, D265–D268 (2020). 31777944 10.1093/nar/gkz991 PMC6943070

[55] Altschul S., Gapped BLAST and PSI-BLAST: a new generation of protein database search programs. Nucleic Acids Res. 25, 3389–3402 (1997). 9254694 10.1093/nar/25.17.3389 PMC146917

[56] Zheng S. Q., Palovcak E., Armache J.-P., Verba K. A., Cheng Y., Agard D. A., MotionCor2: anisotropic correction of beam-induced motion for improved cryo-electron microscopy. Nat. Methods 14, 331–332 (2017). 28250466 10.1038/nmeth.4193 PMC5494038

[57] Rohou A., Grigorieff N., CTFFIND4: Fast and accurate defocus estimation from electron micrographs. J. Struct. Biol. 192, 216–221 (2015). 26278980 10.1016/j.jsb.2015.08.008 PMC6760662

[58] Wagner T., Merino F., Stabrin M., Moriya T., Antoni C., Apelbaum A., Hagel P., Sitsel O., Raisch T., Prumbaum D., Quentin D., Roderer D., Tacke S., Siebolds B., Schubert E., Shaikh T. R., Lill P., Gatsogiannis C., Raunser S., SPHIRE-crYOLO is a fast and accurate fully automated particle picker for cryo-EM. Commun. Biol. 2, 218 (2019). 31240256 10.1038/s42003-019-0437-z PMC6584505

[59] Zivanov J., Nakane T., Forsberg B. O., Kimanius D., Hagen W. J., Lindahl E., Scheres S. H., New tools for automated high-resolution cryo-EM structure determination in RELION-3. Elife 7 (2018). 10.7554/eLife.42166 PMC6250425 30412051

[60] Jamali K., Käll L., Zhang R., Brown A., Kimanius D., Scheres S. H. W., Automated model building and protein identification in cryo-EM maps. (2023). 10.1101/2023.05.16.541002. PMC11006616 38408488

[61] Emsley P., Cowtan K., Coot : model-building tools for molecular graphics. Acta Crystallogr. Sect. D Biol. Crystallogr. 60, 2126–2132 (2004). 15572765 10.1107/S0907444904019158

[62] Adams P. D., Afonine P. V., Bunkóczi G., Chen V. B., Davis I. W., Echols N., Headd J. J., Hung L.-W., Kapral G. J., Grosse-Kunstleve R. W., McCoy A. J., Moriarty N. W., Oeffner R., Read R. J., Richardson D. C., Richardson J. S., Terwilliger T. C., Zwart P. H., PHENIX : a comprehensive Python-based system for macromolecular structure solution. Acta Crystallogr. Sect. D Biol. Crystallogr. 66, 213–221 (2010). 20124702 10.1107/S0907444909052925 PMC2815670

[63] Mirdita M., Schütze K., Moriwaki Y., Heo L., Ovchinnikov S., Steinegger M., ColabFold: making protein folding accessible to all. Nat. Methods 19, 679–682 (2022). 35637307 10.1038/s41592-022-01488-1 PMC9184281

[64] Pettersen E. F., Goddard T. D., Huang C. C., Meng E. C., Couch G. S., Croll T. I., Morris J. H., Ferrin T. E., UCSF ChimeraX: Structure visualization for researchers, educators, and developers. Protein Sci. 30, 70–82 (2021). 32881101 10.1002/pro.3943 PMC7737788

[65] Ritz C., Baty F., Streibig J. C., Gerhard D., Dose-response analysis using R. PLoS One 10, e0146021 (2015). 26717316 10.1371/journal.pone.0146021 PMC4696819

[66] Stein S. E., Linstrom P., Mirokhin Y., Tchekhovskoi D., Yang X., Gary W., Sparkman M. O. D., “NIST Standard Reference Database 1A (NIST 08)” (2011).

[67] Kopka J., Schauer N., Krueger S., Birkemeyer C., Usadel B., Bergmuller E., Dormann P., Weckwerth W., Gibon Y., Stitt M., Willmitzer L., Fernie A. R., Steinhauser D., GMD@CSB.DB: the Golm Metabolome Database. Bioinformatics 21, 1635–1638 (2005). 15613389 10.1093/bioinformatics/bti236

[68] O’Leary B. M., Rico A., McCraw S., Fones H. N., Preston G. M., The infiltration-centrifugation technique for extraction of apoplastic fluid from plant leaves using Phaseolus vulgaris as an example. J. Vis. Exp., doi: 10.3791/52113 (2014). PMC4396939 25549068

[69] Kessler M., Acuto O., Storelli C., Murer H., Müller M., Semenza G., A modified procedure for the rapid preparation of efficiently transporting vesicles from small intestinal brush border membranes. Their use in investigating some properties of d-glucose and choline transport systems. Biochim. Biophys. Acta - Biomembr. 506, 136–154 (1978). 10.1016/0005-2736(78)90440-6 620021

[70] Parales R. E., Harwood C. S., Construction and use of a new broad-host-range lacZ transcriptional fusion vector, pHRP309, for Gram - bacteria. Gene 133, 23–30 (1993). 8224891 10.1016/0378-1119(93)90220-w

[71] Figurski D. H., Helinski D. R., Replication of an origin-containing derivative of plasmid RK2 dependent on a plasmid function provided in trans. Proc. Nati. Acad. Sc 76, 1648–1652 (1979). 10.1073/pnas.76.4.1648 PMC383447 377280

[72] Fones H., Davis C. A. R., Rico A., Fang F., Smith J. A. C., Preston G. M., Metal Hyperaccumulation Armors Plants against Disease. PLOS Pathog. 6, e1001093 (2010). 20838462 10.1371/journal.ppat.1001093 PMC2936542

[73] Bao Y., Lies D. P., Fu H., Roberts G. P., An improved Tn7-based system for the single-copy insertion of cloned genes into chromosomes of gram-negative bacteria. Gene 109, 167–168 (1991). 1661697 10.1016/0378-1119(91)90604-a

