# Supplementary material for: Bacterial pathogen deploys iminosugar galactosyrin to manipulate plant glycobiology
Source: bioRxiv. 2025 Feb 14:2025.02.13.638044. Preprint. [Version 1] doi: 10.1101/2025.02.13.638044 (PMC11844564; doi:10.1101/2025.02.13.638044)
Supplement: 1 [file NIHPP2025.02.13.638044V1-supplement-1.pdf]

bioRxiv preprint doi: https://doi.org/10.1101/2025.02.13.638044; this version posted February 14, 2025. The copyright holder for this preprint (which was not certified by peer review) is the author/funder, who has granted bioRxiv a license to display the preprint in perpetuity. It is made available under aCC-BY 4.0 International license.

Supplementary Information

bioRxiv preprint doi: https://doi.org/10.1101/2025.02.13.638044; this version posted February 14, 2025. The copyright holder for this preprint (which was not certified by peer review) is the author/funder, who has granted bioRxiv a license to display the preprint in perpetuity. It is made available under aCC-BY 4.0 International license.

Materials and Methods

References (34-73)

Data S1 to S2

Fig. S1. Validation screen for candidate galactosyrin mutants.

Fig. S2. Deletion of the *gsn* cluster does not affect bacterial growth *in vitro*.

Fig. S3. The distribution of the *gsn* cluster among *P. syringae* strains is indicative of horizontal gene transfer.

Fig. S4. *gsnA* homologs are part of different putative iminosugar biosynthesis gene clusters.

Fig. S5. Cryo-EM structure determination of β-galactosidase (LacZ) bound with galactosyrin.

Fig. S6. Galactosyrin was detected in enzyme-inhibitor complex and apoplast of infected plants.

Fig. S7. Galactosyrin is a potent β-galactosidase inhibitor.

Fig. S8. Homology and structural modelling predict GsnB function.

Fig. S9. Derivatised products and fragments detected by GC-MS.

Fig. S10. Detection of intermediates in galactosyrin biosynthesis *in vitro*.

Fig. S11. GsnA catalyses galactosyrin biosynthesis from 1ADR.

Fig. S12. GsnA catalyses the production of galactosyrin using 1ADR as a substrate and NAD^+ and Co^{2+} as cofactors.

Fig. S13. Chemical synthesis of galactosyrin.

Fig. S14. NMR spectra confirm the structure of the hydrate form of galactosyrin.

Fig. S15. Galactosyrin production causes accumulation of galactosylglycerol and trehalose in the apoplast independent of BGAL1.

Fig. S16. Galactosyrin inhibits not only BGAL1 but also other β-galactosidases in *N. benthamiana.*

Fig. S17. Galactosyrin inhibits different glycosidases.

Table S1. Tn5 insertion sites in galactosyrin-deficient mutants

Table S2. Metabolome of WT/Δgsn infection

Table S3. Bacterial strains used in this study

Table S4. Plasmids used in this study

Table S5. Primers used in this study

Table S6. Cryo-EM data collection, refinement and validation statistics

bioRxiv preprint doi: https://doi.org/10.1101/2025.02.13.638044; this version posted February 14, 2025. The copyright holder for this preprint (which was not certified by peer review) is the author/funder, who has granted bioRxiv a license to display the preprint in perpetuity. It is made available under aCC-BY 4.0 International license.

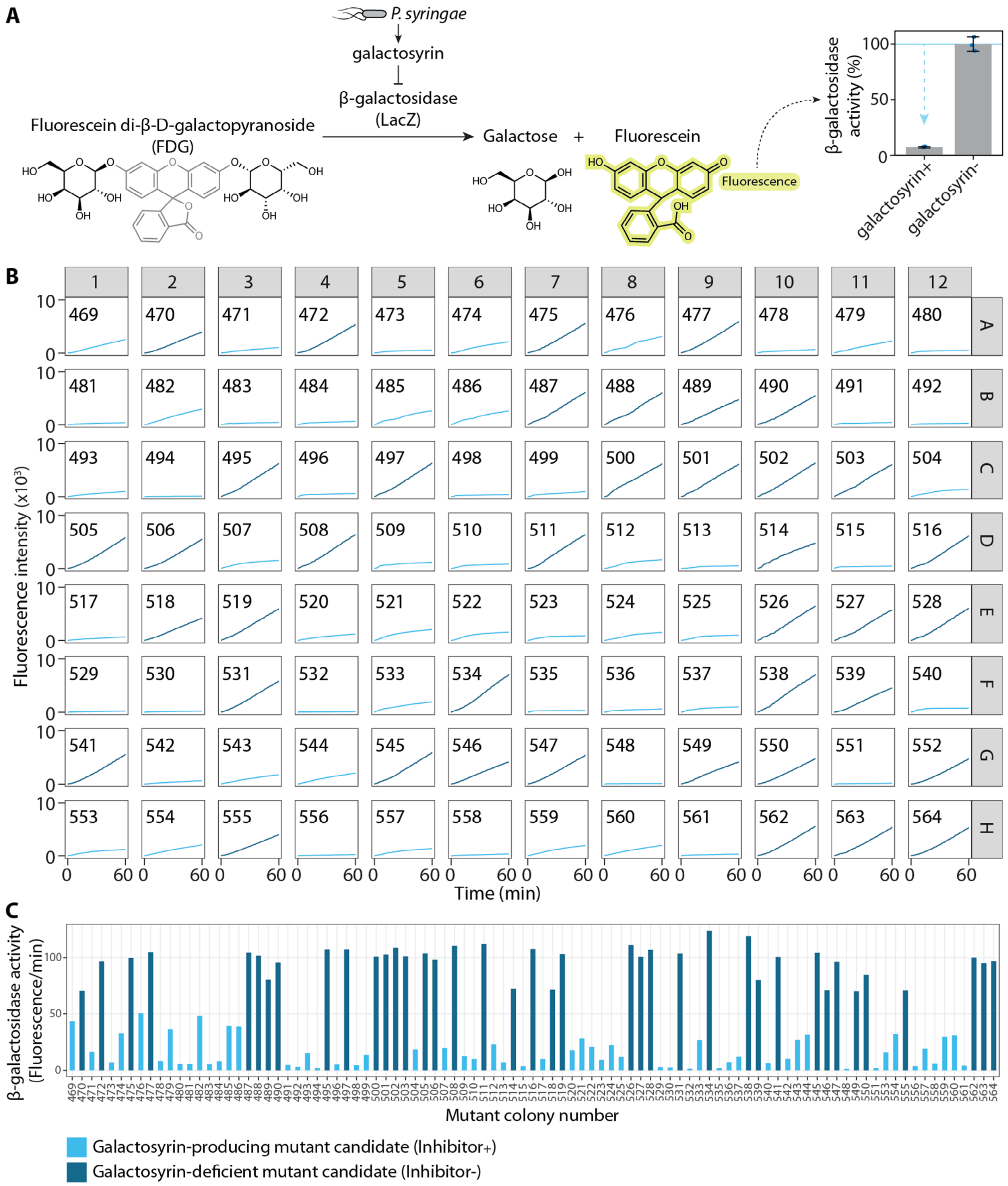

Fig. S1. Validation screen for candidate galactosyrin mutants.

(A) The enzymatic assay to monitor β-galactosidase inhibitor production. The cleavage of FDG substrate by β-galactosidase (LacZ) produces a fluorescent product fluorescein. The rate of fluorescence increase over time can be calculated as a measure of enzyme activity. The addition of galactosyrin produced by the bacteria inhibits LacZ activity. **(B)** Candidate galactosyrin mutants selected from plates were numbered and grown in virulence-inducing MG medium in each well of a 96-well plate overnight. The supernatants were then tested for the inhibitor in an enzymatic assay with FDG substrate and LacZ enzyme. Fluorescence

bioRxiv preprint doi: https://doi.org/10.1101/2025.02.13.638044; this version posted February 14, 2025. The copyright holder for this preprint (which was not certified by peer review) is the author/funder, who has granted bioRxiv a license to display the preprint in perpetuity. It is made available under aCC-BY 4.0 International license.

signal from cleavage product of FDG by LacZ was measured over time to monitor enzyme activity and inhibition. **(C)** β-galactosidase activity was calculated for each mutant and was classified into 2 groups: galactosyrin-producing mutant (Inhibitor+, light blue), causing low enzyme activity in the assay, and galactosyrin-deficient mutant (Inhibitor-, dark blue), causing high enzyme activity in the assay. The validated Inhibitor- mutants were selected for sequencing and mapping of inhibitor-related genes.

bioRxiv preprint doi: https://doi.org/10.1101/2025.02.13.638044; this version posted February 14, 2025. The copyright holder for this preprint (which was not certified by peer review) is the author/funder, who has granted bioRxiv a license to display the preprint in perpetuity. It is made available under aCC-BY 4.0 International license.

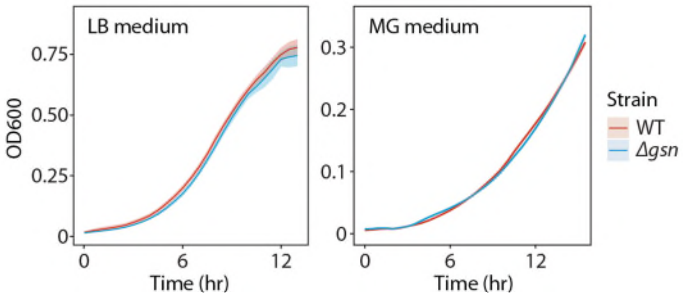

Fig. S2. Deletion of the *gsn* cluster does not affect bacterial growth *in vitro*.

Bacterial strains (wild-type (WT) or *gsn* cluster knockout (*Δgsn*)) were inoculated in LB medium or virulence-inducing MG medium in a 96-well plate and grown at 28 °C. Optical density at 600 nm (OD600) was measured over time to monitor bacterial growth. Mean and standard deviation from 3 replicates are plotted.

bioRxiv preprint doi: https://doi.org/10.1101/2025.02.13.638044; this version posted February 14, 2025. The copyright holder for this preprint (which was not certified by peer review) is the author/funder, who has granted bioRxiv a license to display the preprint in perpetuity. It is made available under aCC-BY 4.0 International license.

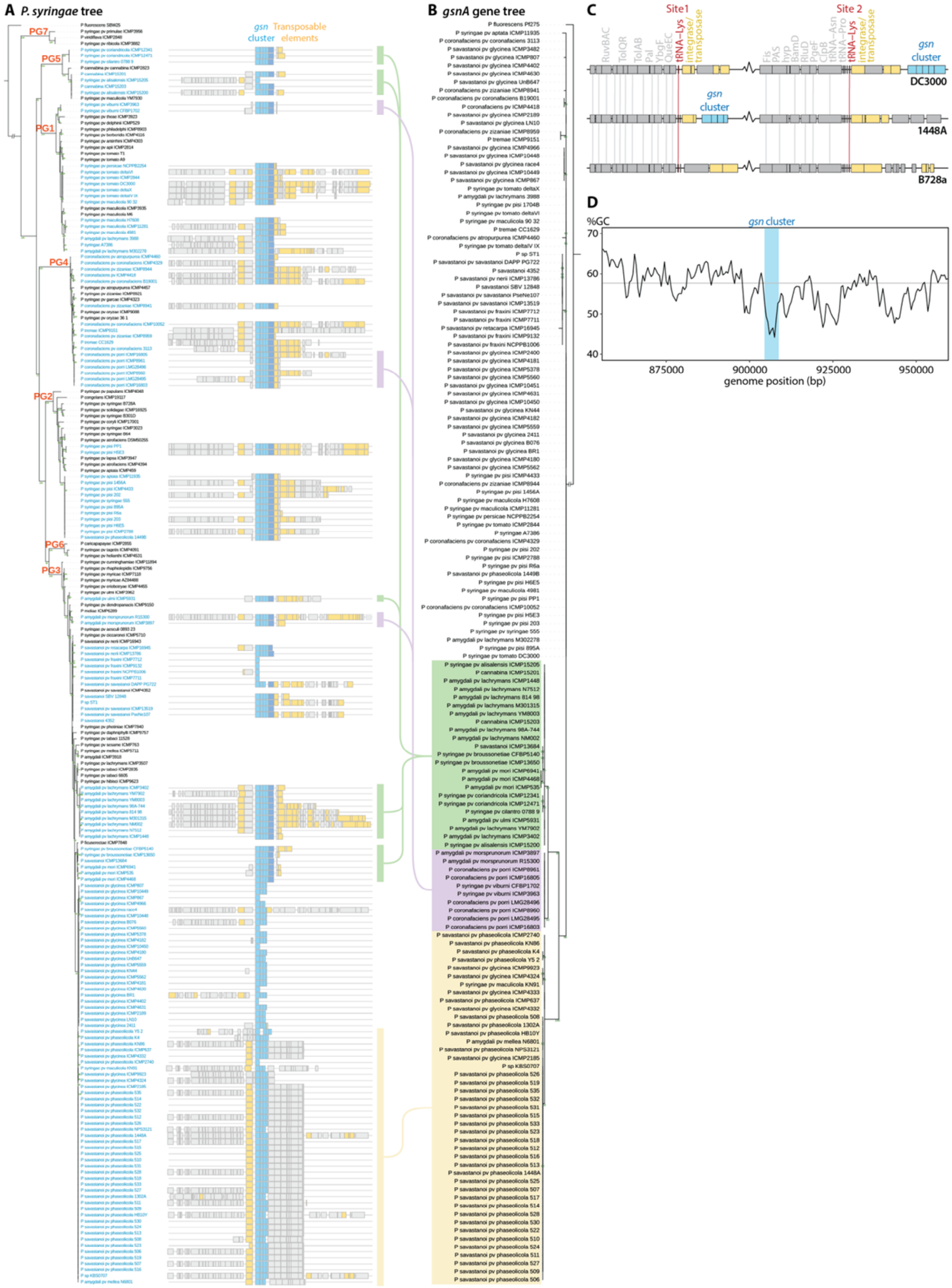

Fig. S3. The distribution of the *gsn* cluster among *P. syringae* strains is indicative of horizontal gene transfer.

bioRxiv preprint doi: https://doi.org/10.1101/2025.02.13.638044; this version posted February 14, 2025. The copyright holder for this preprint (which was not certified by peer review) is the author/funder, who has granted bioRxiv a license to display the preprint in perpetuity. It is made available under aCC-BY 4.0 International license.

(A) Phylogenetic tree of *P. syringae* strains. Maximum likelihood phylogenetic tree was constructed using concatenated coding DNA sequences of 4 conserved genes (*rpoD*, *gyrB*, *gltA* and *gapA*). Phylogroups (PG) are labelled with orange texts on each clade. *P. fluorescens* SBW25 is included as an outgroup. Strains with the *gsn* cluster are highlighted in blue. Genomic regions (20 kbp) around the *gsn* cluster are displayed on the right of each strain with annotated genes displayed as rectangles, the *gsn* cluster highlighted in blue and transposable elements highlighted in yellow. Many genomes are not completely assembled so truncated genomic regions are commonly observed. **(B)** Phylogenetic tree of *gsnA* genes. Maximum likelihood phylogenetic tree was constructed using coding DNA sequences of *gsnA* homologs identified from BLAST against genomes of *P. syringae* strains. A homolog from *P. fluorescens* Pf275 is included as an outgroup. Selected clades of closely related *gsnA* sequences are highlighted in the same colour (green, purple or orange) with lines connecting to the corresponding strains in the *P. syringae* tree, showing phylogenetic incongruence indicative of horizontal gene transfer. (B, C) Branch support values are calculated from ultrafast bootstrap with 1,000 replications. Branches with support values of less than 80 are collapsed. Branch lengths are drawn to scale representing the number of substitutions per site. **(C)** *gsn* clusters are found downstream of one of the two tRNA-lysine sites in the genome. Genomic regions surrounding the two tRNA-lysine sites (red) in the genomes of three *P. syringae* reference strains. In the strains with the *gsn* cluster (blue) (DC3000 and 1448A), the *gsn* clusters are found associated with transposable elements (yellow) downstream of the $tRNA^{lys}$ site, which are usual targets for integration. The *gsn* cluster is absent from the B728a strain. **(D)** *gsn* cluster has a lower GC content than average. Genome sequence of *P. syringae* pv. *tomato* DC3000 was analysed for percent GC content in 500 bp sliding windows across a 100 kbp region surrounding the *gsn* cluster (blue). Grey line indicates the overall GC content of the genome. A GC content different from the genome is suggestive of horizontal gene transfer origin.

bioRxiv preprint doi: https://doi.org/10.1101/2025.02.13.638044; this version posted February 14, 2025. The copyright holder for this preprint (which was not certified by peer review) is the author/funder, who has granted bioRxiv a license to display the preprint in perpetuity. It is made available under aCC-BY 4.0 International license.

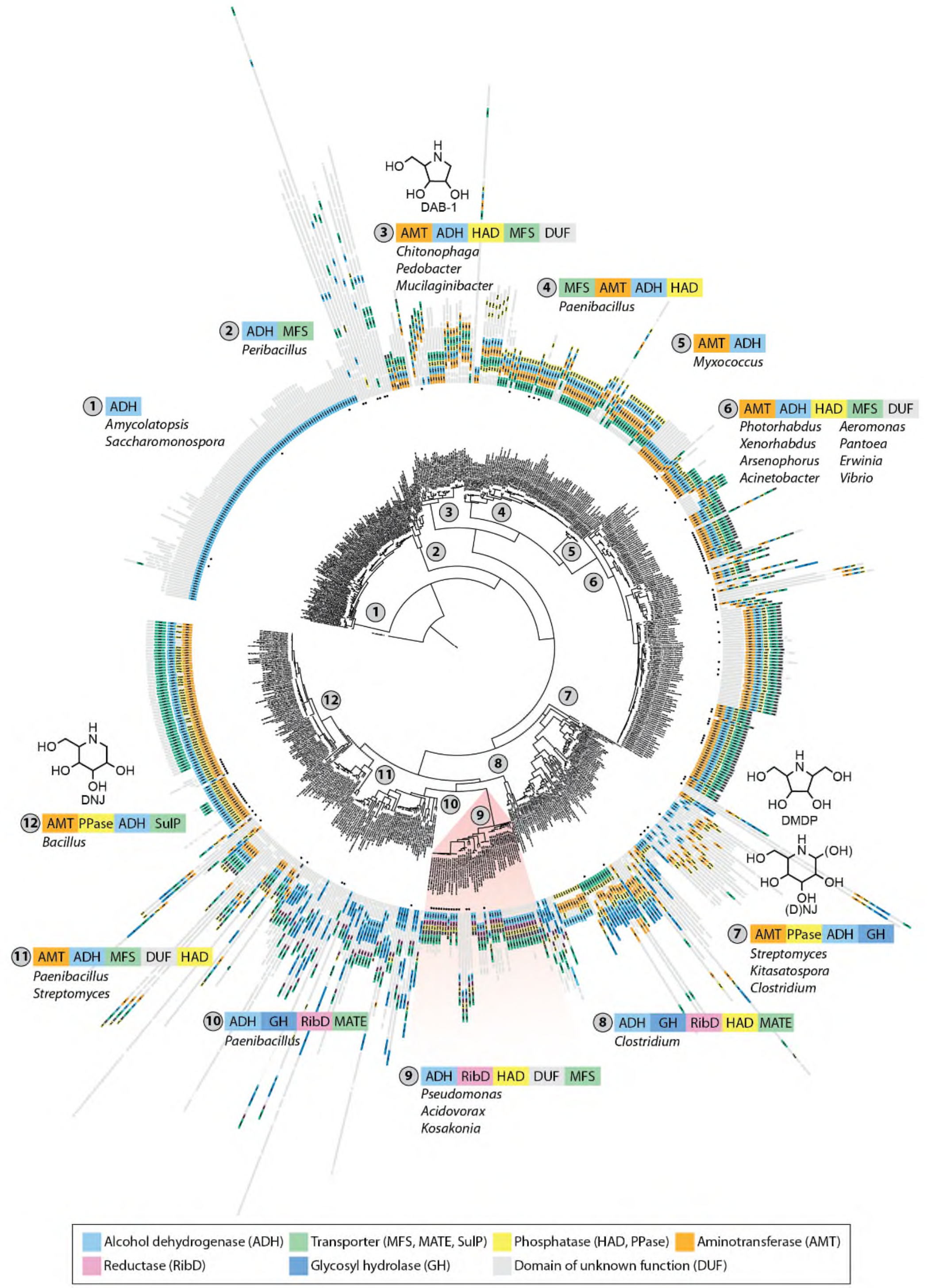

Fig. S4. *gsnA* homologs are part of different putative iminosugar biosynthesis gene clusters. Maximum likelihood phylogenetic tree was constructed using amino acid sequences of *gsnA* homologs [alcohol dehydrogenase (ADH)] identified by PSI-BLAST on Refseq database. Each homolog is labelled with its protein ID and the names of the species in which it is found. For brevity, a maximum of 5 homologs identified from each species are included. Branch support values are calculated from ultrafast bootstrap

bioRxiv preprint doi: https://doi.org/10.1101/2025.02.13.638044; this version posted February 14, 2025. The copyright holder for this preprint (which was not certified by peer review) is the author/funder, who has granted bioRxiv a license to display the preprint in perpetuity. It is made available under aCC-BY 4.0 International license.

with 1,000 replications. Branches with support values of less than 80 are collapsed. Branch lengths are drawn to scale representing the number of substitutions per site. Threonine dehydrogenase from *E. coli* is included as an outgroup. Genomic regions of putative gene clusters are displayed next to each entry with annotated genes displayed as rectangles and selected commonly occurring genes and families are highlighted in different colours. Solid black dots indicate gene cluster association with transposable elements. Homologs and clusters are classified into 12 distinct clades (labelled with numbers in a circle) based on tree topology. Representative gene cluster composition and bacterial genus are shown for each clade. Iminosugars reported to be produced by members of the clades are shown (*7–9*). Clade 9 containing the *P. syringae gsn* cluster is highlighted in red.

bioRxiv preprint doi: https://doi.org/10.1101/2025.02.13.638044; this version posted February 14, 2025. The copyright holder for this preprint (which was not certified by peer review) is the author/funder, who has granted bioRxiv a license to display the preprint in perpetuity. It is made available under aCC-BY 4.0 International license.

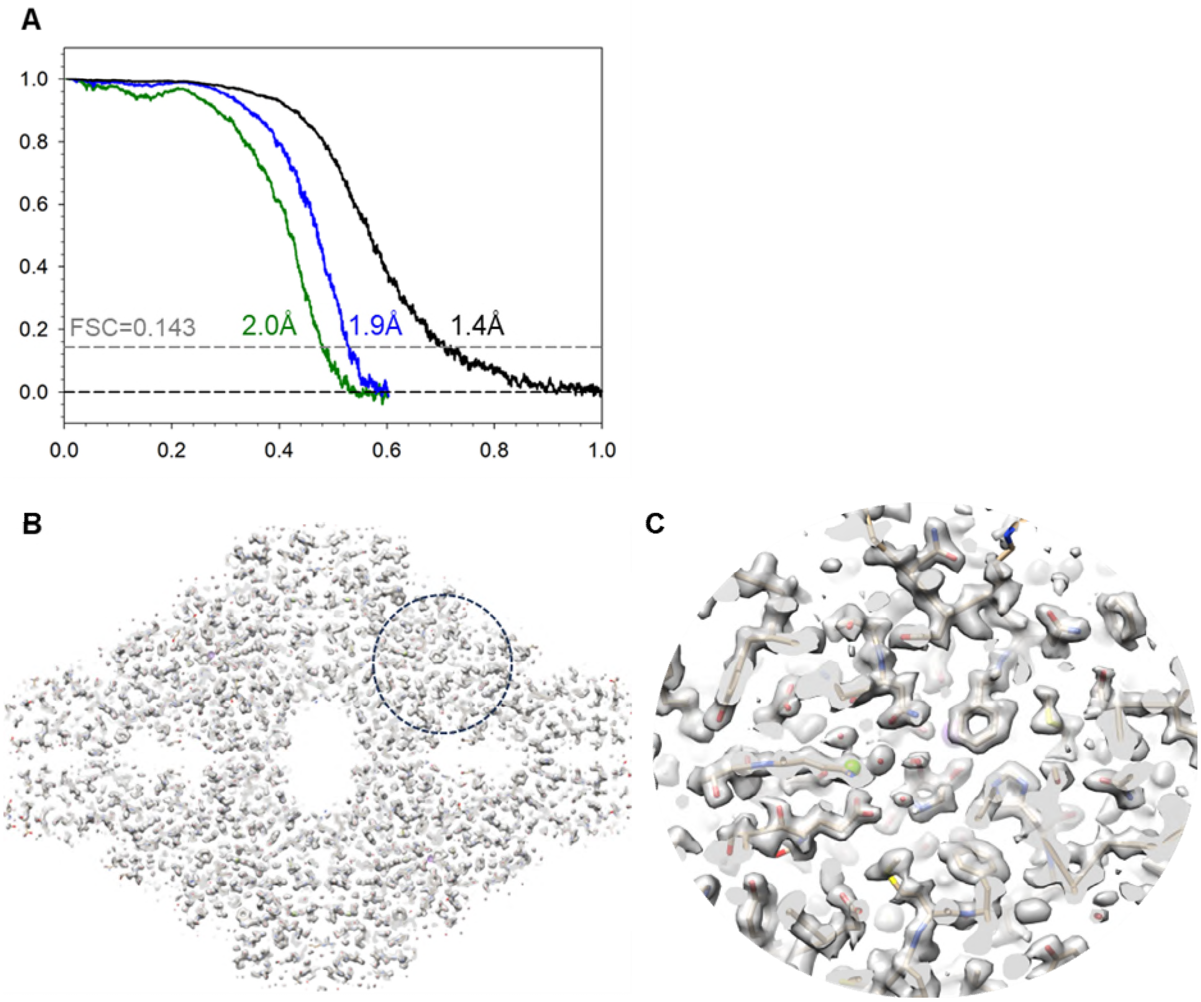

Fig. S5. Cryo-EM structure determination of β-galactosidase (LacZ) bound with galactosyrin.

(A) Fourier Shell Correlation (FSC) between the two independently refined half-maps for LacZ in complex with the native inhibitor (WT, blue), the negative control Δ*gsn* (green), and with the synthetic galactosyrin (black). **(B)** Cryo-EM map of LacZ bound with the synthetic galactosyrin, shown with a central slice for clarity. **(C)** A zoom-in view of the LacZ active site circled in (B). Atomic details of the density map are clearly displayed.

bioRxiv preprint doi: https://doi.org/10.1101/2025.02.13.638044; this version posted February 14, 2025. The copyright holder for this preprint (which was not certified by peer review) is the author/funder, who has granted bioRxiv a license to display the preprint in perpetuity. It is made available under aCC-BY 4.0 International license.

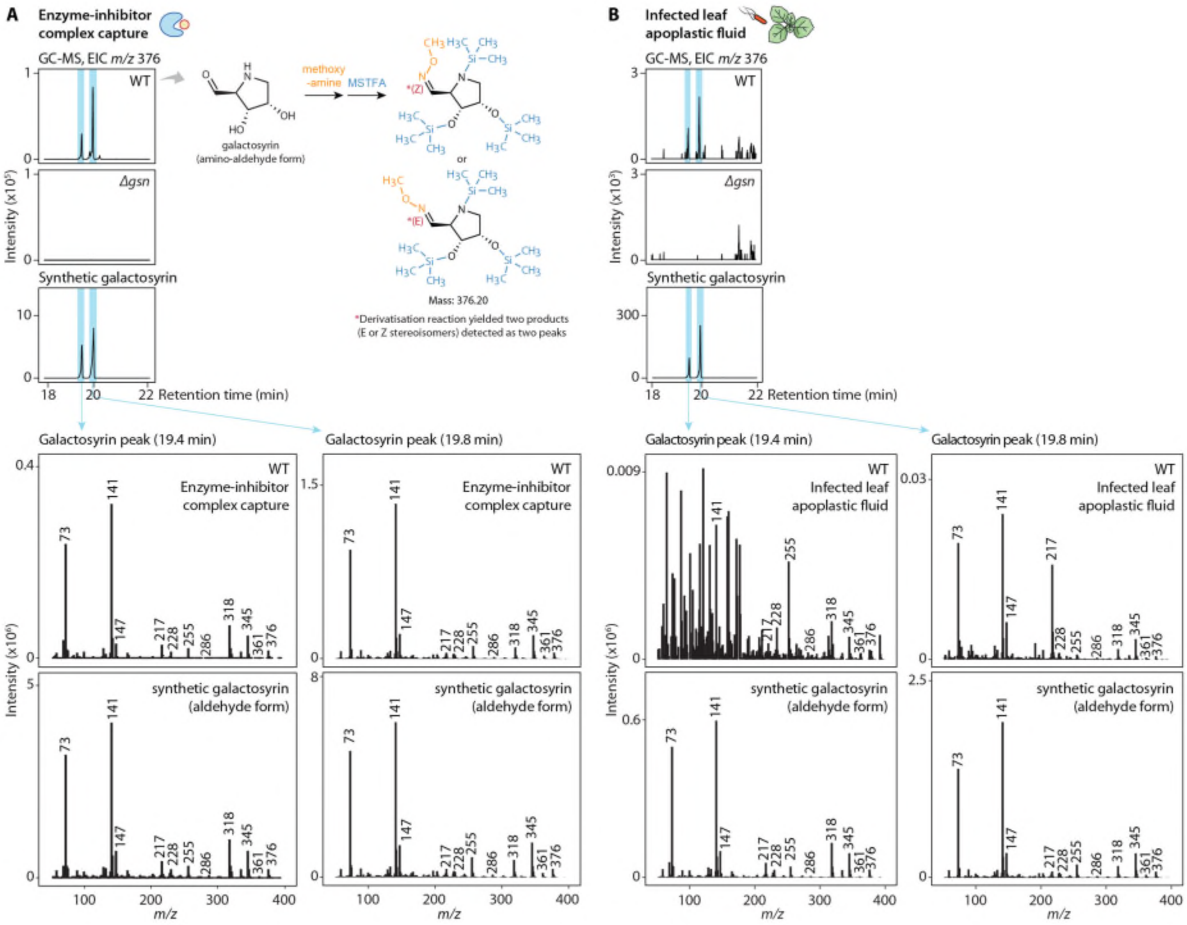

Fig. S6. Galactosyrin was detected in enzyme-inhibitor complex and apoplast of infected plants. (Top) Extracted ion chromatogram (EIC) of *m/z* 376 from GC-MS analysis of synthetic galactosyrin and soluble metabolite extracts from **(A)** enzyme-inhibitor complex capture from cultures of galactosyrin-producing (WT) or galactosyrin-deficient (*Δgsn*) strains (**Fig. 2**) and **(B)** apoplastic fluid from *N. benthamiana* leaves infected with WT or *Δgsn* strains. Samples were analysed by GC-MS after chemical modification with methoxyamine, which modifies the aldehyde group, and N-Methyl-N-(trimethylsilyl)trifluoroacetamide (MSTFA), which modifies hydroxyl and amine groups, to enable carbohydrate analysis. Since methoxyamine modification yields two stereoisomers, E and Z (*34*), galactosyrin was detected as two peaks. Blue stripes highlight galactosyrin peaks. (Bottom) Mass spectra of the detected galactosyrin peaks showing identical expected masses. Compound structures, derivatisation and mass annotations are shown in **Fig. S9**. The galactosyrin peak (19.4 min) detected in the infected apoplastic fluid sample had a relatively low intensity and co-eluted with an unknown metabolite form the plant apoplast so high background noise was observed in the mass spectrum.

bioRxiv preprint doi: https://doi.org/10.1101/2025.02.13.638044; this version posted February 14, 2025. The copyright holder for this preprint (which was not certified by peer review) is the author/funder, who has granted bioRxiv a license to display the preprint in perpetuity. It is made available under aCC-BY 4.0 International license.

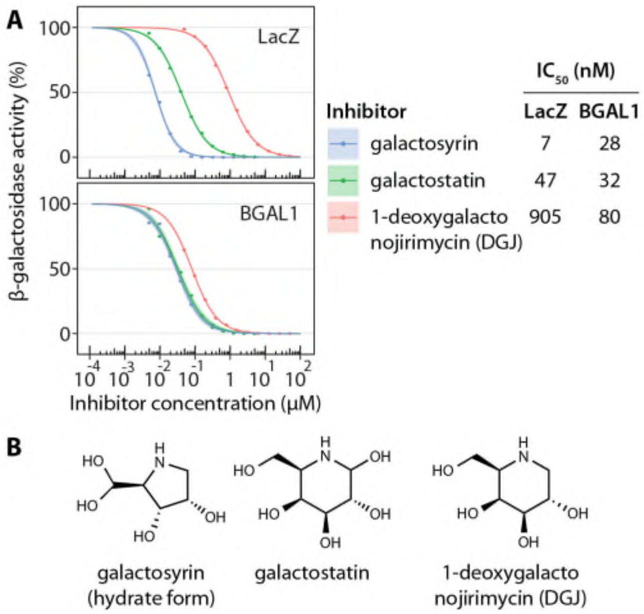

Fig. S7. Galactosyrin is a potent β-galactosidase inhibitor.

(A) Dose-response curves of galactosyrin, galactostatin and 1-deoxygalactonojirimycin (DGJ) inhibition of LacZ or BGAL1 with FDG substrate. β-galactosidase activity is expressed as a percentage of the activity relative to no inhibitor control. A four-parameter logistic model was fitted and half maximal inhibitory concentration (IC50) is shown for each enzyme and inhibitor combination. (B) Structures of inhibitors tested.

bioRxiv preprint doi: https://doi.org/10.1101/2025.02.13.638044; this version posted February 14, 2025. The copyright holder for this preprint (which was not certified by peer review) is the author/funder, who has granted bioRxiv a license to display the preprint in perpetuity. It is made available under aCC-BY 4.0 International license.

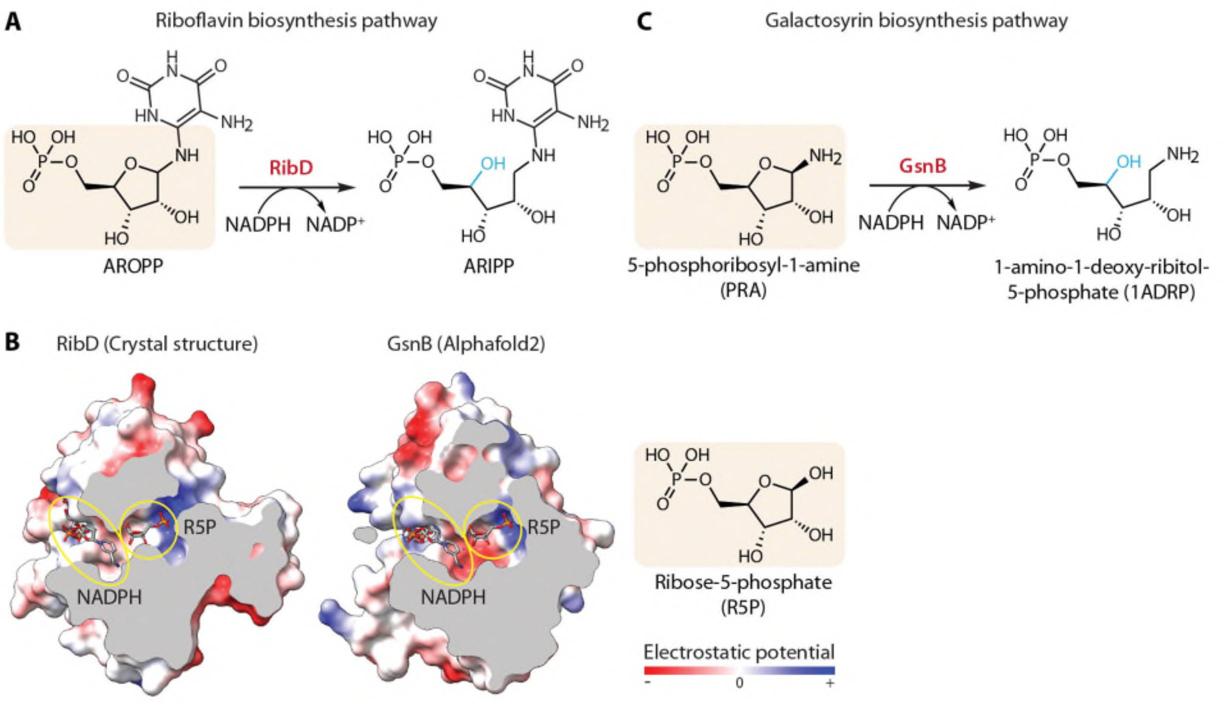

Fig. S8. Homology and structural modelling predict GsnB function.

(A) RibD, a close domain family member of GsnB, functions in the riboflavin biosynthesis pathway by catalysing the reduction of 5-amino-6-ribosylamino-2,4(1H,3H)-pyrimidinedione 5′-phosphate (AROPP) to 5-amino-6-ribitylamino-2,4(1H,3H)-pyrimidinedione 5′-phosphate (ARIPP). **(B)** Comparison of crystal structures of RibD from *E. coli* in complex with the ribose-5-phosphate (R5P) substrate analog (PDB: 2OBC) or NADPH cofactor (PDB: 2O7P) and Alphafold2 predicted structure of GsnB (RMSD (angstrom) of 1.07 between 125 pruned atom pairs, 7.79 across all 187 pairs compared to 2OBC and 1.06 between 125 pruned atom pairs, 4.53 across all 183 pairs compared to 2O7P). The enzyme structure cross section shows the conserved active site pockets (yellow circles) with the overlay of the structure of the substrate analog (R5P) and cofactor (NADPH). Protein structures are coloured based on electrostatic potential of amino acid residues (red: negative - blue: positive) to show the conserved properties at the sites for substrate and cofactor binding. **(C)** GsnB was predicted then proven to function in galactosyrin biosynthesis pathway by catalysing the reduction of 5-phosphoribosyl-1-amine (PRA) to 1-amino-1-deoxy-ribitol-5-phosphate (1ADRP). Chemical change in the product compared to the substrate is highlighted in blue. Similar structures of the substrates and analogs are highlighted in rounded rectangle.

bioRxiv preprint doi: https://doi.org/10.1101/2025.02.13.638044; this version posted February 14, 2025. The copyright holder for this preprint (which was not certified by peer review) is the author/funder, who has granted bioRxiv a license to display the preprint in perpetuity. It is made available under aCC-BY 4.0 International license.

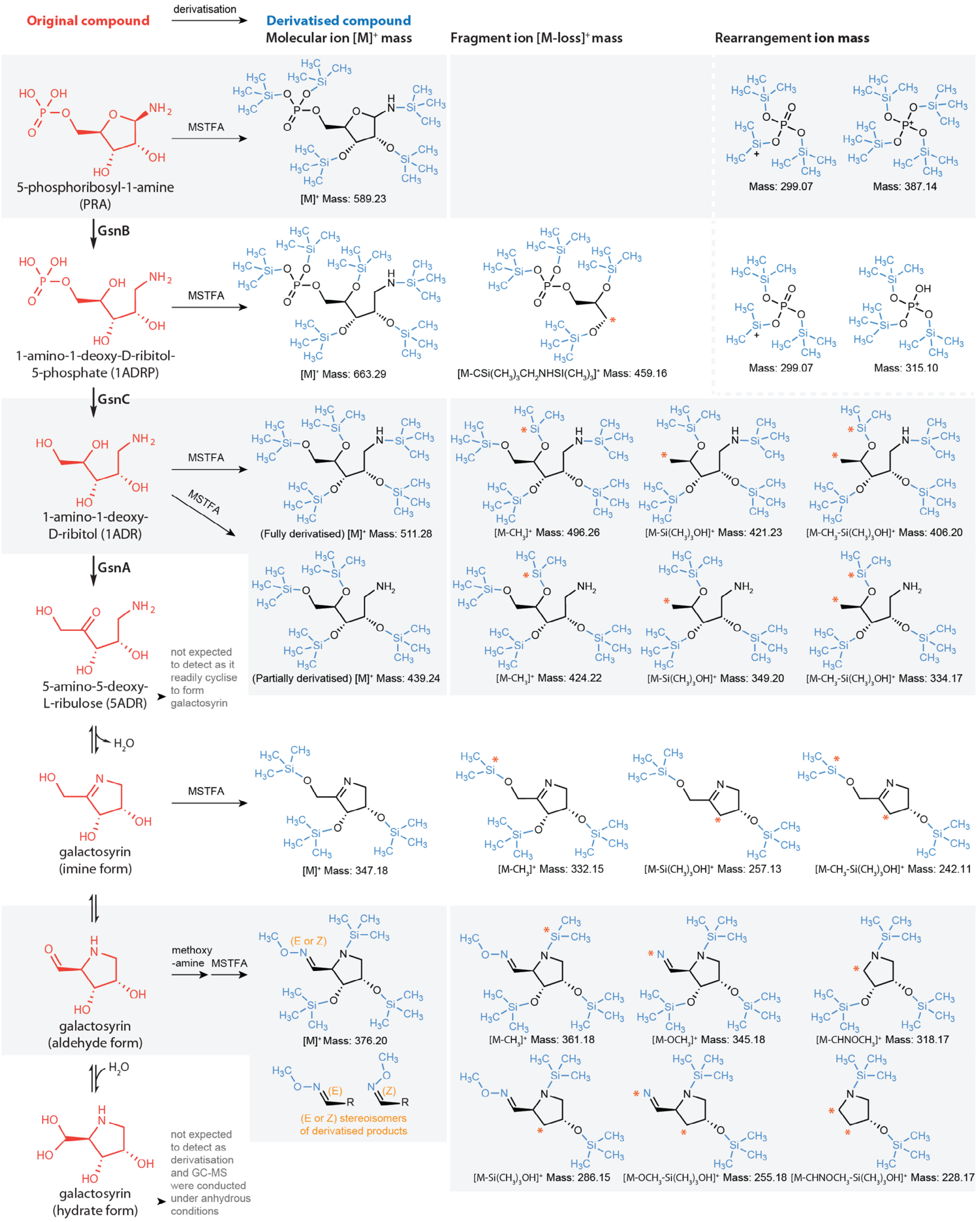

Fig. S9. Derivatised products and fragments detected by GC-MS.

(Left) Compounds in the galactosyrin biosynthesis pathway with structures shown in red and chemical derivatisation for GC-MS analysis indicated on arrows. MSTFA (N-Methyl-N-(trimethylsilyl)trifluoroacetamide) derivatises hydroxyl and amine groups, while methoxyamine derivatises aldehyde groups yielding two products with stereoisomers E or Z, detected as two peaks for the aldehyde

bioRxiv preprint doi: https://doi.org/10.1101/2025.02.13.638044; this version posted February 14, 2025. The copyright holder for this preprint (which was not certified by peer review) is the author/funder, who has granted bioRxiv a license to display the preprint in perpetuity. It is made available under aCC-BY 4.0 International license.

form of galactosyrin. Compounds not expected to be detected in GC-MS are indicated with a description in grey. (Right) Derivatised compounds with the derivatisation on the original structure shown in blue. The mass expected in GC-MS detection are indicated below each structure. Molecular ion shows the intact derivatised compounds. Fragment ion shows expected fragments from the fragmentation of molecular ion caused by electron ionisation (EI) during GC-MS. Orange asterisks indicate the group lost by fragmentation. Rearrangement ion shows expected products from rearrangement of the molecular and fragment ions during GC-MS. Data interpretation was facilitated by general principles of derivatisation, fragmentation and rearrangement in GC-MS (*35*, *36*).

bioRxiv preprint doi: https://doi.org/10.1101/2025.02.13.638044; this version posted February 14, 2025. The copyright holder for this preprint (which was not certified by peer review) is the author/funder, who has granted bioRxiv a license to display the preprint in perpetuity. It is made available under aCC-BY 4.0 International license.

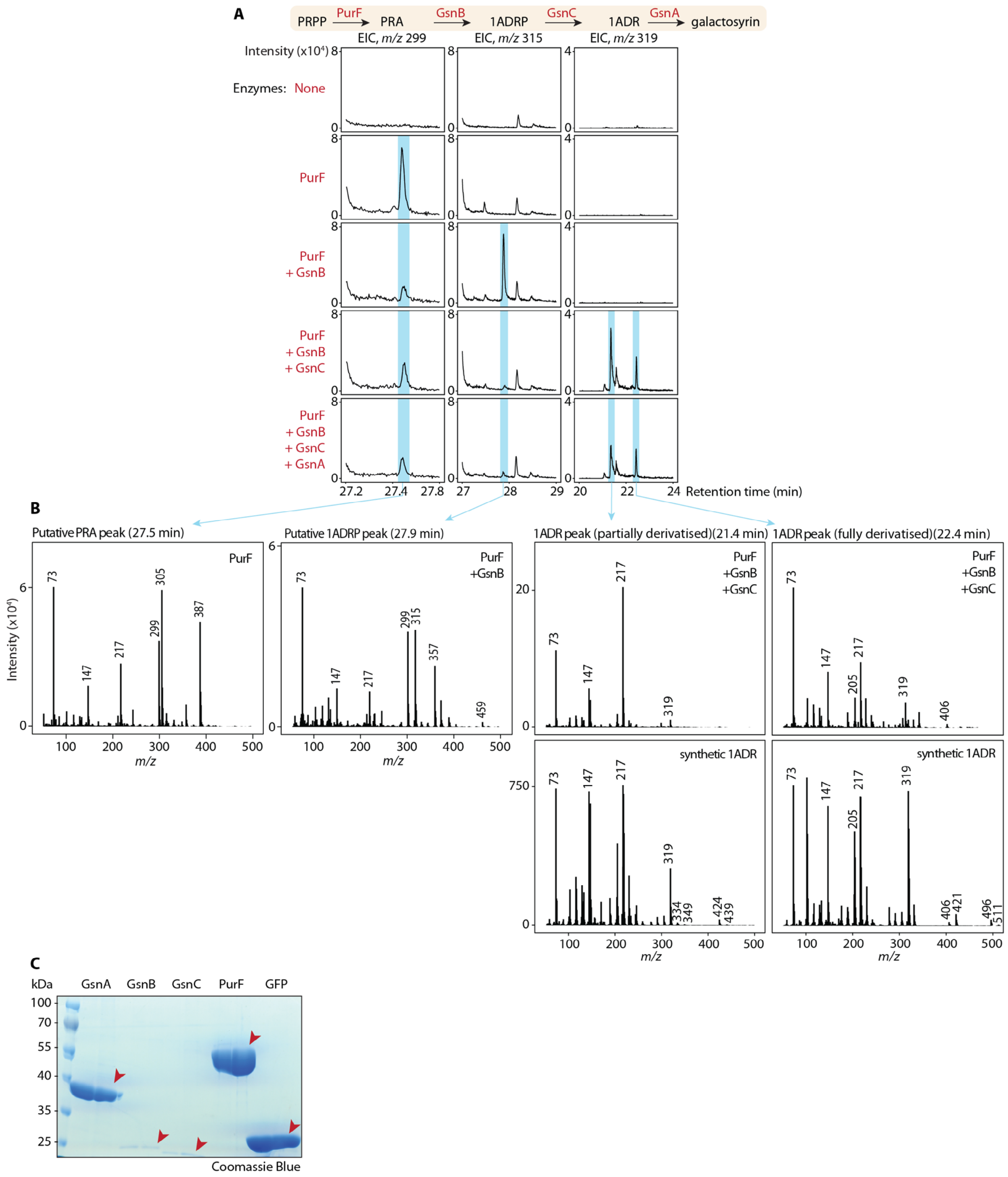

Fig. S10. Detection of intermediates in galactosyrin biosynthesis *in vitro*.

(A) Summary diagram of the biosynthesis pathway is shown at the top. Extracted ion chromatograms (EIC) of mass signature corresponding to each intermediate (PRA, 1ADRP and 1ADR) are shown in each column. For each row, each mixture of enzymes (listed in red on the left) were mixed with the starting precursor (PRPP) and cofactors to reconstruct the biosynthesis pathway, then soluble metabolite fractions were analysed by GC-MS. Blue stripes highlight peaks of the detected intermediates, accumulating in samples

bioRxiv preprint doi: https://doi.org/10.1101/2025.02.13.638044; this version posted February 14, 2025. The copyright holder for this preprint (which was not certified by peer review) is the author/funder, who has granted bioRxiv a license to display the preprint in perpetuity. It is made available under aCC-BY 4.0 International license.

containing enzymes that produce them and decreasing in samples containing enzymes that use them. **(B)** Mass spectra of the detected peaks. For 1ADR, the matching spectra of a synthetic standard were also shown underneath. Compound structures, derivatisation and mass annotations are shown in **Fig. S9**. **(C)** Purified enzymes used in the experiment. Proteins were separated on SDS-PAGE and stained with Coomassie blue. Orange arrows indicate the expected protein band.

bioRxiv preprint doi: https://doi.org/10.1101/2025.02.13.638044; this version posted February 14, 2025. The copyright holder for this preprint (which was not certified by peer review) is the author/funder, who has granted bioRxiv a license to display the preprint in perpetuity. It is made available under aCC-BY 4.0 International license.

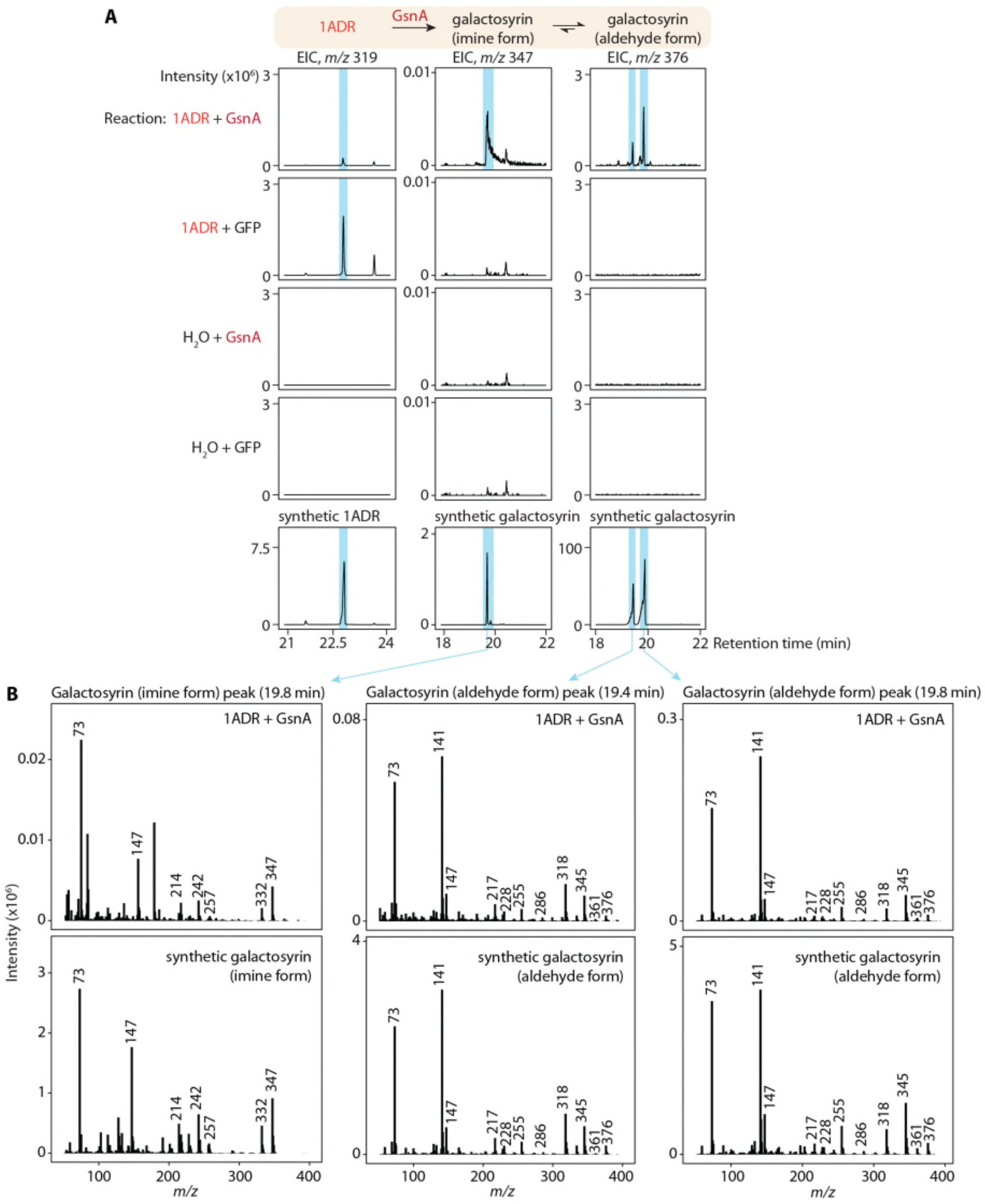

Fig. S11. GsnA catalyses galactosyrin biosynthesis from 1ADR.

(A) Summary diagram of the biosynthesis step is shown at the top. 1ADR is converted by GsnA into the galactosyrin imine form which can spontaneously convert into the aldehyde form. Extracted ion chromatograms (EIC) of mass signature corresponding to each compound (1ADR, galactosyrin imine and aldehyde form) are shown in each column. For each row, each mixture of enzyme and substrate (listed in

bioRxiv preprint doi: https://doi.org/10.1101/2025.02.13.638044; this version posted February 14, 2025. The copyright holder for this preprint (which was not certified by peer review) is the author/funder, who has granted bioRxiv a license to display the preprint in perpetuity. It is made available under aCC-BY 4.0 International license.

red and orange on the left, GFP used as negative control) were mixed with the cofactors to reconstruct the biosynthesis, then soluble metabolite fractions were analysed by GC-MS. Blue stripes highlight peaks of the detected compounds, which are confirmed to match with synthetic standards shown in the bottom row. Galactosyrin (aldehyde form) was derivatized with methoxyamine, which yielded two stereoisomers detected as two peaks. **(B)** Mass spectra of the detected peaks with the matching spectra of synthetic standards shown underneath. Compound structures, derivatisation and mass annotations are shown in **Fig. S9**.

bioRxiv preprint doi: https://doi.org/10.1101/2025.02.13.638044; this version posted February 14, 2025. The copyright holder for this preprint (which was not certified by peer review) is the author/funder, who has granted bioRxiv a license to display the preprint in perpetuity. It is made available under aCC-BY 4.0 International license.

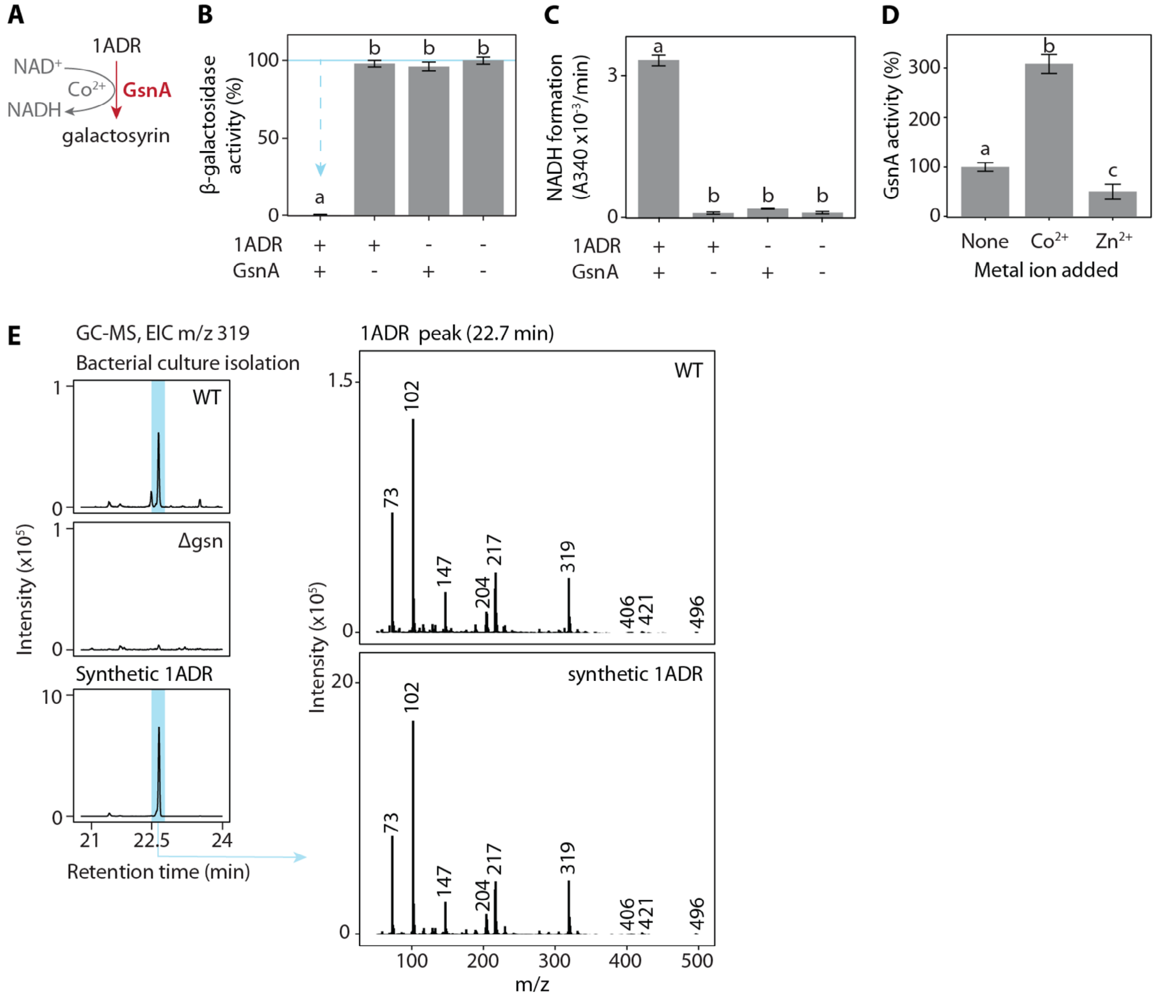

Fig. S12. GsnA catalyses the production of galactosyrin using 1ADR as a substrate and NAD^+ and Co^{2+} as cofactors.

(A) Summary of the GsnA catalysed reaction. **(B)** Galactosyrin is produced by GsnA using 1ADR substrate. The enzyme GsnA (+) or GFP (-) and substrate 1ADR (+) or water (-) were mixed with cofactors, then the mixture was tested for galactosyrin formation in an enzyme activity assay with FDG substrate and LacZ enzyme. β-galactosidase activity is expressed as a percentage of the activity relative to the mean of no enzyme/substrate control. Arrows indicate inhibition. **(C)** NADH is formed from the oxidation of 1ADR catalysed by GsnA. The same reactions as (B) were monitored for NADH formation by measuring absorbance at 340 nm (A340). **(D)** GsnA has increased activity with Co^{2+} as a cofactor. Purified GsnA was pre-treated with EDTA to remove endogenously bound metal then used in 1ADR and NAD^+ reaction mixture with or without Co^{2+} or Zn^{2+}. The activity of GsnA was monitored with the rate of NADH formation and expressed as a percentage relative to no metal added sample. (B, C, D) Mean and standard deviation from 3 replicates are plotted. Different letters indicate different groups with statistically significant difference ($P < 0.05$) using one-way ANOVA and post-hoc Tukey HSD test. **(E)** 1ADR was also detected

bioRxiv preprint doi: https://doi.org/10.1101/2025.02.13.638044; this version posted February 14, 2025. The copyright holder for this preprint (which was not certified by peer review) is the author/funder, who has granted bioRxiv a license to display the preprint in perpetuity. It is made available under aCC-BY 4.0 International license.

in bacterial culture. (Left) Extracted ion chromatogram (EIC) of *m/z* 319 from GC-MS analysis of cation-exchanged-isolated soluble metabolite extracts from cultures of galactosyrin-producing (WT) or galactosyrin-deficient (*Δgsn*) strains. Blue stripes highlight the peak of 1ADR matching with the synthetic standard. (Right) Mass spectra of the detected 1ADR peaks showing identical expected masses. Compound structures, derivatisation and mass annotations are shown in **Fig. S9**.

bioRxiv preprint doi: https://doi.org/10.1101/2025.02.13.638044; this version posted February 14, 2025. The copyright holder for this preprint (which was not certified by peer review) is the author/funder, who has granted bioRxiv a license to display the preprint in perpetuity. It is made available under aCC-BY 4.0 International license.

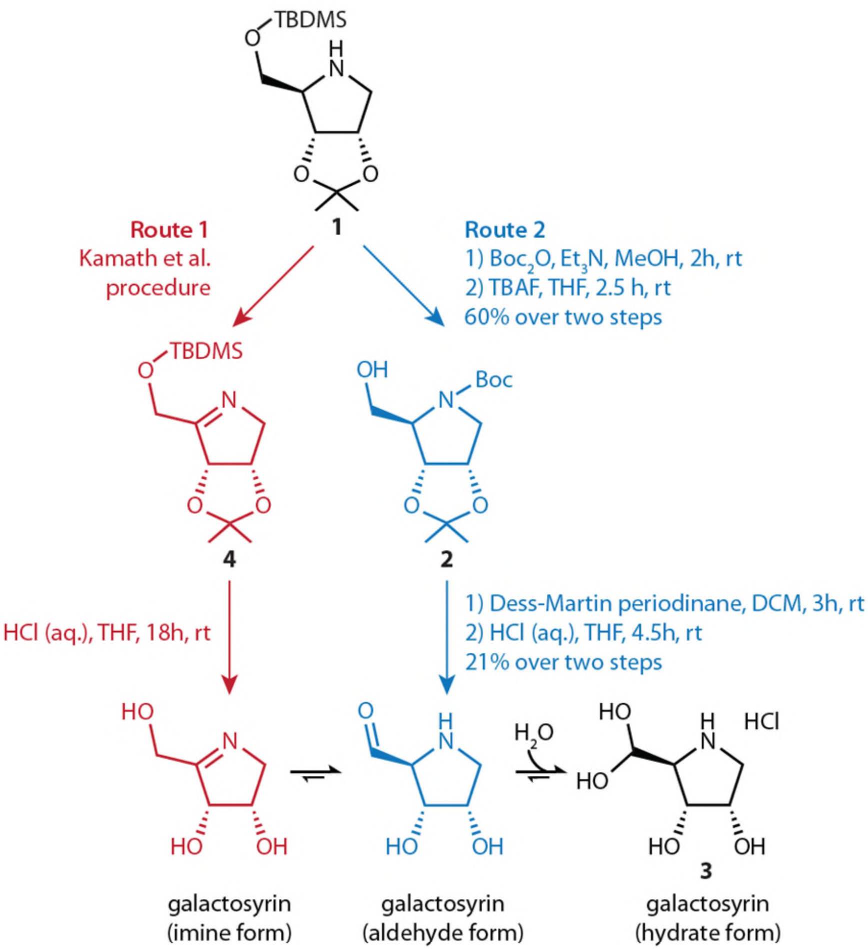

Fig. S13. Chemical synthesis of galactosyrin.

The hydrate form of galactosyrin can be obtained through two synthesis routes: route 1 via galactosyrin imine form using Kamath et al. procedure (*37*) and route 2 via galactosyrin aldehyde form

bioRxiv preprint doi: https://doi.org/10.1101/2025.02.13.638044; this version posted February 14, 2025. The copyright holder for this preprint (which was not certified by peer review) is the author/funder, who has granted bioRxiv a license to display the preprint in perpetuity. It is made available under aCC-BY 4.0 International license.

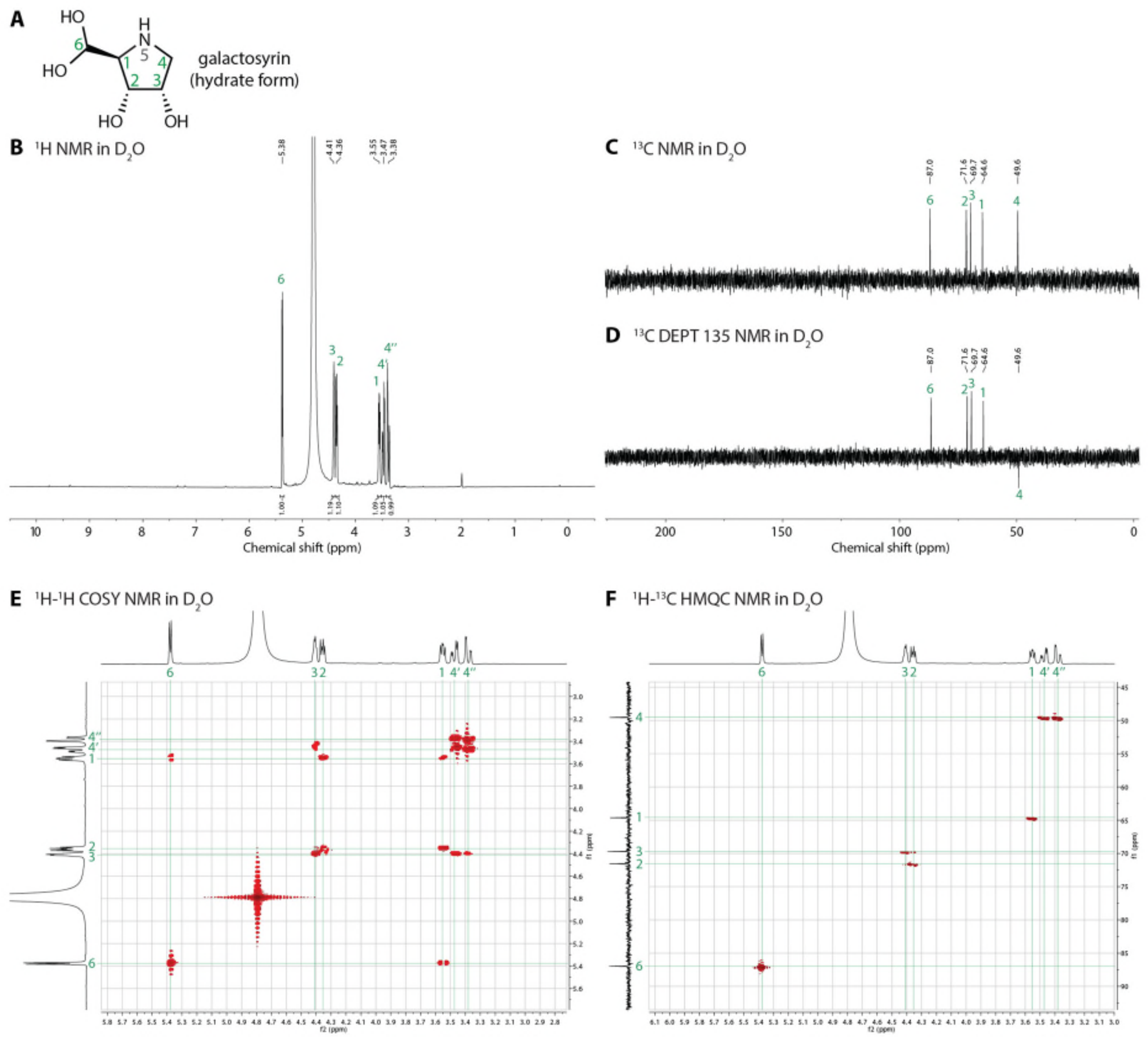

Fig. S14. NMR spectra confirm the structure of the hydrate form of galactosyrin.

(A) Structure of galactosyrin hydrate form with atom positions numbered (green) for assignment in NMR spectra. **(B)** ^{1}H NMR. **(C)** ^{13}C NMR. **(D)** ^{13}C distortionless enhancement by polarization transfer (DEPT) 135 NMR. **(E)** ^{1}H-^{1}H homonuclear correlated spectroscopy (COSY) NMR. **(F)** ^{1}H-^{13}C heteronuclear multiple quantum correlation (HMQC) NMR.

bioRxiv preprint doi: https://doi.org/10.1101/2025.02.13.638044; this version posted February 14, 2025. The copyright holder for this preprint (which was not certified by peer review) is the author/funder, who has granted bioRxiv a license to display the preprint in perpetuity. It is made available under aCC-BY 4.0 International license.

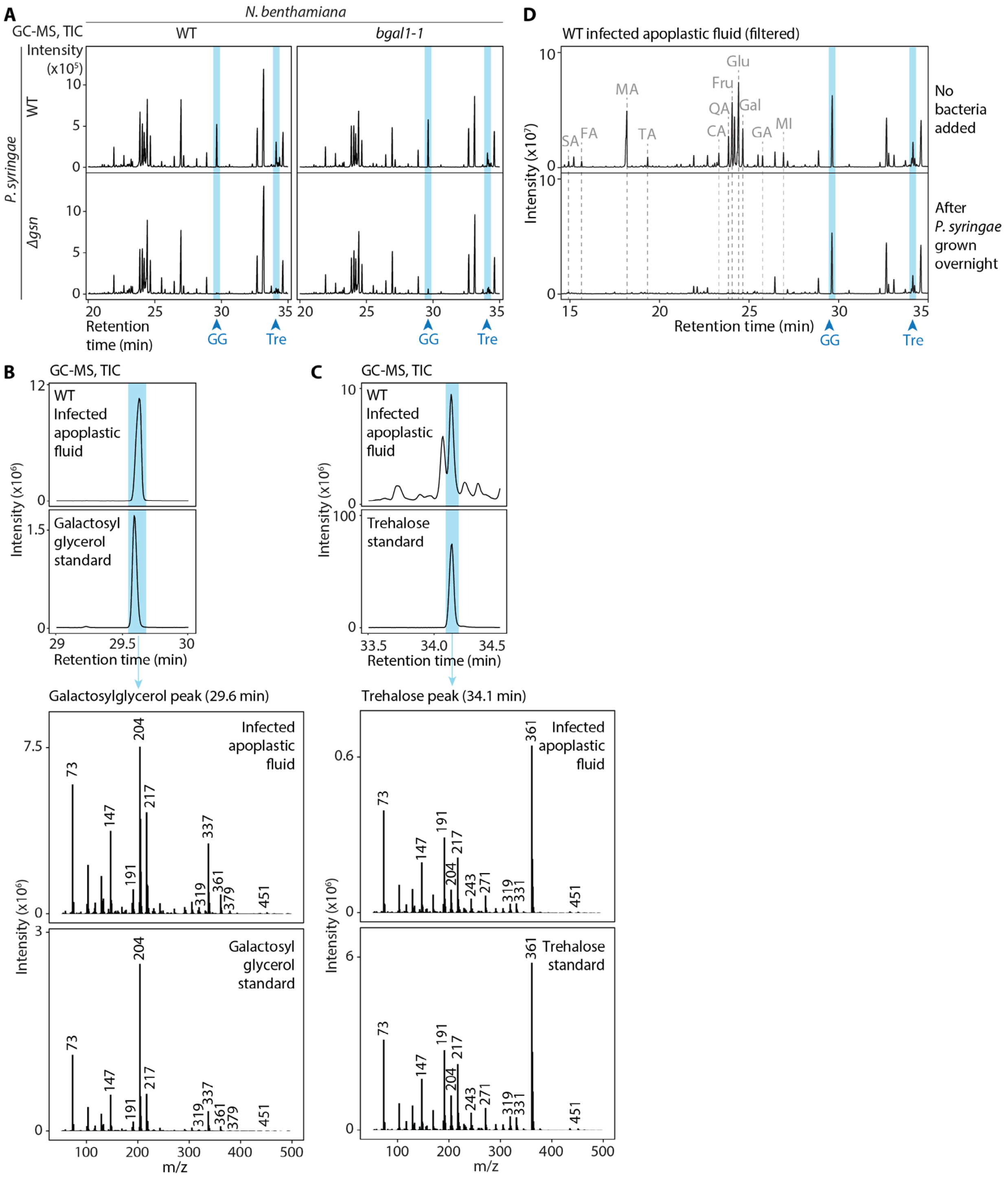

Fig. S15. Galactosyrin production causes accumulation of galactosylglycerol and trehalose in the apoplast independent of BGAL1.

(A) Total ion chromatograms (TIC) from GC-MS of soluble metabolites extracted from apoplastic fluids from *N. benthamiana* (wild-type (WT) or BGAL1 knockout mutant (*bgal1-1*) infected by galactosyrin-producing *P. syringae* (WT) or galactosyrin-deficient mutant (*Δgsn*). Blue stripes highlight peaks of

bioRxiv preprint doi: https://doi.org/10.1101/2025.02.13.638044; this version posted February 14, 2025. The copyright holder for this preprint (which was not certified by peer review) is the author/funder, who has granted bioRxiv a license to display the preprint in perpetuity. It is made available under aCC-BY 4.0 International license.

galactosylglycerol (GG) and trehalose (Tre). **(B)** Galactosylglycerol and **(C)** trehalose detection in the infected apoplastic fluid were confirmed by synthetic standards. (Top) Total ion chromatograms (TIC) from GC-MS of *P. syringae* (WT) infected apoplastic fluid. Blue stripes highlight peaks of the detected compounds, which are confirmed to match with synthetic standards shown underneath. (Bottom) Mass spectra of the detected peaks with the matching spectra of synthetic standards shown underneath. **(D)** Galactosylglycerol and trehalose are not preferred nutrient sources of *P. syringae*. Apoplastic fluid from *P. syringae* (WT)-infected *N. benthamiana* (WT) leaves was filter-sterilised then used as a culture medium for *P. syringae* WT with OD600 of 1 overnight at 28°C or incubated without the bacteria. Soluble metabolites were extracted from these samples, analysed with GC-MS and total ion chromatograms (TIC) are shown. Blue stripes highlight peaks of galactosylglycerol (GG) and trehalose (Tre), which were not depleted after *P. syringae* growth. Dashed lines highlight peaks of metabolites that were depleted, including sugars: fructose (Fru), glucose (Glu), galactose (Gal), organic acids: succinic (SA), fumaric (FA), malic (MA), threonic (TA), citric (CA), quinic (QA), gluconic (GA) acid and myo-inositol (MI).

bioRxiv preprint doi: https://doi.org/10.1101/2025.02.13.638044; this version posted February 14, 2025. The copyright holder for this preprint (which was not certified by peer review) is the author/funder, who has granted bioRxiv a license to display the preprint in perpetuity. It is made available under aCC-BY 4.0 International license.

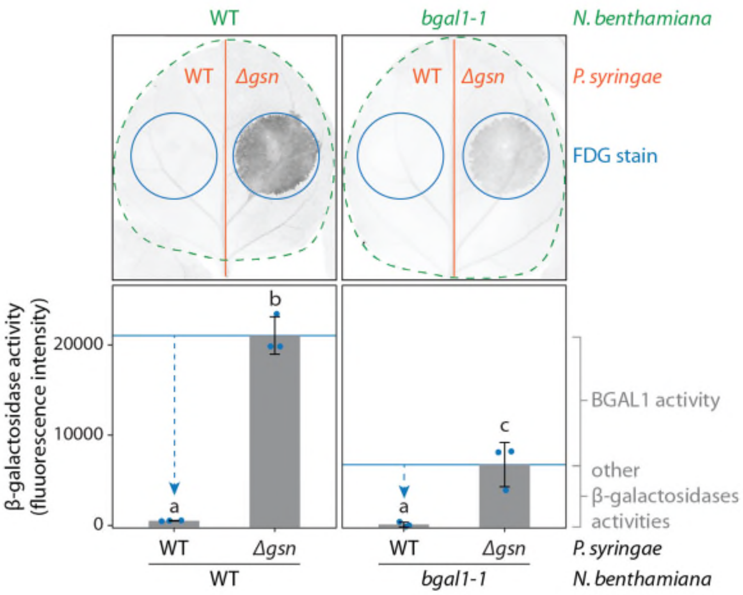

Fig. S16. Galactosyrin inhibits not only BGAL1 but also other β-galactosidases in *N. benthamiana.*

(Top) *N. benthamiana* leaves (WT or *bgal1-1* mutant lacking BGAL1 β-galactosidase) were infected with *P. syringae* (WT or the galactosyrin-deficient *Δgsn* mutant) infiltrated into each half of each leaf. At three days post infection, the fluorogenic substrate FDG was infiltrated as spots into the leaves and β-galactosidase activity was measured by imaging the fluorescence signal of FDG cleavage product. (Bottom) β-galactosidase activity was quantified using integrated density of fluorescence signal from each spot. Error bars represent standard deviation from 3 replicates. Different letters indicate different groups with statistically significant difference ($P < 0.01$) using one-way ANOVA and post-hoc Tukey HSD test. Arrows highlight inhibition. β-galactosidase activity in the *bgal1-1* mutant was lower than WT plant since it lacks BGAL1 but activities of other β-galactosidases were still detectable. All of these β-galactosidase activities were suppressed by galactosyrin produced from WT *P. syringae*.

bioRxiv preprint doi: https://doi.org/10.1101/2025.02.13.638044; this version posted February 14, 2025. The copyright holder for this preprint (which was not certified by peer review) is the author/funder, who has granted bioRxiv a license to display the preprint in perpetuity. It is made available under aCC-BY 4.0 International license.

Target	Enzyme (Source)	IC50 (μM)
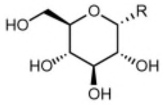	**α-glucosidase**	
	(Rice)	3.2
	(Rat intestinal maltase)	13
	(Human lysosome)	10
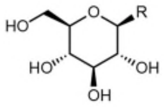	**β-glucosidase**	
	(Almond)	2
	(Bovine liver)	NA (29%)
	(Human lysosome)	NA (15%)
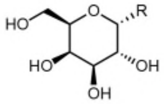	**α-galactosidase**	
	(Coffee beans)	NA (33%)
	(Human lysosome)	NA (37%)
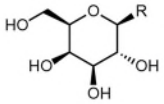	**β-galactosidase**	
	(*E. coli*)	2.3
	(Bovine liver)	NA (30%)
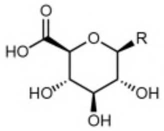	**β-glucuronidase**	
	(*E. coli*)	NA (4%)
	(Bovine liver)	NA (7%)
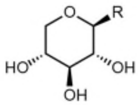	**β-xylosidase**	
	(*T. longibrachiatum*)	NA (20%)
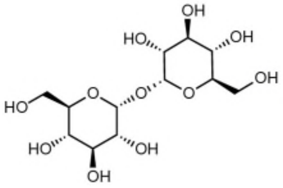	**Trehalase**	
	(Porcine kidney)	NA (10%)
	(Rat intestinal)	NA (21%)

Fig. S17. Galactosyrin inhibits different glycosidases.

Half maximal inhibitory concentration (IC_{50}) of galactosyrin inhibition of various glycosidases. NA denotes less than 50% inhibition at 100 µM galactosyrin, with numbers in brackets showing percent inhibition observed at 100 µM galactosyrin. Enzymes sources are colored: green:plants, purple:mammals and black:microbes.

bioRxiv preprint doi: https://doi.org/10.1101/2025.02.13.638044; this version posted February 14, 2025. The copyright holder for this preprint (which was not certified by peer review) is the author/funder, who has granted bioRxiv a license to display the preprint in perpetuity. It is made available under aCC-BY 4.0 International license.

Materials and methods

Bacterial strains, culture conditions and growth media - Bacterial strains and plasmids used in this study are listed in **Table S3** and **Table S4**, respectively. Generally, *P. syringae* and *A. tumefaciens* were grown at 28°C and *E. coli* were grown at 37°C. Liquid cultures were incubated with shaking at 220 rpm. Growth media were made as follows. LB medium: 10g/L tryptone, 5 g/L yeast extract, 10 g/L NaCl. Mannitol glutamate (MG) virulence-inducing minimal medium (*38*): 10 g/L mannitol, 2 g/L L-glutamic acid, 0.5 g/L KH_2PO_4, 0.2 g/L NaCl, 0.2 g/L $MgSO_4.7H_2O$, 50 µM iron citrate, adjusted to pH 5.5. M9 fructose medium: 6 g/L Na_2HPO_4, 3 g/L KH_2PO_4, 0.5 g/L NaCl, 1 g/L NH_4Cl, 0.1 mM $CaCl_2$, 2 mM $MgSO_4$, 22 mM fructose, adjusted to pH 5.5. Solid medium for plating contains 1.5% (w/v) agar. Antibiotics were generally used at the concentrations of 25 µg/ml rifampicin, 12.5 µg/ml gentamicin, 50 µg/ml kanamycin, 10 µg/ml tetracycline, 100 µg/ml carbenicillin, 25 µg/ml chloramphenicol. For purine auxotrophs (Δ*purF* and Δ*purD* mutants), cultures were supplemented with 0.2 mM adenine, guanine and thiamine.

Bacterial conjugation - Overnight bacterial cultures in LB medium were collected by centrifuging at 4,000 × g for 5 minutes, washed and resuspended in LB medium with no antibiotic. The recipient strain (*P. syringae* to be transformed), donor strain (*E. coli* containing plasmid constructs) and helper strain (*E. coli* pRK2013) were mixed in a ratio of 7:3:1 and centrifuged at 4,000 × g for 5 minutes to form a pellet which was spotted on to LB agar without antibiotics and incubated at 28°C overnight. The bacterial mixture was then resuspended and plated on LB agar with 25 µg/ml nitrofurantoin to select against *E. coli* and appropriate antibiotics to select for the desired transformants.

Forward genetic screen for galactosyrin mutants - A random transposon insertion mutant library was created by delivering mini-Tn5 transposon into the recipient *P. syringae* strain carrying a plasmid with constitutively expressed *lacZ* β-galactosidase gene (WT *lacZ*) through triparental mating conjugation with the *E. coli* donor strain (mini-Tn5 transposon) and helper strain (pRK2013). The resulting mutant library was plated on MG agar supplemented with 12.5 µg/ml each of rifampicin, gentamicin and kanamycin, 0.1 mg/ml X-gal (5-bromo-4-chloro-3-indolyl-beta-D-galacto-pyranoside) and 0.1 mM IPTG. Candidate galactosyrin inhibitor mutant colonies were identified by their darker blue colour from the higher activity of LacZ cleaving X-gal in the absence of galactosyrin. To validate the mutants, these candidate colonies were grown in MG medium overnight and the supernatant was heat inactivated at 95°C for 5 minutes and used in FDG assay to confirm the lack of inhibitor production. Then, inverse PCR was performed to amplify the transposon insertion site. Briefly, a small amount of bacteria was resuspended in water and heated at 95°C for 5 minutes to release DNA, which was restriction digested with Fast Digest PaeI (Thermo Scientific) at 37°C for 1 hour, heat inactivated at 65°C for 20 minutes, then self-ligated by adding ATP and T4 DNA ligase (Thermo Scientific) at 16°C for 18 hour and heat inactivated at 65°C for 20 minutes. The ligated DNA was used in a PCR with Q5 high-fidelity DNA polymerase (New England Biolabs) with GC

bioRxiv preprint doi: https://doi.org/10.1101/2025.02.13.638044; this version posted February 14, 2025. The copyright holder for this preprint (which was not certified by peer review) is the author/funder, who has granted bioRxiv a license to display the preprint in perpetuity. It is made available under aCC-BY 4.0 International license.

enhancer using primer pair oNS22/oNS23 (**Table S5**). PCR products were treated with Illustra ExoProStar (Cytiva) and Sanger sequenced (Eurofins, Luxembourg) using primer oNS15 (**Table S5**). To identify transposon insertion sites in the genome, the sequence adjacent to the transposon boundary was BLAST searched against the *P. syringae* pv. *tomato* DC3000 genome using The Pseudomonas Genome Database (pseudomonas.com) (*39*).

Galactosyrin production from bacterial culture - Bacterial cells were collected from an overnight culture in LB medium by centrifugation at 4,000 × g for 5 minutes, washed with sterile water, resuspended in MG medium to an OD_{600} of 0.5 and grown overnight at 28 °C (for both *P. syringae* and *E. coli*). Then the culture was centrifuged again and the supernatant containing the inhibitor was collected.

FDG assay for β -galactosidase activity and inhibition - For LacZ assays, the reaction was set up in Z buffer (60 mM Na_2HPO_4, 40 mM NaH_2PO_4, 10 mM KCl, 1 mM $MgSO_4$) with 0.2 µM FDG (Fluorescein di-β-D-galactopyranoside, Marker Gene Technologies, M0250) and 0.1 mU/µl LacZ (β-galactosidase enzyme from *E. coli*, Sigma, G6008). For inhibition tests, inhibitor samples made up half the reaction volume. For apoplastic fluid assays, apoplastic fluid from *N. benthamiana* was incubated with 0.2 µM FDG and 50 mM MES pH 5.5. Fluorescence signal from fluorescein, a product of FDG cleavage by β-galactosidase, was measured with 485 nm excitation and 535 nm emission every minute at 25°C using the plate reader Infinite M200 (Tecan). β-galactosidase activity was calculated from the rate of fluorescence increase over time. For *in planta* assays, 10 µM FDG was infiltrated into *N. benthamiana* leaves as spots then the leaves were imaged using Amersham Typhoon 5 (Cytiva) with 488 nm excitation laser and Cy2 emission filter (525BP20) at 300 PMT. Images were analysed using Fiji (*40*). β-galactosidase activity was calculated from the integrated density of fluorescence signal from each spot subtracted by a background from an uninfiltrated spot on each leaf.

Generation of gene deletion mutants - Deletion of the gene of interest in *P. syringae* genome was generated by the two-step allelic exchange method (*41*). For plasmid construct generation, fragments of around 1 kb flanking each side of the target gene were PCR amplified from genomic DNA using Q5 high fidelity DNA polymerase (New England Biolabs) with primer pairs L_F/L_R (left flanking fragment) and R_F/R_R (right flanking fragment) (**Table S5**). The plasmid backbone was PCR amplified from the suicide vector pK18mobsacB (*42*) using Q5 high fidelity DNA polymerase (New England Biolabs) with primer pair oNS34/oNS35 (**Table S5**) then assembled with the flanking fragments by Gibson assembly and transformed into *E. coli* TOP10. The resulting constructs were checked for correct assembly by Sanger sequencing using primers oNS50 and oNS51 (**Table S5**). The validated plasmids were transformed into *P. syringae* by triparental mating conjugation with the helper strain (pRK2013). The resulting merodiploid transformants were counter-selected on LB agar plates containing 10% (w/v) sucrose. Then, colonies were screened by colony PCR using primer pairs L_F/R_R (**Table S5**) to confirm the deletion mutant with the expected size of the deleted genomic region.

bioRxiv preprint doi: https://doi.org/10.1101/2025.02.13.638044; this version posted February 14, 2025. The copyright holder for this preprint (which was not certified by peer review) is the author/funder, who has granted bioRxiv a license to display the preprint in perpetuity. It is made available under aCC-BY 4.0 International license.

Generation of *gsn* cluster expression constructs - The sequence of the *gsn* cluster was PCR amplified from *P. syringae* WT genomic DNA using Q5 high fidelity DNA polymerase (New England Biolabs) with primer pairs oNS68/oNS63 for the *gsn* cluster with native promoter, or oNS206/oNS63 for the *gsn* cluster with no promoter (**Table S5**). The pBBR1MCS plasmid backbones (*43*) were linearised by restriction digest with Fast Digest SmaI and NsiI (Thermo Scientific) for pBBR1MCS2 (*l*ac promoter removed for insertion of *gsn* cluster with native promoter), or SmaI for pBBR1MCS3 (for insertion of *gsn* cluster with no promoter downstream of *lac* promoter) then assembled with the gene fragment by Gibson assembly and transformed into *E. coli* TOP10. The resulting constructs were checked for correct assembly by Sanger sequencing using primers seq_F and seq_R (**Table S5**). The validated plasmids were transformed into *P. syringae* by triparental mating conjugation with the helper strain (pRK2013).

Reverse transcriptase polymerase chain reaction (RT-PCR) - *P. syringae* cells were collected from LB agar plates grown at 28°C for 2 days, washed with sterile water, resuspended at an OD600 of 1 in MG medium and incubated at 28°C with shaking for 6 hours to induce virulence gene expression. Then, the cells were collected by centrifuging at 12,000 × g for 5 min, frozen with liquid nitrogen and lysed with lysozyme (5 mg/ml, Sigma) in TE buffer (10 mM Tris-HCL pH 7.4, 1 mM EDTA) at 25°C for 10 minutes while shaking. Total RNA was extracted from the sample using Monarch Total RNA miniprep kit (New England Biolabs), treated with Turbo DNA-free kit (Invitrogen) to remove genomic DNA followed by cDNA synthesis with GoScript reverse transcriptase master mix with random primers (Promega). PCR was then performed using GoTaq green master mix (Promega) with primer pairs F/R listed in **Table S5**. The PCR product was detected upon separation in 4% (w/v) agarose gel electrophoresis and staining with ethidium bromide. The no RT controls were performed on equivalent samples without reverse transcriptase to ensure that no amplification was detected from genomic DNA contamination.

Generation of Tn7 lux reporter constructs - The Tn7 lux reporter construct (pNS161) was derived from pRS-pOXB20:lux (*44*) by removing the OXB20 promoter by restriction digest with Ecl136II and Eco105I (Thermo Scientific) and replacing with T0T1 terminators amplified from pRS-pOXB20:lux using primer pair oNS386/oNS387 (**Table S5**). To generate the promoter:lux reporter fusion, the sequence of the promoter of interest was PCR amplified from *P. syringae* WT genomic DNA using Q5 high fidelity DNA polymerase (New England Biolabs) with primer pairs F/R (**Table S5**) and assembled into the Ecl136II restriction site in pNS161 by Gibson assembly and transformed into *E. coli* TOP10. The resulting constructs were checked for correct assembly by Sanger sequencing using primers oNS384 and oNS385 (**Table S5**). The validated plasmids were used in a four-parental mating conjugation with helper strains (pRK2013 and pUXBF13) to integrate the Tn7 transposon construct into the genome of *P. syringae*.

Luminescence reporter activity in planta - *P. syringae* strains with Tn7 lux reporter constructs were collected from an overnight culture in LB medium by centrifuging at 4,000 × g for 5 minutes, washed with 10 mM $MgCl_2$ and resuspended in 10 mM $MgCl_2$ at the OD_{600} of 1 (for imaging the same day) or

bioRxiv preprint doi: https://doi.org/10.1101/2025.02.13.638044; this version posted February 14, 2025. The copyright holder for this preprint (which was not certified by peer review) is the author/funder, who has granted bioRxiv a license to display the preprint in perpetuity. It is made available under aCC-BY 4.0 International license.

0.001 (for imaging after 3 days). Bacterial suspensions were infiltrated into *N. benthamiana* leaves using a needleless syringe. Then, leaves were detached from the plant, placed in a plastic Petri dish with wet tissue paper, left in the dark for 5 minutes and imaged for luminescence signal using Imagequant LAS-4000 imager (Cytiva).

Infection assay - *P. syringae* cells were collected from an overnight culture in LB medium by centrifuging at 4,000 × g for 5 minutes, washed with sterile water and resuspended in sterile water at the OD_{600} of 0.2 (~10^8 CFU/ml) with 0.04% (v/v) Silwet L-77. The bacterial suspension was then sprayed on both surfaces of 4-5 weeks-old *N. benthamiana* plant leaves until runoff. The infected plants were placed in a transparent plastic box to keep high humidity and maintained in a growth chamber at 21°C with 12-hour photoperiod and light intensity of 100 µmol/m^2/s. At 3 days post infection, leaf discs were then collected from infected tissue, surface sterilised with 70% (v/v) ethanol, washed with sterile water and homogenised in 1 ml of sterile water for serial dilution plating. Colonies were counted to calculate the number of bacterial colony forming units (CFU) per cm^2 area of infected tissue. The bacterial suspension used for inoculation was also plated to confirm the bacterial inoculum.

Bacterial growth curve *in vitro* - *P. syringae* cells were collected from an overnight culture in LB medium by centrifuging at 4,000 × g for 5 minutes, washed with sterile water and resuspended in sterile water at the OD_{600} of 0.05 in 150 µl of media in each well of 96 well plate. Bacterial growth was monitored with optical density at 600 nm (OD600) taken every 30 minutes using the plate reader Infinite M200 (Tecan) maintained at 28°C with shaking.

Generation of recombinant protein production constructs - The sequence of the gene of interest was PCR amplified from *P. syringae* WT genomic DNA using Q5 high fidelity DNA polymerase (New England Biolabs) with primer pairs F/R (**Table S5**). The pET-28b plasmid backbone was PCR amplified using Q5 high fidelity DNA polymerase (New England Biolabs) with primer pair oNS207/oNS208 (**Table S5**) then assembled with the gene fragments by Gibson assembly and transformed into *E. coli* TOP10. The resulting constructs were checked for correct assembly by Sanger sequencing using primers oNS209 and oNS210 (**Table S5**). The validated plasmids were then transformed into *E. coli* expression strains.

Enzyme purification - *E. coli* expression strains were grown in LB medium with appropriate antibiotics at 37°C overnight, then diluted in a fresh LB medium to OD600 of 0.04 and grown until OD600 of 0.6, then IPTG was added (0.4 mM) to induce protein expression at 20°C overnight. For GsnB, 500 uM L-rhamnose was also added. Then, bacterial cells were collected by centrifugation and lysed with CelLytic Express (Sigma). Strep-tagged enzymes from the lysate were isolated with Strep-Tactin XT 4Flow resin (IBA Life Sciences), washed with TBS buffer (50 mM Tris, 150 mM NaCl, pH 7.5) and eluted with TBS buffer with 50 mM biotin. Protein concentration was measured using Bradford assay (Sigma) with BSA (bovine serum albumin) standards.

bioRxiv preprint doi: https://doi.org/10.1101/2025.02.13.638044; this version posted February 14, 2025. The copyright holder for this preprint (which was not certified by peer review) is the author/funder, who has granted bioRxiv a license to display the preprint in perpetuity. It is made available under aCC-BY 4.0 International license.

Galactosyrin biosynthesis pathway reconstruction *in vitro* - The reaction was set up in TBS buffer (50 mM Tris, 150 mM NaCl, pH 7.5) with 100 mM NH_4Cl, 1 mM $MgCl_2$, 1 mM PRPP (5-Phospho-D-ribose 1-diphosphate), 1 mM NAD^+ (nicotinamide adenine dinucleotide), 1 mM NADPH (nicotinamide adenine dinucleotide phosphate) and biosynthesis enzymes (0.2 µM PurF, 0.2 µM GsnB, 0.4 µM GsnC and 2.6 µM GsnA). For each enzyme omission, an equivalent amount of GFP was used instead. The reaction mixture was incubated at 25°C for 20 hours to produce galactosyrin. For stepwise reaction, the first reaction was heat inactivated at 95°C for 5 minutes before the components of the second reaction was added.

GsnA assay - The reaction was set up in TBS buffer (50 mM Tris, 150 mM NaCl, pH 7.5) with 1 mM NAD^+, 1 mM 1ADR (1-amino-1-deoxy-ribitol) and 10 µM GsnA. Enzyme activity was monitored by measuring the formation of NADH with 340 nm absorbance every minute at 25°C using the plate reader Infinite M200 (Tecan). For metal cofactor preference test, GsnA was pretreated with 100 µM EDTA to chelate divalent metal ions. Then, the assay was supplemented with or without 1 mM $CoCl_2$ or $ZnCl_2$.

Isolation of 1ADR - Bacterial culture supernatant prepared the same way as galactosyrin production was mixed with 50% volume methanol and 20% volume chloroform, centrifuged at 10,000 × g for 5 minutes, then the top aqueous fraction was collected. 1ADR from the fraction was isolated with Amberlyst 15 hydrogen form cation exchange resin (Supelco), washed with 70% (v/v) ethanol followed by water, and eluted with 1M NH_4OH. The eluate was freeze dried then resuspended in water.

Protein structure modeling - GsnB (PSPTO_0835) structure was predicted with Alphafold2 through ColabFold (*63*) with default settings. The first ranked model was used. ChimeraX was used to visualise protein structures and matchmaker was used for structure comparison (*64*).

Inhibitor dose response curve - β -galactosidase activity was measured with the FDG assay as described above in the presence of different concentrations of each inhibitor. The activity was normalised with a no inhibitor control set as 100%. Then, drc package (*65*) was used to fit a four-parameter logistic model with a lower limit of 0 and upper limit of 100 to calculate the half maximal inhibitory concentration (IC_{50}).

***N. benthamiana* infection** - *P. syringae* cells were collected from an overnight culture in LB medium by centrifuging at 4,000 × g for 5 minutes, washed and resuspended in sterile water at the OD_{600} of 0.0002 (~10^5 CFU/ml). The bacterial suspension was then infiltrated into leaves of 4-5 weeks-old *N. benthamiana* plants. Infected plants were maintained in a growth chamber at 21°C with 12-hour photoperiod and light intensity of 100 µmol/m^2/s. Samples were collected after 3 days for GC-MS metabolomics or 5 days for lectin blot.

Apoplastic fluid extraction - *N. benthamiana* leaves were rolled into a 50 ml syringe filled with ice-cold water. The leaves were infiltrated by pulling the plunger to apply negative pressure drawing air out of the leaf tissue then pushing the plunger to apply positive pressure forcing water into the leaf tissue, while keeping the syringe tip sealed. The infiltrated leaves were blotted dry and rolled into a 20 ml syringe barrel

bioRxiv preprint doi: https://doi.org/10.1101/2025.02.13.638044; this version posted February 14, 2025. The copyright holder for this preprint (which was not certified by peer review) is the author/funder, who has granted bioRxiv a license to display the preprint in perpetuity. It is made available under aCC-BY 4.0 International license.

placed inside a 50 ml conical centrifuge tube. Apoplastic fluid was collected from the leaves by centrifugation at 900 × g for 15 minutes at 4°C.

Lectin blot - Proteins were collected by mixing apoplastic fluids with 4 volumes of acetone and 20 mM NaCl, incubating at room temperature for 15 minutes, then centrifuging at 12,000 × g for 10 minutes. The protein pellet was briefly air dried and resuspended in gel loading buffer (100 mM Tris-Cl (pH 6.8), 200 mM DTT, 4% (w/v) SDS, 0.2% (w/v) bromophenol blue, 20% (v/v) glycerol) then heated at 95°C for 5 minutes before being separated in 12% (w/v) polyacrylamide gel electrophoresis and transferred onto PVDF membrane using Trans-Blot Turbo system (Bio-Rad). The membrane was blocked with Carbo-Free blocking solution (Vector Laboratories) with 0.05% (v/v) Tween-20, then incubated with 5 µg/ml biotinylated RCA I lectin (Ricinus Communis Agglutinin I, RCA120, Vector Laboratories) in PBS buffer (10 mM sodium phosphate buffer, 3 mM KCl, 140 mM NaCl, pH 7.4) with 0.05% (v/v) Tween-20 (PBST), then washed with PBST, then incubated with Streptavidin-HRP (Sigma) (1:5,000x dilution in 5% BSA in PBST), and then washed with PBST. Chemiluminescence was detected using Clarity ECL western blotting substrates (Bio-Rad) with an Imagequant LAS-4000 imager (Cytiva). The membrane was then stained with Ponceau S for total protein detection.

Untargeted metabolomics - Apoplastic fluid samples (200 µl) were spiked with 20 µl of 2 µg/ml ribitol as an internal standard and analysed with GC-MS as described in GC-MS section. GC-MS data were processed using MassHunter Unknown Analysis (Agilent). Metabolite peaks were detected using TIC analysis with default settings, then mass spectra were searched against NIST11 (*66*) and Golm (*67*) library to putatively assign compound identifications to hits with match factors of at least 90. Peaks of the same metabolite feature were aligned using hierarchical clustering with a retention time window of 0.06. Peaks also detected in solvent blank samples were excluded. Metabolite features not robustly detected in all samples were removed. Peak area of each metabolite feature was log2 transformed and normalised with the peak of ribitol internal standard in each sample. Differentially accumulating metabolites were identified using Welch's t-test with p-values adjusted using Benjamini-Hochberg method to correct for multiple comparisons.

Estimation of apoplastic metabolite concentrations - Apoplastic fluid samples and metabolite standards at different concentrations (50 µl) were spiked with 5 µl of 0.1 mg/ml ribitol as an internal standard and analysed with GC-MS as described in GC-MS section. The peak area of each metabolite was normalised with the peak area of ribitol in each sample. A standard curve was constructed from the normalized peak areas and the corresponding concentrations of standards then linear regression was used to calculate the unknown concentration of the metabolite in apoplastic fluid samples based on their normalized peak area. Since apoplastic fluids extracted from leaves were diluted compared to the original plant apoplast in the leaf tissue, metabolite concentrations in the plant apoplast was estimated by

bioRxiv preprint doi: https://doi.org/10.1101/2025.02.13.638044; this version posted February 14, 2025. The copyright holder for this preprint (which was not certified by peer review) is the author/funder, who has granted bioRxiv a license to display the preprint in perpetuity. It is made available under aCC-BY 4.0 International license.

multiplying the concentration in the apoplastic fluid samples by a dilution factor of 2.7 ± 0.3, determined based on the method from (*68*).

Glycosidase inhibition assay - The enzymes β-glucosidase (from almond and bovine liver), α-galactosidase (from coffee beans), β -galactosidase (from *E. coli* and bovine liver), β -glucuronidases (from *E.coli* and bovine liver), β -xylosidase (*T. longibrachiatum*), *p*-nitrophenyl glycosides, and various disaccharides were purchased from Sigma-Aldrich Co. Brush border membranes were prepared from the rat small intestine according to the method of Kessler et al. (*69*) and were assayed at pH 6.8 for rat intestinal maltase using maltose. The inhibitory activity toward human lysosomal acid glycosidases was measured with Myozyme, Cerezyme, and Fabrazyme (Genzyme) as the enzyme source and an appropriate 4-methylumbelliferyl-glycopyranoside (Sigma-Aldrich) as substrate. The reaction mixture consisted 100 mM McIlvaine buffer (pH 5.2), 0.25% sodium taurocholate and 0.1% Triton X-100 (Nacalai Tesque Inc), and the appropriate amount of enzyme. The reaction mixture was pre-incubated at 0˚C for 45 min, and the reaction was started by the using 3 mM substrate solution, followed by incubation at 37˚C for 30 min. The reaction was stopped by the addition of 1.6 mL of the solution of 400 mM Glycine-NaOH solution (pH 10.6). The released 4-methylumbelliferone was measured (excitation 362 nm, emission 450 nm) with a F-4500 fluorescence spectrophotometer (Hitachi). For rat intestinal maltase activity, the reaction mixture contained 25 mM maltose, and the appropriate amount of enzyme, and the incubations were performed for 10 min at 37 ˚C. The reaction was stopped by heating at 100 ˚C for 3 min. After centrifugation (600 g; 10 min), the resulting reaction mixture was added to the Glucose CII-test Wako (Wako Pure Chemical Ind.). The absorbance at 505 nm was measured to determine the amount of the released D-glucose. Other glycosidase activities were determined using an appropriate *p*-nitrophenyl glycoside as substrate at the optimum pH of each enzyme. The reaction mixture contained 2 mM of the substrate and the appropriate amount of enzyme. The reaction was stopped by the addition of 400 mM Na_2CO_3. The released *p*-nitrophenol was measured spectrometrically at 400 nm.

Phylogenetic analysis

For each tree, the sequences described below were aligned using MAFFT 7 with L-INS-i algorithm (*45*). The resulting multiple sequence alignment was used to construct a maximum likelihood phylogenetic tree using IQ-TREE 2 (*46*) with the best-fit amino acid substitution model from ModelFinder (*47*). Branch support values were calculated using ultrafast bootstrap with 1,000 replications. Phylogenetic trees were visualised using iTOL (*48*).

Phylogeny of *P. syringae* - The *P. syringae* tree was constructed using concatenated coding DNA sequences of 4 conserved housekeeping genes (*rpoD* encoding sigma factor 70, *gyrB* encoding DNA gyrase B, *gltA* encoding citrate synthase and *gapA* encoding glyceraldehyde-3-phosphate dehydrogenase), identified through a local BLAST+ search (*49*) against selected genomes obtained from publicly available

bioRxiv preprint doi: https://doi.org/10.1101/2025.02.13.638044; this version posted February 14, 2025. The copyright holder for this preprint (which was not certified by peer review) is the author/funder, who has granted bioRxiv a license to display the preprint in perpetuity. It is made available under aCC-BY 4.0 International license.

NCBI (*50*) and pseudomonas.com databases (*39*). *P. syringae* genomes used in the analysis were selected to include all strains that have the *gsn* gene cluster identified from a BLAST search of GsnA amino acid sequence from *P. syringae* pv. *tomato* DC3000 (on www.blast.ncbi.nlm.nih.gov) with default settings, 62 type and pathotype strains (*51*) and additional strains to represent different phylogroups. Strains that have incomplete assembly or annotation at the site of the 4 housekeeping genes were discarded. *P. fluorescens* SBW25 was included as an outgroup. Phylogroups were assigned according to (*52*). For strains that contain the *gsn* cluster, the *gsn* cluster together with 20 kb of genomic region flanking each side was displayed. Gene model annotation of the genome was acquired from the RefSeq database (*53*) and gene family assignment was based on conserved domain database entry (CDD) linked to each gene (*54*). Many available genomes were not fully assembled so fragmented genomic regions and incompletely annotated genes are common.

Phylogeny of gsnA - The phylogenetic tree was constructed using coding DNA sequences of homologs of *gsnA* identified in *P. syringae* strains from above. A homolog of *gsnA* from *P. fluorescens* Pf275 was included as an outgroup since *P. fluorescens* SBW25 does not contain the *gsn* cluster.

Phylogeny of GsnA homologs - The amino acid sequence of GsnA from *P. syringae* pv. *tomato* DC3000 was used as a query for a PSI-BLAST search (on www.blast.ncbi.nlm.nih.gov)(*55*) on RefSeq database with default settings over 3 iterations to obtain 1,000 proteins, which were then filtered to include only hits with a length of 300-400 amino acids and at least 80% query coverage. For brevity, only a maximum of 5 homologs from each species were used as representatives. The sequence of threonine dehydrogenase from *E. coli* (WP_000646007.1) was included as an outgroup. Putative gene clusters were assigned by identifying a stretch of consecutive genes with the same orientation on the same strand of the genome as the gene encoding the GsnA homolog, both up and downstream until there was a gene with an opposite orientation. Gene model annotation of the genome was acquired from the RefSeq database (*53*) and gene family assignment was based on conserved domain database entry (CDD) linked to each gene (*54*). The association with transposable elements was defined as the presence of transposable elements within five genes distance from the first and last gene in the cluster. Many available genomes were not fully assembled so fragmented genomic regions and incompletely annotated genes are common.

Capture of enzyme-inhibitor complex

Galactosyrin production - *P. syringae* WT cells were collected from an overnight culture in LB medium by centrifugation at 4,000 × g for 5 minutes, washed and resuspended to the OD_{600} of 1 in M9 fructose medium to induce inhibitor production overnight at 28 °C. Then the culture was centrifuged again and the supernatant containing the inhibitor was collected, filtered through a 0.4 μm syringe filter and adjusted to pH 7.2 with NaOH. As a negative control, the equivalent sample from the galactosyrin-deficient Δ*gsn* mutant was also produced.

bioRxiv preprint doi: https://doi.org/10.1101/2025.02.13.638044; this version posted February 14, 2025. The copyright holder for this preprint (which was not certified by peer review) is the author/funder, who has granted bioRxiv a license to display the preprint in perpetuity. It is made available under aCC-BY 4.0 International license.

LacZ production - The *E. coli* expression strain (pNS141) was grown in LB medium with 50 ug/ml kanamycin at 37°C overnight, then diluted in fresh LB medium to OD600 of 0.04 and grown until OD600 of 0.6, then IPTG was added (0.4 mM) to induce protein expression at 28°C overnight. The bacterial cells were collected by centrifugation and lysed with CelLytic Express (Sigma). The lysate was mixed with imidazole (10 mM) before adding HisPur Ni-NTA resin (Thermo Scientific) and incubating at 4°C for 30 minutes to immobilize the His-tagged LacZ protein on the resin, which was then washed with 2xPBS (20 mM sodium phosphate buffer, 6 mM KCl, 280 mM NaCl, pH 7.4) with 25 mM imidazole.

Complex capture - The immobilized LacZ was loaded into a column then at least 10-fold volume of *P. syringae* supernatant from the first step was passed through the column to allow LacZ to capture galactosyrin to saturation. The column was then washed with 2xPBS with 25 mM imidazole before the complex was eluted with 1xPBS with 250 mM imidazole. For quality control, the eluted complex was run on SDS-PAGE to check the purity and quantity. To assess inhibitor saturation, the eluted complex was diluted 100 times and used in FDG assay to measure its enzyme activity compared to a negative control sample with no inhibitor.

Metabolite extraction - LacZ-galactosyrin complex capture was performed as above except the elution step. Instead, the immobilized complex was washed with water then with 17 mM acetic acid to denature the enzyme and release the metabolite.

Cryo-electron microscopy (cryo-EM)

Sample preparation - Purified LacZ β-galactosidase samples were refined using the Akta Pure chromatography system (Cytiva) with SuperDex 200 size exclusion chromatography column (Cytiva) and elution buffer with 25 mM Tris, 50 mM NaCl, 2 mM $MgCl_2$, 2 mM EDTA, 1 mM TCEP, pH 8. Protein concentration was measured with Nanodrop (Thermo Scientific) and adjusted to 0.7 mg/ml. Samples of native galactosyrin (WT) and negative control (Δ*gsn*) were prepared from enzyme-inhibitor complex capture. The sample with synthetic galactosyrin was prepared by adding 5 mM galactosyrin into a purified LacZ aliquot and incubating on ice for 30 minutes before applying onto grids.

Loading - An aliquot of 3 µl of control sample (Δ*gsn*) was applied onto a glow-discharged holey carbon copper grid (300 mesh, QUANTIFOIL® R 2/1). 3.2 µL of native inhibitor sample (WT) was applied onto a glow-discharged holey carbon copper grid (200 mesh, QUANTIFOIL® R 2/1). The grid was blotted and flash-frozen in liquid ethane with an FEI Mark IV Vitrobot. 3-3.5 µL of synthetic inhibitor sample was applied to a glow-discharged holey carbon copper grid (300 mesh, QUANTIFOIL® R 2/1). A Glacios Cryo-TEM was used to screen grids. CryoEM grids with optimal particle distribution and ice thickness were obtained by varying the blotting time/force. The Vitrobot chamber was set to 22 °C temperature and 100% humidity.

bioRxiv preprint doi: https://doi.org/10.1101/2025.02.13.638044; this version posted February 14, 2025. The copyright holder for this preprint (which was not certified by peer review) is the author/funder, who has granted bioRxiv a license to display the preprint in perpetuity. It is made available under aCC-BY 4.0 International license.

Imaging - Optimized cryo-EM grids for inhibitor-bound β-galactosidase were loaded onto a Titan Krios (Thermo Fisher Scientific Inc) operated at 300 keV in eBIC (electron Bio-Imaging Centre, Diamond). The inhibitor-bound dataset was collected with a Gatan Quantum post-column energy filter (Gatan Inc) operated in zero-loss mode with 20 eV slit width, paired with a Gatan K3 direct electron detector, using EPU by electron counting in super-resolution mode (bin 2) at a physical pixel size of 0.829 per pixel. Optimized cryo-EM grids for control β-galactosidase were loaded onto another Titan Krios (Thermo Fisher Scientific Inc) operated at 300 keV in eBIC (electron Bio-Imaging Centre, Diamond). The control dataset was collected with a Gatan Quantum post-column energy filter (Gatan Inc) operated in zero-loss mode with 20 eV slit width, paired with a Gatan K3 direct electron detector, using EPU by electron counting in super-resolution mode (bin 2) at a physical pixel size of 0.831 Å per pixel. Optimized cryo-EM grids for synthetic inhibitor-bound β-galactosidase were loaded onto a third Titan Krios (Thermo Fisher Scientific Inc) operated at 300 keV in eBIC (electron Bio-Imaging Centre, Diamond). The synthetic inhibitor-bound dataset was acquired with a Thermo Scientific Selectris X imaging filter (Thermo Fisher Scientific Inc) with 10 eV slit width, paired with the latest generation Thermo Scientific Falcon 4i direct electron detector, using EPU by super-resolution counting mode in EER format at a physical pixel size of 0.921 Å per pixel. For all 3 datasets, a total number of 40 frames were acquired for each exposure, giving a total dose of ~40 e−/Å^2/micrographs, with a defocus range between −0.5 and −2.1 µm. Details of data collection parameters are listed in **Table S6**.

Structure determination - The raw data was first corrected using a gain reference recorded immediately before the data acquisition, followed by generating a single micrograph that was corrected for overall drift using the MotionCor2 (*56*).The defocus value of each drift-corrected micrograph was determined by CTFFIND-4.1 (*57*) generating values ranging from −0.5 to −5.0 µm. Particle picking was done using the crYOLO program (*58*). Initially, a total of 3061973 particles were automatically picked from 15180 averaged images for native inhibitor-bound β-galactosidase, 1977809 particles from 11944 images for control β-galactosidase, and 2705966 particles from 10166 images for synthetic inhibitor-bound β-galactosidase, respectively.

All the 2D classification and 3D auto-refinement were done in RELION4.0 (*59*). The particles in 4k (super-resolution mode, bin 2) were boxed out in dimensions of 276 × 276 square pixels square for inhibitor-bound and control β-galactosidase, 250 × 250 square pixels square for synthetic inhibitor-bound β-galactosidase before further processing by the GPU accelerated RELION4.0. The first round 2D classification was done at a pixel size of around 2.39 Å for all 3 β-galactosidase samples. Several iterations of reference-free 2D classification were subsequently performed to remove bad particles (i.e., classes with fuzzy or un-interpretable features). 1406757, 1161378 and 2414813 good particles were re-extracted, and the second round 2D classification was done on the un-binned particles at pixel sizes of 0.829, 0.831 and 0.921 Å, yielding 659661, 593967 and 2353968 good particles for inhibitor-bound, control, and synthetic

bioRxiv preprint doi: https://doi.org/10.1101/2025.02.13.638044; this version posted February 14, 2025. The copyright holder for this preprint (which was not certified by peer review) is the author/funder, who has granted bioRxiv a license to display the preprint in perpetuity. It is made available under aCC-BY 4.0 International license.

inhibitor-bound β-galactosidase respectively. 588492 particles from synthetic inhibitor-bound β-galactosidase were used for 3D auto-refinement, for better comparison with the other 2 datasets. D2 symmetry was imposed throughout processing. The two half-maps of each dataset from this auto-refinement step were subjected to RELION's standard post-processing procedure. After auto-refinement and CTF refinement, the final maps of inhibitor-bound, control, and synthetic inhibitor-bound β-galactosidase achieved an averaged resolution of 1.93, 2.06, and 1.85 Å, respectively, based on RELION's gold-standard FSC with cutoff at 0.143. The synthetic inhibitor-bound β-galactosidase had reached its Nyquist resolution, so we re-extracted the 588492 particles using RELION reconstruct and further processed the synthetic inhibitor-bound β-galactosidase dataset in 8k (super-resolution mode, bin 1) at a pixel size of 0.4605 Å. After several iterations of 3D auto- refinement and CTF refinement, the final overall resolution for the synthetic inhibitor-bound β-galactosidase is 1.42 Å. Prior to visualization, all density maps were sharpened by applying a negative B-factor calculated by RELION post-process (*59*).

Model building and refinement - Atomic model building of the β-galactosidase protein chains were accomplished using ModelAngelo to build the initial β-galactosidase into our refined maps (*60*). For the inhibitor-bound structures, galactosyrin was manually fit into the map using COOT (*61*). Ions were then added to the models in COOT. Models refined via an iterative process using COOT and PHENIX real-space refinement (*61*, *62*). Water molecules were added using PHENIX douse function before final real-space refinement (*62*).

The cryoEM maps and corresponding PDB models for control (Δ*gsn*), native inhibitor-bound (WT), and synthetic inhibitor-bound have been deposited under accession number 8RI6, 8RI7, and 8RI8, and EMDB accession numbers EMD-19181, EMD-19182, and EMD-19183, respectively.

Gas chromatography-mass spectrometry (GC-MS)

Sample preparation - To extract soluble metabolites, sample was mixed with an equal volume of a mixture of methanol:chloroform (2.5:1 ratio), centrifuged at 12,000 × g for 5 minutes then the top aqueous fraction was collected and freeze dried. The dried sample was dissolved in pyridine with 20 mg/ml methoxyamine HCl (for carbonyl-containing compounds such as galactosyrin aldehyde and untargeted metabolomics) or pyridine only (for all other samples) and incubated at 37°C for 30 minutes. Then the sample was derivatised with MSTFA (N-Methyl-N-trimethylsilyl trifluoroacetamide) at 37°C for 30 minutes. The resulting mixture was centrifuged at 12,000 × g for 5 minutes and the supernatant was transferred into vials for GC-MS.

GC-MS - GC-MS was conducted using Intuvo 9000 GC system with 5977B single quadrupole MSD system and 7693 autosampler (Agilent). The GC system was equipped with a guard chip and two Agilent 122-5512UI-INT columns connected sequentially. The sample (0.5 µl) was injected in splitless mode at 230°C. Helium was used as carrier gas and the flow was set constant at 1 ml/min through the first column and 1.2 ml/min through the second column. The oven temperature was initially held at 70°C for 5

bioRxiv preprint doi: https://doi.org/10.1101/2025.02.13.638044; this version posted February 14, 2025. The copyright holder for this preprint (which was not certified by peer review) is the author/funder, who has granted bioRxiv a license to display the preprint in perpetuity. It is made available under aCC-BY 4.0 International license.

min then increased to 330°C at the rate of 7.5°C/min. The transfer line temperature was 250 °C. The sample was ionised by electron impact ionisation at 70 eV. The MS source temperature was 230°C and the MS quadrupole temperature was 150°C. The mass spectrometer was autotuned before running each batch of samples. The acquisition of mass spectra was in scan mode for ions from 50 to 600 mass units with the scan speed of n=2. Solvent delay of 7.5 min was set before acquisition. Data were analysed using MassHunter qualitative analysis software (Agilent).

Chemical standards - 1ADR (1-Amino-1-deoxy-D-ribitol hydrochloride, Supelco 72679, CAS no. 22566-17-2), galactosylglycerol ((2R)-Glycerol-O-β-D-galactopyranoside, Cayman Chemical CAY17218, CAS no. 16232-91-0), trehalose (Sigma T9531, CAS no. 6138-23-4)

Chemical synthesis

The synthesis scheme is outlined in **Fig. S13**.

Chemicals and other materials - All reactions were carried out under an argon atmosphere. Reagents and (dry) solvents were commercially obtained from Acros Organics, Merck, VWR Chemicals, Fisher Chemical and Carbolution Chemicals, compound **1** was obtained from AstaTech (P10778, CAS no. 153172-31-7). Flash chromatography was performed on silica gel from Thermo Scientific with a particle size of 60-200 µm and an average pore size of 60 Å. Thin layer chromatography (TLC) analysis was carried out on Merck $60F_{254}$ silica gel plates.

HPLC and mass spectrometry - Preparative HPLC reversed-phase chromatography was performed on a Shimadzu Prominence system with a LC-20AP pump equipped with a Phenomenex Luna® C18(2) 100Å column (5 µm, 100 x 21.2 mm). Analytical LC-MS (ESI) analysis was performed on a Thermo Scientific UltiMate 3000 HPLC system with a Thermo Finnigan LCQ Fleet ion trap mass spectrometer, using a gradient program with eluent A = MQ-water with 0.1% formic acid and eluent B = acetonitrile with 0.1% formic acid, 0.5 min 10% B, then 10% to 100% B in 5.5 min, then 3.2 min 100% B, a flow rate of 1.0 mL min^{-1} on a Macherey-Nagel NUCLEODUR C18 Pyramid column (particle size 5 µm, 250 mm x 4.0 mm) at 25 °C. HRMS (ESI) analyses were performed on a Thermo Scientific Exactive Plus EMR mass spectrometer.

Nuclear magnetic resonance - NMR spectra were recorded with a Bruker 400 MHz Avance II spectrometer, equipped with a PATXI probe, with a ^{1}H frequency of 399.99 MHz and ^{13}C frequency of 100.59 MHz. Residual ^{1}H resonance from the deuterated solvents was used to reference the ^{1}H spectra with the methyl resonance of tetramethylsilane (δ = 0.00 ppm), the ^{13}C spectra were referenced through the solvent ^{13}C resonance.

***tert*-Butyl (3a*R*,4*R*,6a*S*)-4-(hydroxymethyl)-2,2-dimethyl-tetrahydro-[1,3]dioxolo[4,5-*c*]pyrrole-5-carboxylate (2):** A solution of compound **1** (67.4 mg, 234 µmol, 1.0 equiv.) in a mixture of dry MeOH (600 µL) and triethylamine (200 µL) was treated with di-*tert*-butyl dicarbonate (76.6 mg, 351

bioRxiv preprint doi: https://doi.org/10.1101/2025.02.13.638044; this version posted February 14, 2025. The copyright holder for this preprint (which was not certified by peer review) is the author/funder, who has granted bioRxiv a license to display the preprint in perpetuity. It is made available under aCC-BY 4.0 International license.

µmol, 1.5 equiv.) and stirred for 2 hours at room temperature. The resulting mixture was quenched by the addition of EtOAc (20 mL) and saturated aqueous NH_4Cl (10 mL). The organic layer was separated, washed with 1 M hydrochloric acid (10 mL) and brine (10 mL), dried over Na_2SO_4, filtered, and concentrated *in vacuo*. The obtained crude residue was dissolved in a 1.0 M solution of *N*, *N*, *N*-tributylbutan-1-aminium fluoride (1.0 mL, 1.0 mmol, 4.3 equiv.) and stirred for 2.5 hours at room temperature. The resulting mixture was concentrated *in vacuo*, re-dissolved in DCM (10 mL), washed with saturated aqueous NH_4Cl (10 mL) and brine (10 mL), and concentrated *in vacuo*. The obtained crude residue was purified by flash column chromatography (6 g SiO_2, EtOAc/cyclohexane 1:9 → 1:1) to provide alcohol **2** as a clear colorless oil (38.6 mg, 60% over two steps). R_f = 0.3 (SiO_2, EtOAc/cyclohexane 1:1). HPLC retention time = 6.7 min. MS (ESI) *m/z* (%) = expected 273.16; (pos) 174.29 ($[M+2H\text{-}Boc]^+$, 46), 218.08 ($[M+2H\text{-}^tBu]^+$, 100), 273.92 ($[M+H]^+$, 4). ^1H NMR (400 MHz, DMSO-D_6) δ 4.97 – 4.86 (m, 1H), 4.72 – 4.65 (m, 1H), 4.65 – 4.60 (m, 1H), 3.90 – 3.72 (m, 1H), 3.57 – 3.43 (m, 2H), 3.43 – 3.34 (m, 2H), 1.39 (s, 9H), 1.33 (s, 3H), 1.24 (s, 3H). ^{13}C NMR (101 MHz, DMSO-D_6) (two rotamers) δ 153.5, 110.4, 82.4, 81.6, 79.1, 78.7, 78.6, 78.3, 65.0, 64.5, 60.8, 60.2, 52.8, 52.2, 28.1, 26.8, 24.7.

(2*S*,3*R*,4*S*)-2-(dihydroxymethyl)pyrrolidine-3,4-diol (3) via **2:** A solution of alcohol **2** (33.2 mg, 122 µmol, 1.0 equiv.) in wetted DCM (1 mL) was treated with Dess-Martin reagent (77.0 mg, 182 µmol, 1.5 equiv.) and stirred for 3 hours at room temperature. The resulting mixture was concentrated *in vacuo* and purified by preparative reversed phase chromatography (C18, acetonitrile/water with 0.1% TFA) to provide the intermediate aldehyde after drying *in vacuo* (HPLC retention time = 6.2 min. MS (ESI) *m/z* (%) = expected 271.14; (pos) 172.15 ($[M+2H\text{-}Boc]^+$, 100), 215.94 ($[M+2H\text{-}^tBu]^+$, 49)). The resulting intermediate aldehyde was immediately re-dissolved in a 2 M HCl solution in THF/water (1:1, 16.6 mL), stirred for 4.5 h at room temperature and subsequently concentrated *in vacuo*. The crude residue was purified by preparative reversed phase chromatography (C18, acetonitrile/water) to provide compound **4** as a slightly brownish solid (4.3 mg, 21% over two steps). HPLC retention time = 1.8 min. MS (ESI) *m/z* (%) = expected 149.07; (pos) 132.01 ($[M+H\text{-}H_2O]^+$, 28), 149.97 ($[M+H]^+$, 100). ^1H NMR (400 MHz, D_2O) δ 5.38 (d, J = 4.6 Hz, 1H), 4.45 – 4.37 (m, 1H), 4.36 (dd, J = 7.3, 4.2 Hz, 1H), 3.55 (dd, J = 7.2, 4.8 Hz, 1H), 3.47 (dd, J = 12.7, 3.8 Hz, 1H), 3.38 (dd, J = 12.7, 2.2 Hz, 1H). ^{13}C NMR (101 MHz, D_2O) δ 87.0, 71.6, 69.7, 64.6, 49.6.

Alternatively, (2*S*,3*R*,4*S*)-2-(dihydroxymethyl)pyrrolidine-3,4-diol (**3**) could also be obtained as a mixture via intermediate **4**: To this end, the crude compound **4**, obtained via the Kamath *et al.* procedure (*37*) using **1** (100 mg, 348 µmol, 1.0 equiv.) was dissolved in a 2 M HCl solution in THF/water (1:1, 6 mL) and stirred for 18 h at room temperature. The resulting mixture was concentrated *in vacuo* and purified by preparative reversed phase chromatography (C18, acetonitrile/water) to provide a brownish solid (10.2 mg), consisting predominantly (according to NMR) of compound **3**.

bioRxiv preprint doi: https://doi.org/10.1101/2025.02.13.638044; this version posted February 14, 2025. The copyright holder for this preprint (which was not certified by peer review) is the author/funder, who has granted bioRxiv a license to display the preprint in perpetuity. It is made available under aCC-BY 4.0 International license.

Full-length sequence of *gsnA* - *gsnA* (PSPTO_0834) was originally misannotated with a truncation. The recently updated locus tag PSPTO_RS04425 is correctly annotated. The full-length sequence is shown below.

>*gsnA*_CDS

ATGAAAGCACTGGGCTTAATGGATAACCAGAGGCTTGAACTTGTGGATTGCAAGGATCCTGT
CATGCTTGCTCCTGACGGCGTAGAAATCGATATCGTGCTATCAGGTATATGCGGAACTGATC
TGGCGGTATTGTCGGGCCGTGAAGGTGGAGAGGTGGGCATTATACGCGGGCACGAAGCAGT
TGGCATTATTATCGATGTAGGTAAGGATGTAGTACACCTACAAAAAGGGATGCGGGTGGTG
GTTGATCCCAACGAATACTGTGGCGTTTGCGAACCTTGCCGTCTTGCTAAAACGCACCTATG
CAATGGGGGGGTGAACGCTGGGTTGGATATCGCAGGTGTCAACAAACATGGAACTTTTGCC
GAGCGCTTCGTTACTCGTGAGCGTTTTGTGTATCAATTGCCAGACGATATGAGCTGGGCAGC
TGGTGTGTTGGTTGAGCCTGTTGCCTGCATTCTGAATAATATAGACCAGGCGTTCATTCGAGC
GGGAGAGCGTGTGTTGATCCTAGGGTCTGGCCCTATGAGTCTGATTGCGCAGATCGTTCTGC
GCTCAATGGGAGTTGACACGCTCGCCACTGATCGAAACACACATCGCATACAGTTCGGCCGC
TCACAAAGTCTTGATGTTATACATGCCGATGATCTTGAGTTGCAGATGCAGCACCAAGAAAA
GTTTGATGTTGTTATCGATACTGTCGGTAATCAGATCGATACAGCTTCACGCTACATCGGTCG
CGGTGGGAGAATTGTACTTTTTGGATTTGATAGTGACTATCACTACATGCTGCCTGTAAAGT
ACTTCCTGGTTAACGCTATCAGTATTATTTCTGCTGGAGAATACAATCAGCACTTTCCTAGAG
CAATTCGTCTTGTGCAAAAACTTCCTGAGCTAGGGCGGCTGGTAACGCATCGCTACGTACTA
GAAAATCACTCGGAGGTTTTCGATGCACTTCTGAACGATGCTTCCGCCCCCAATATAAAAAG
CGTATTCACACCAAATCTCGCTTATCTTTAA

>GsnA_protein

MKALGLMDNQRLELVDCKDPVMLAPDGVEIDIVLSGICGTDLAVLSGREGGEVGIIRGHEAVGII
IDVGKDVVHLQKGMRVVVDPNEYCGVCEPCRLAKTHLCNGGVNAGLDIAGVNKHGTFAERFV
TRERFVYQLPDDMSWAAGVLVEPVACILNNIDQAFIRAGERVLILGSGPMSLIAQIVLRSMGVDT
LATDRNTHRIQFGRSQSLDVIHADDLELQMQHQEKFDVVIDTVGNQIDTASRYIGRGGRIVLFGF
DSDYHYMLPVKYFLVNAISIISAGEYNQHFPRAIRLVQKLPELGRLVTHRYVLENHSEVFDALLN
DASAPNIKSVFTPNLAYL

bioRxiv preprint doi: https://doi.org/10.1101/2025.02.13.638044; this version posted February 14, 2025. The copyright holder for this preprint (which was not certified by peer review) is the author/funder, who has granted bioRxiv a license to display the preprint in perpetuity. It is made available under aCC-BY 4.0 International license.

Table S1 Tn5 insertion sites in galactosyrin-deficient mutants

Mutant	Site	PSPTO	hit
320	63170	0046	hypothetical protein
716	289486	0265	peptide ABC transporter permease
796	327456	0301	gabT-2 ,4-aminobutyrate aminotransferase
545	385528	0352	ntrC ,nitrogen regulation protein NR(I)
505	455021	0408	hypothetical protein
55	455662	0409	TldD/PmbA family protein
341	905189	NA	721 bp intergenic region
487	905266	NA	721 bp intergenic region
364	905304	NA	721 bp intergenic region
475	905581	0834	alcohol dehydrogenase
488	905939	0834	alcohol dehydrogenase
312	906245	0834	alcohol dehydrogenase
71	906842	0835	ribD C-terminal domain protein
422	907025	0835	ribD C-terminal domain protein
744	907033	0835	ribD C-terminal domain protein
179	907165	0835	ribD C-terminal domain protein
231	953659	0882	hypothetical protein
232	953659	0882	hypothetical protein
272	1134010	1034	wssI ,cell morphology protein
821	1236381	1122	MerR family transcriptional regulator
97	1270036	1156	isochorismatase family protein
519	1284728	1169	pta ,phosphate acetyltransferase
363	1326959	1211	membrane protein
500	1361947	1239	algE ,alginate biosynthesis protein AlgE
135	1492216	1355	ABC transporter ATP-binding protein
137	1492216	1355	ABC transporter ATP-binding protein
92	1521575	NA	1077 bp intergenic region
110	1521578	NA	1077 bp intergenic region
344	1522424	1379	hrpR ,type III transcriptional regulator HrpR
291	1522426	1379	hrpR ,type III transcriptional regulator HrpR
295	1522426	1379	hrpR ,type III transcriptional regulator HrpR
298	1522426	1379	hrpR ,type III transcriptional regulator HrpR

bioRxiv preprint doi: https://doi.org/10.1101/2025.02.13.638044; this version posted February 14, 2025. The copyright holder for this preprint (which was not certified by peer review) is the author/funder, who has granted bioRxiv a license to display the preprint in perpetuity. It is made available under aCC-BY 4.0 International license.

339	1522426	1379	hrpR ,type III transcriptional regulator HrpR
16	1522469	1379	hrpR ,type III transcriptional regulator HrpR
290	1522470	1379	hrpR ,type III transcriptional regulator HrpR
31	1522486	1379	hrpR ,type III transcriptional regulator HrpR
688	1522596	1379	hrpR ,type III transcriptional regulator HrpR
37	1522686	1379	hrpR ,type III transcriptional regulator HrpR
244	1522686	1379	hrpR ,type III transcriptional regulator HrpR
442	1522887	1379	hrpR ,type III transcriptional regulator HrpR
444	1522887	1379	hrpR ,type III transcriptional regulator HrpR
433	1522902	1379	hrpR ,type III transcriptional regulator HrpR
508	1522903	1379	hrpR ,type III transcriptional regulator HrpR
330	1522931	1379	hrpR ,type III transcriptional regulator HrpR
336	1522996	1379	hrpR ,type III transcriptional regulator HrpR
310	1523025	1379	hrpR ,type III transcriptional regulator HrpR
743	1523049	1379	hrpR ,type III transcriptional regulator HrpR
368	1523060	1379	hrpR ,type III transcriptional regulator HrpR
20	1523171	1379	hrpR ,type III transcriptional regulator HrpR
167	1523177	1379	hrpR ,type III transcriptional regulator HrpR
24	1523315	1380	hrpS ,type III transcriptional regulator HrpS
89	1523388	1380	hrpS ,type III transcriptional regulator HrpS
477	1523470	1380	hrpS ,type III transcriptional regulator HrpS
359	1523518	1380	hrpS ,type III transcriptional regulator HrpS
703	1523518	1380	hrpS ,type III transcriptional regulator HrpS
111	1523528	1380	hrpS ,type III transcriptional regulator HrpS
27	1523533	1380	hrpS ,type III transcriptional regulator HrpS
353	1523553	1380	hrpS ,type III transcriptional regulator HrpS
360	1523581	1380	hrpS ,type III transcriptional regulator HrpS
269	1523693	1380	hrpS ,type III transcriptional regulator HrpS
436	1523755	1380	hrpS ,type III transcriptional regulator HrpS
631	1523917	1380	hrpS ,type III transcriptional regulator HrpS
516	1523940	1380	hrpS ,type III transcriptional regulator HrpS
813	1523967	1380	hrpS ,type III transcriptional regulator HrpS
223	1524128	1380	hrpS ,type III transcriptional regulator HrpS

bioRxiv preprint doi: https://doi.org/10.1101/2025.02.13.638044; this version posted February 14, 2025. The copyright holder for this preprint (which was not certified by peer review) is the author/funder, who has granted bioRxiv a license to display the preprint in perpetuity. It is made available under aCC-BY 4.0 International license.

333	1542679	NA	243 bp intergenic region
247	1542809	NA	243 bp intergenic region
415	1542828	NA	243 bp intergenic region
48	1542876	1404	hrpL ,RNA polymerase sigma factor HrpL
538	1543014	1404	hrpL ,RNA polymerase sigma factor HrpL
531	1543189	1404	hrpL ,RNA polymerase sigma factor HrpL
511	1543233	1404	hrpL ,RNA polymerase sigma factor HrpL
331	1543339	1404	hrpL ,RNA polymerase sigma factor HrpL
677	1543342	1404	hrpL ,RNA polymerase sigma factor HrpL
108	1543347	1404	hrpL ,RNA polymerase sigma factor HrpL
582	1543348	1404	hrpL ,RNA polymerase sigma factor HrpL
354	1543352	1404	hrpL ,RNA polymerase sigma factor HrpL
329	1654324	1497	sensor histidine kinase/response regulator
772	1798122	1640	hypothetical protein
737	1810899	1649	autotransporter
526	1826908	NA	1094 bp intergenic region
42	1839403	1670	xfp ,xylulose-5-phosphate/fructose-6-phosphate phosphoketolase
210	1947971	1778	htpX ,heat shock protein HtpX
77	1948049	1778	htpX ,heat shock protein HtpX
87	1948049	1778	htpX ,heat shock protein HtpX
245	2051445	1878	ISPsy11, transposase OrfB
124	2051500	1878	ISPsy11, transposase OrfB
237	2078470	1902	bphP ,bacteriophytochrome histidine kinase
75	2194099	2009	hypothetical protein
747	2346315	2149	pyoverdine sidechain synthetase III, L-Thr-L-Ser component
67	2445610	2222	sensor histidine kinase
366	2445618	2222	sensor histidine kinase
446	2445702	2222	sensor histidine kinase
322	2445745	2222	sensor histidine kinase
62	2445863	2222	sensor histidine kinase
534	2445871	2222	sensor histidine kinase
490	2445923	2222	sensor histidine kinase
345	2445940	2222	sensor histidine kinase

bioRxiv preprint doi: https://doi.org/10.1101/2025.02.13.638044; this version posted February 14, 2025. The copyright holder for this preprint (which was not certified by peer review) is the author/funder, who has granted bioRxiv a license to display the preprint in perpetuity. It is made available under aCC-BY 4.0 International license.

82	2445950	2222	sensor histidine kinase
70	2446019	2222	sensor histidine kinase
218	2446176	2222	sensor histidine kinase
792	2446222	2222	sensor histidine kinase
248	2446283	2222	sensor histidine kinase
764	2446368	2222	sensor histidine kinase
266	2489809	NA	106 bp intergenic region
61	3421882	NA	447 bp intergenic region
259	3468913	3087	hopAB2 ,type III effector HopAB2
789	3567970	3176	thermolysin metallopeptidase
309	3619914	NA	618 bp intergenic region
506	3899841	3457	short-chain fatty acid transporter
503	4147127	3680	methyl-accepting chemotaxis protein
234	4390369	3878	ABC transporter substrate-binding protein
527	4421429	3906	hypothetical protein
281	4503514	3993	kup ,potassium uptake protein
308	4643117	4119	estC ,carboxylesterase
539	4759280	4225	nadB ,L-aspartate oxidase
546	4759393	4225	nadB ,L-aspartate oxidase
274	4759468	4225	nadB ,L-aspartate oxidase
549	4759759	4225	nadB ,L-aspartate oxidase
22	4787767	NA	308 bp intergenic region
133	4809323	4270	ISPsy4, transposition helper protein
134	4809323	4270	ISPsy4, transposition helper protein
497	4860239	4309	catI ,3-oxoadipate:succinyl-CoA transferase subunit A
770	4891781	4340	insecticidal toxin protein
98	4962478	4397	mutT/nudix family protein
343	5610705	4950	orn ,oligoribonuclease
323	5770050	5066	ISPsy8, transposase OrfB
91	5815028	5114	hypothetical protein
176	5876742	NA	41 bp intergenic region
502	6014135	5288	ilvA-2 ,threonine dehydratase
321	6058555	5330	hypothetical protein

bioRxiv preprint doi: https://doi.org/10.1101/2025.02.13.638044; this version posted February 14, 2025. The copyright holder for this preprint (which was not certified by peer review) is the author/funder, who has granted bioRxiv a license to display the preprint in perpetuity. It is made available under aCC-BY 4.0 International license.

541	6081806	5348	hypothetical protein
683	6205735	5453	oxidoreductase, aldo/keto reductase family
495	6256388	5489	cytosolic long-chain acyl-CoA thioester hydrolase family protein
44	6256408	5489	cytosolic long-chain acyl-CoA thioester hydrolase family protein
154	6264157	5499	aspA ,aspartate ammonia-lyase
452	6390782	5610	gidA ,glucose-inhibited division protein A
426	6390809	5610	gidA ,glucose-inhibited division protein A
840	6393312	5611	trmE ,tRNA modification GTPase TrmE
749	6393435	5611	trmE ,tRNA modification GTPase TrmE
320	63170	0046	hypothetical protein
716	289486	0265	peptide ABC transporter permease
796	327456	0301	gabT-2 ,4-aminobutyrate aminotransferase
545	385528	0352	ntrC ,nitrogen regulation protein NR(I)
505	455021	0408	hypothetical protein
55	455662	0409	TldD/PmbA family protein
341	905189	NA	721 bp intergenic region
487	905266	NA	721 bp intergenic region
364	905304	NA	721 bp intergenic region
475	905581	0834	alcohol dehydrogenase
488	905939	0834	alcohol dehydrogenase
312	906245	0834	alcohol dehydrogenase
71	906842	0835	ribD C-terminal domain protein
422	907025	0835	ribD C-terminal domain protein
744	907033	0835	ribD C-terminal domain protein
179	907165	0835	ribD C-terminal domain protein
231	953659	0882	hypothetical protein
232	953659	0882	hypothetical protein
272	1134010	1034	wssI ,cell morphology protein
821	1236381	1122	MerR family transcriptional regulator
97	1270036	1156	isochorismatase family protein
519	1284728	1169	pta ,phosphate acetyltransferase
363	1326959	1211	membrane protein
500	1361947	1239	algE ,alginate biosynthesis protein AlgE

bioRxiv preprint doi: https://doi.org/10.1101/2025.02.13.638044; this version posted February 14, 2025. The copyright holder for this preprint (which was not certified by peer review) is the author/funder, who has granted bioRxiv a license to display the preprint in perpetuity. It is made available under aCC-BY 4.0 International license.

135	1492216	1355	ABC transporter ATP-binding protein
137	1492216	1355	ABC transporter ATP-binding protein
92	1521575	NA	1077 bp intergenic region
110	1521578	NA	1077 bp intergenic region
344	1522424	1379	hrpR ,type III transcriptional regulator HrpR
291	1522426	1379	hrpR ,type III transcriptional regulator HrpR
295	1522426	1379	hrpR ,type III transcriptional regulator HrpR
298	1522426	1379	hrpR ,type III transcriptional regulator HrpR
339	1522426	1379	hrpR ,type III transcriptional regulator HrpR
16	1522469	1379	hrpR ,type III transcriptional regulator HrpR
290	1522470	1379	hrpR ,type III transcriptional regulator HrpR
31	1522486	1379	hrpR ,type III transcriptional regulator HrpR
688	1522596	1379	hrpR ,type III transcriptional regulator HrpR
37	1522686	1379	hrpR ,type III transcriptional regulator HrpR
244	1522686	1379	hrpR ,type III transcriptional regulator HrpR
442	1522887	1379	hrpR ,type III transcriptional regulator HrpR
444	1522887	1379	hrpR ,type III transcriptional regulator HrpR
433	1522902	1379	hrpR ,type III transcriptional regulator HrpR
508	1522903	1379	hrpR ,type III transcriptional regulator HrpR
330	1522931	1379	hrpR ,type III transcriptional regulator HrpR
336	1522996	1379	hrpR ,type III transcriptional regulator HrpR
310	1523025	1379	hrpR ,type III transcriptional regulator HrpR
743	1523049	1379	hrpR ,type III transcriptional regulator HrpR
368	1523060	1379	hrpR ,type III transcriptional regulator HrpR
20	1523171	1379	hrpR ,type III transcriptional regulator HrpR
167	1523177	1379	hrpR ,type III transcriptional regulator HrpR
24	1523315	1380	hrpS ,type III transcriptional regulator HrpS
89	1523388	1380	hrpS ,type III transcriptional regulator HrpS
477	1523470	1380	hrpS ,type III transcriptional regulator HrpS
359	1523518	1380	hrpS ,type III transcriptional regulator HrpS
703	1523518	1380	hrpS ,type III transcriptional regulator HrpS
111	1523528	1380	hrpS ,type III transcriptional regulator HrpS
27	1523533	1380	hrpS ,type III transcriptional regulator HrpS

bioRxiv preprint doi: https://doi.org/10.1101/2025.02.13.638044; this version posted February 14, 2025. The copyright holder for this preprint (which was not certified by peer review) is the author/funder, who has granted bioRxiv a license to display the preprint in perpetuity. It is made available under aCC-BY 4.0 International license.

353	1523553	1380	hrpS ,type III transcriptional regulator HrpS
360	1523581	1380	hrpS ,type III transcriptional regulator HrpS
269	1523693	1380	hrpS ,type III transcriptional regulator HrpS
436	1523755	1380	hrpS ,type III transcriptional regulator HrpS
631	1523917	1380	hrpS ,type III transcriptional regulator HrpS
516	1523940	1380	hrpS ,type III transcriptional regulator HrpS
813	1523967	1380	hrpS ,type III transcriptional regulator HrpS
223	1524128	1380	hrpS ,type III transcriptional regulator HrpS
333	1542679	NA	243 bp intergenic region
247	1542809	NA	243 bp intergenic region
415	1542828	NA	243 bp intergenic region
48	1542876	1404	hrpL ,RNA polymerase sigma factor HrpL
538	1543014	1404	hrpL ,RNA polymerase sigma factor HrpL
531	1543189	1404	hrpL ,RNA polymerase sigma factor HrpL
511	1543233	1404	hrpL ,RNA polymerase sigma factor HrpL
331	1543339	1404	hrpL ,RNA polymerase sigma factor HrpL
677	1543342	1404	hrpL ,RNA polymerase sigma factor HrpL
108	1543347	1404	hrpL ,RNA polymerase sigma factor HrpL
582	1543348	1404	hrpL ,RNA polymerase sigma factor HrpL
354	1543352	1404	hrpL ,RNA polymerase sigma factor HrpL
329	1654324	1497	sensor histidine kinase/response regulator
772	1798122	1640	hypothetical protein
737	1810899	1649	autotransporter
526	1826908	NA	1094 bp intergenic region
42	1839403	1670	xfp ,xylulose-5-phosphate/fructose-6-phosphate phosphoketolase
210	1947971	1778	htpX ,heat shock protein HtpX
77	1948049	1778	htpX ,heat shock protein HtpX
87	1948049	1778	htpX ,heat shock protein HtpX
245	2051445	1878	ISPsy11, transposase OrfB
124	2051500	1878	ISPsy11, transposase OrfB
237	2078470	1902	bphP ,bacteriophytochrome histidine kinase
75	2194099	2009	hypothetical protein
747	2346315	2149	pyoverdine sidechain synthetase III, L-Thr-L-Ser component

bioRxiv preprint doi: https://doi.org/10.1101/2025.02.13.638044; this version posted February 14, 2025. The copyright holder for this preprint (which was not certified by peer review) is the author/funder, who has granted bioRxiv a license to display the preprint in perpetuity. It is made available under aCC-BY 4.0 International license.

67	2445610	2222	sensor histidine kinase
366	2445618	2222	sensor histidine kinase
446	2445702	2222	sensor histidine kinase
322	2445745	2222	sensor histidine kinase
62	2445863	2222	sensor histidine kinase
534	2445871	2222	sensor histidine kinase
490	2445923	2222	sensor histidine kinase
345	2445940	2222	sensor histidine kinase
82	2445950	2222	sensor histidine kinase
70	2446019	2222	sensor histidine kinase
218	2446176	2222	sensor histidine kinase
792	2446222	2222	sensor histidine kinase
248	2446283	2222	sensor histidine kinase
764	2446368	2222	sensor histidine kinase
266	2489809	NA	106 bp intergenic region
61	3421882	NA	447 bp intergenic region
259	3468913	3087	hopAB2 ,type III effector HopAB2
789	3567970	3176	thermolysin metallopeptidase
309	3619914	NA	618 bp intergenic region
506	3899841	3457	short-chain fatty acid transporter
503	4147127	3680	methyl-accepting chemotaxis protein
234	4390369	3878	ABC transporter substrate-binding protein
527	4421429	3906	hypothetical protein
281	4503514	3993	kup ,potassium uptake protein
308	4643117	4119	estC ,carboxylesterase
539	4759280	4225	nadB ,L-aspartate oxidase
546	4759393	4225	nadB ,L-aspartate oxidase
274	4759468	4225	nadB ,L-aspartate oxidase
549	4759759	4225	nadB ,L-aspartate oxidase
22	4787767	NA	308 bp intergenic region
133	4809323	4270	ISPsy4, transposition helper protein
134	4809323	4270	ISPsy4, transposition helper protein
497	4860239	4309	catI ,3-oxoadipate:succinyl-CoA transferase subunit A

bioRxiv preprint doi: https://doi.org/10.1101/2025.02.13.638044; this version posted February 14, 2025. The copyright holder for this preprint (which was not certified by peer review) is the author/funder, who has granted bioRxiv a license to display the preprint in perpetuity. It is made available under aCC-BY 4.0 International license.

770	4891781	4340	insecticidal toxin protein
98	4962478	4397	mutT/nudix family protein
343	5610705	4950	orn ,oligoribonuclease
323	5770050	5066	ISPsy8, transposase OrfB
91	5815028	5114	hypothetical protein
176	5876742	NA	41 bp intergenic region
502	6014135	5288	ilvA-2 ,threonine dehydratase
321	6058555	5330	hypothetical protein
541	6081806	5348	hypothetical protein
683	6205735	5453	oxidoreductase, aldo/keto reductase family
495	6256388	5489	cytosolic long-chain acyl-CoA thioester hydrolase family protein
44	6256408	5489	cytosolic long-chain acyl-CoA thioester hydrolase family protein
154	6264157	5499	aspA ,aspartate ammonia-lyase
452	6390782	5610	gidA ,glucose-inhibited division protein A
426	6390809	5610	gidA ,glucose-inhibited division protein A
840	6393312	5611	trmE ,tRNA modification GTPase TrmE
749	6393435	5611	trmE ,tRNA modification GTPase TrmE

bioRxiv preprint doi: https://doi.org/10.1101/2025.02.13.638044; this version posted February 14, 2025. The copyright holder for this preprint (which was not certified by peer review) is the author/funder, who has granted bioRxiv a license to display the preprint in perpetuity. It is made available under aCC-BY 4.0 International license.

Table S2 Metabolome of WT/Δgsn infection

Peak	Time (min)	Peak (log2)	Change (log2fold)	P	P*	Annotation
9	8.51	-3.56	-0.225	0.580	0.737	NA
12	8.86	0.788	0.206	0.115	0.300	NA
18	9.96	-3.14	-0.264	0.314	0.563	NA
22	10.25	1.17	-0.0699	0.542	0.731	Tris(trimethylsilyl)hydroxylamine
23	11.07	0.173	0.0231	0.913	0.943	NA
24	11.22	0.663	0.724	0.0109	0.0606	NA
29	12.22	-2.20	0.0448	0.822	0.942	NA
30	12.34	-4.39	-0.227	0.474	0.688	NA
43	13.94	1.72	0.290	0.432	0.676	Silanol, trimethyl-, phosphate (3:1)
50	14.82	-0.565	0.241	0.272	0.517	Succinic acid (2TMS)
51	15.01	-2.97	-0.367	0.464	0.688	NA
53	15.12	0.0209	0.0312	0.866	0.943	Propanoic acid (2TMS)
57	15.54	-1.42	0.283	0.720	0.878	Fumaric acid (2TMS)
71	16.78	-1.12	-0.0990	0.833	0.943	Pentanedioic acid (TMS)
76	17.4	-2.14	-0.107	0.752	0.900	NA
81	18.05	3.17	0.482	0.208	0.423	Butanedioic acid (TMS); Malic acid (3TMS)
84	18.37	-1.15	0.589	0.092	0.255	Butane (4TMS)
87	18.87	-3.05	-0.357	0.0523	0.177	NA
88	19	-4.51	0.411	0.0679	0.207	NA
89	19.11	-2.61	0.0199	0.875	0.943	NA
90	19.29	-0.511	0.516	0.0181	0.0848	L-Threonic acid (TMS)
94	19.75	-5.793	-0.0532	0.887	0.943	NA
100	20.42	-2.14	0.135	0.502	0.712	NA
105	20.81	-3.75	0.556	0.0665	0.207	Xylose (4TMS) (1MEOX)
106	20.91	-4.72	0.555	0.241	0.475	NA
109	21.14	-2.80	0.158	0.142	0.320	Xylose (4TMS) (1MEOX)
112	21.42	-4.355	0.575	0.00833	0.0583	NA
113	21.53	-3.62	0.102	0.563	0.731	NA
115	21.73	-4.87	1.34	2.00E-05	**0.000609**	Rhamnose (4TMS) (1MEOX)
120	22.19	-3.62	-0.933	0.0104	0.0606	NA

bioRxiv preprint doi: https://doi.org/10.1101/2025.02.13.638044; this version posted February 14, 2025. The copyright holder for this preprint (which was not certified by peer review) is the author/funder, who has granted bioRxiv a license to display the preprint in perpetuity. It is made available under aCC-BY 4.0 International license.

123	22.38	-4.34	-0.657	0.374	0.634	NA
125	22.48	-3.74	-0.0142	0.962	0.962	NA
126	22.59	-0.359	-0.245	0.170	0.371	NA
127	22.71	-3.73	0.599	0.135	0.316	NA
130	23.01	0.551	-0.726	0.123	0.300	D-(-)-fructofuranose (5TMS)
131	23.12	-0.490	-0.0221	0.927	0.943	Shikimic acid (4TMS);<br>D-(-)-Fructofuranose (5TMS)
134	23.35	-3.63	-0.543	0.441	0.676	NA
137	23.67	2.10	-0.0246	0.927	0.943	Tetradecanoic acid (TMS)
139	23.98	2.34	-0.277	0.00860	0.0583	Fructose (5TMS) (1MEOX)
140	24.11	2.16	-0.225	0.0234	0.100	Fructose (5TMS) (1MEOX)
141	24.25	1.39	-0.933	0.00455	**0.0462**	D-(+)-Galactopyranose (5TMS)
142	24.33	2.57	-0.319	0.00804	0.0583	Glucose (5TMS) (1MEOX)
143	24.58	1.37	-0.420	0.00450	**0.0462**	Glucose (5TMS) (1MEOX)
145	24.86	-1.96	0.483	0.0347	0.125	NA
148	25.13	-4.10	-0.374	0.121	0.300	NA
150	25.45	1.460	-0.714	0.0322	0.123	β-D-allopyranose (5TMS);<br>β-D-glucopyranose (5TMS);<br>α-D-(+)-talopyranose (5TMS)
158	26.59	-3.57	-0.206	0.679	0.846	NA
159	26.86	0.0129	-0.0302	0.905	0.943	Inositol, myo (6TMS)
160	27.13	-0.711	0.989	0.00206	**0.0314**	Galactose (5TMS) (1MEOX)
162	27.32	-5.012	0.887	0.0246	0.100	NA
163	27.41	-3.26	0.836	0.0826	0.240	Galactose (5TMS) (1MEOX)
164	27.49	-2.64	1.245	0.0174	0.0848	Galactose (5TMS) (1MEOX)
168	27.83	-4.77	-0.216	0.531	0.731	NA
181	29.58	2.81	8.199	4.14E-09	**2.53E-07**	NA
196	31.88	-1.025	0.221	0.558	0.731	Sucrose (8TMS)
200	32.56	-2.14	0.428	0.299	0.552	Sucrose (8TMS)
203	32.9	-1.39	0.352	0.355	0.619	NA
204	33.08	2.87	0.179	0.411	0.661	Sucrose (8TMS)
207	33.63	-1.84	0.332	0.411	0.661	Sucrose (8TMS)
211	34.14	1.363	1.589	3.53E-05	**7.18E--04**	Trehalose α,α' (8TMS)
212	34.25	-1.64	0.410	0.178	0.375	NA

bioRxiv preprint doi: https://doi.org/10.1101/2025.02.13.638044; this version posted February 14, 2025. The copyright holder for this preprint (which was not certified by peer review) is the author/funder, who has granted bioRxiv a license to display the preprint in perpetuity. It is made available under aCC-BY 4.0 International license.

*, adjusted P value; NA, not annotated; TMS, Tetramethylsilyl

bioRxiv preprint doi: https://doi.org/10.1101/2025.02.13.638044; this version posted February 14, 2025. The copyright holder for this preprint (which was not certified by peer review) is the author/funder, who has granted bioRxiv a license to display the preprint in perpetuity. It is made available under aCC-BY 4.0 International license.

Table S3. Bacterial strains used in this study

ID	Name	Description	Res*	Reference
Pseudomonas syringae				
	WT	*P. syringae* pv. *tomato* DC3000 Δ*hopQ1-1* lacking the effector *hopQ1* allowing it to cause disease in the model plant *N. benthamiana*	Rif	(*3*)
PsNS6	WT *lacZ*	WT carrying pHRP308 plasmid expressing *lacZ* B-galactosidase	Rif, Gent	This work, (*70*)
PsNS40	Δ*gsn*	WT with a deletion of the *gsn* cluster (PSPTO_0834-0838)	Rif	This work
PsNS101	Δ*gsn* Empty vector	Δ*gsn* carrying pBBR1MCS2 Empty vector plasmid	Rif, Tet	This work
PsNS103	Δ*gsn* *gsn* cluster	Δ*gsn* carrying pBBR1MCS2:*gsn* plasmid for expressing the *gsn* cluster under native promoter	Rif, Tet	This work
bim322	*rhpS*-1	WT *lacZ* with a mini-Tn5 transposon insertion disrupting *rhpS* (PSPTO_2222)	Rif, Gent, Kan	This work
bim244	*hrpR*-1	WT *lacZ* with a mini-Tn5 transposon insertion disrupting *hrpR* (PSPTO_1379)	Rif, Gent, Kan	This work
bim442	*hrpR*-2	WT *lacZ* with a mini-Tn5 transposon insertion disrupting *hrpR* (PSPTO_1379)	Rif, Gent, Kan	This work
bim516	*hrpS*-1	WT *lacZ* with a mini-Tn5 transposon insertion disrupting *hrpS* (PSPTO_1380)	Rif, Gent, Kan	This work
bim436	*hrpS*-2	WT *lacZ* with a mini-Tn5 transposon insertion disrupting *hrpS* (PSPTO_1380)	Rif, Gent, Kan	This work
bim331	*hrpL*-1	WT *lacZ* with a mini-Tn5 transposon insertion disrupting *hrpL* (PSPTO_1404)	Rif, Gent, Kan	This work

bioRxiv preprint doi: https://doi.org/10.1101/2025.02.13.638044; this version posted February 14, 2025. The copyright holder for this preprint (which was not certified by peer review) is the author/funder, who has granted bioRxiv a license to display the preprint in perpetuity. It is made available under aCC-BY 4.0 International license.

bim511	*hrpL*-2	WT *lacZ* with a mini-Tn5 transposon insertion disrupting *hrpL* (PSPTO_1404)	Rif, Gent, Kan	This work
PsNS151	empty vector *lux*	WT with a genomic integration of Tn7 lux reporter Empty vector	Rif, Gent	This work
PsNS145	*gsnA:lux*	WT with a genomic integration of Tn7 lux reporter *gsnA:lux*	Rif, Gent	This work
PsNS146	*avrPtoB:lux*	WT with a genomic integration of Tn7 lux reporter *avrPtoB:lux*	Rif, Gent	This work
PsNS152	*oxb20:lux*	WT with a genomic integration of pRS-pOXB20:lux (constitutive)	Rif, Gent	This work
PsNS163	Δ*purF*	WT with a deletion of *purF* (PSPTO_3811), purine auxotroph	Rif	This work
PsNS166	Δ*purD*	WT with a deletion of *purD* (PSPTO_4867), purine auxotroph	Rif	This work
E. coli				
	pRK2013	Helper strain carrying pRK2013 plasmid for conjugal transfer	Kan	(*71*)
	Mini-Tn5 transposon	Donor strain carrying mini-Tn5-Km2 plasmid with promoterless GFP:lux	Kan	(*72*)
Agrobacterium tumefaciens				
	GV3101 pMP90	Disarmed *A. tumefaciens* carrying *vir* plasmid pMP90		

[*], Antibiotic resistance: Kanamycin (Kan); Tetracyline (Tet); Chloramphenicol (Chl); Gentamycin (Gent); and Carbenicillin (Carb).

bioRxiv preprint doi: https://doi.org/10.1101/2025.02.13.638044; this version posted February 14, 2025. The copyright holder for this preprint (which was not certified by peer review) is the author/funder, who has granted bioRxiv a license to display the preprint in perpetuity. It is made available under aCC-BY 4.0 International license.

Table S4. Plasmids used in this study

ID	Name	Description	Res*	Reference
pNS38	pK18mobsacB Δ*gsn*	used for generation of Δ*gsn* deletion mutant	*Kan*	This work
pNS172	pK18mobsacB Δ*purF*	used for generation of Δ*purF* deletion mutant	*Kan*	This work
pNS173	pK18mobsacB Δ*purD*	used for generation of Δ*purD* deletion mutant	*Kan*	This work
pNS35	pBBR1MCS2:*gsn*	plasmid with the *gsn* cluster under native promoter	*Tet*	This work
pNS99	pBBR1MCS3:*gsn*	plasmid with the *gsn* cluster under a constitutive Lac promoter	*Tet*	This work
pNS161	Tn7 lux reporter Empty vector	Tn7 transposon construct with no promoter:*luxCDABE*, derived from pRS-pOXB20:lux by removing pOXB20 promoter and inserting T0T1 terminator upstream to prevent unwanted transcription	*Gent*	This work
pNS163	Tn7 lux reporter *gsnA:lux*	Tn7 transposon construct with *gsnA* promoter:*luxCDABE* fusion for transcriptional activity reporter	*Gent*	This work
pNS164	Tn7 lux reporter *avrPtoB:lux*	Tn7 transposon construct with *avrPtoB* promoter:*luxCDABE* fusion for transcriptional activity reporter	*Gent*	This work
pNS141	pET-28b:LacZHis	pET-28b expressing LacZ with a C-terminal 6xHistidine tag, in T7 Express lysY/Iq expression strain (New England Biolabs)	*Kan*	This work
pNS177	pET-28b:PurF	pET-28b expressing PurF with a C-terminal Strep-tagII, in Lemo21(DE3) expression strain (New England Biolabs)	*Kan, Chl*	This work
pNS70	pET-28b:GsnA	pET-28b expressing GsnA with a C-terminal Strep-tagII, in T7 Express lysY/Iq expression strain (New England Biolabs)	*Kan*	This work

bioRxiv preprint doi: https://doi.org/10.1101/2025.02.13.638044; this version posted February 14, 2025. The copyright holder for this preprint (which was not certified by peer review) is the author/funder, who has granted bioRxiv a license to display the preprint in perpetuity. It is made available under aCC-BY 4.0 International license.

pNS155	pET-28b:GsnB	pET-28b expressing GsnB with a C-terminal Strep-tagII, in Lemo21(DE3) expression strain (New England Biolabs)	*Kan, Chl*	This work
pNS77	pET-28b:GsnC	pET-28b expressing GsnC with a C-terminal Strep-tagII, in T7 Express lysY/Iq expression strain (New England Biolabs)	*Kan*	This work
pNS153	pET-28b:sfGFP	pET-28b expressing sfGFP with a C-terminal Strep-tagII, in T7 Express lysY/Iq expression strain (New England Biolabs)	*Kan*	This work
pRK2013	pRK2013	Helper strain carrying pRK2013 plasmid for conjugal transfer	*Kan*	(*71*)
pK18mobsacB	pK18mobsacB Empty vector	pK18mobsacB plasmid used for generation of deletion mutants	*Kan*	(*42*)
pBBR1MCS2	pBBR1MCS2 Empty vector	pBBR1MCS2 plasmid used for gene expression	*Tet*	(*43*)
pBBR1MCS3	pBBR1MCS3 Empty vector	pBBR1MCS3 plasmid used for gene expression	*Tet*	(*43*)
pUXBF13	pUXBF13	pUXBF13 plasmid for Tn7 transposon integration	*Carb*	(*73*)
OXB20:lux	pRS-pOXB20:lux	Tn7 transposon construct with pOXB20:*luxCDABE* constitutive expression	*Gent*	(*44*)
pET-28b	pET-28b Empty vector	pET-28b plasmid for recombinant protein production under IPTG-inducible T7 promoter	*Kan*	Novagen
pBK26	35S::BGAL1	Binary vector for overexpression of BGAL1 by agroinfiltration	*Kan*	(2)

[*], Antibiotic resistance: Kanamycin (Kan); Tetracyline (Tet); Chloramphenicol (Chl); Gentamycin (Gent); and Carbenicillin (Carb).

bioRxiv preprint doi: https://doi.org/10.1101/2025.02.13.638044; this version posted February 14, 2025. The copyright holder for this preprint (which was not certified by peer review) is the author/funder, who has granted bioRxiv a license to display the preprint in perpetuity. It is made available under aCC-BY 4.0 International license.

Table S5. Primers used in this study

ID	Name	Sequence (5'-3')	Description
**Inverse PCR**			
oNS22	Tn5_R_R3	GGAACAGTAGACGAAATTATTGC TTCCATGAAGCTCTTCCAGTCTGA TGTCATGCCAT	inverse PCR amplification
oNS23	Tn5_R_F4	TTTATTCCTCCTTGTTAACCCGGG TACCGAGCTCGAATTCGCGCGGCC GCACTTGTGTATAAGAGTCAG	inverse PCR amplification
oNS15	Tn5_R_seq2	TTTATTCCTCCTTGTTAACCCG	sequencing inverse PCR products to determine transposon insertion site
***gsn* cluster expression constructs**			
oNS67	pBBR1MCS2_GA_seq_F2	CAAGGCGACAAGGTGCTGATG	colony PCR and sequencing for checking inserts in pBBR1MCS2
oNS48	pBBR1MCS3_GA_seq_F	GAACGGGTTGGCATGGATTGTAG	colony PCR and sequencing for checking inserts in pBBR1MCS3
oNS49	pBBR1MCS3_GA_seq_R	CTGCAAGGCGATTAAGTTGGGTA	colony PCR and sequencing for checking inserts in pBBR1MCS3 and 2
oNS68	0834_prom_F	TGCTTAATGAATTACAACAGTTTT TATGCACCGTTCCATTCAAACCCT TCGC	amplify the whole *gsn* cluster from native promoter
oNS206	0834_5'full_F	GATAAGCTTGATATCGAATTCCTG CAGCCCCCTGGTAGAAATGGAGT GATTATCAACTTATG	amplify the whole *gsn* cluster from *gsnA*
oNS63	0838_R2	CCGCTCTAGAACTAGTGGATCCCC CTCAAGGAGGAGGAATCACAGCC	amplify the whole *gsn* cluster until *gsnE*
**Deletion constructs**			
oNS34	pK18mobsacb_GA_F	GCTACTGGGCTATCTGGACAAGG GAA	amplify pK18mobsacB plasmid backbone

bioRxiv preprint doi: https://doi.org/10.1101/2025.02.13.638044; this version posted February 14, 2025. The copyright holder for this preprint (which was not certified by peer review) is the author/funder, who has granted bioRxiv a license to display the preprint in perpetuity. It is made available under aCC-BY 4.0 International license.

oNS35	pK18mobsacb_GA_R	CGGTATCAGCTCACTCAAAGGCGGTA	amplify pK18mobsacB plasmid backbone
oNS50	pK18mobsacb_GA_seq_F	TTGCTCACATGTTCTTTCCTGCG	colony PCR and sequencing for checking inserts in pK18mobsacB
oNS51	pK18mobsacb_GA_seq_R	TTCGCTTGCTGTCCATAAAACCG	colony PCR and sequencing for checking inserts in pK18mobsacB
oNS42	0834-8_del_L_F	TACCGCCTTTGAGTGAGCTGATACCGTAAGTACATCAGCCGCCACC	amplify left flanking region of *gsnA* for deletion
oNS43	0834-8_del_L_R	TCAAGGAGGAGGAATCACAGCCGCCGTCAGGAGCAAGCAT	amplify left flanking region of *gsnA* for deletion
oNS44	0834-8_del_R_F	ATGCTTGCTCCTGACGGCGGCTGTGATTCCTCCTCCTTGA	amplify right flanking region of *gsnE* for deletion
oNS45	0834-8_del_R_R	TTCCCTTGTCCAGATAGCCCAGTAGCGCTCACAACGGACAACTGGA	amplify right flanking region of *gsnE* for deletion
oNS407	purF_del_L_F	TACCGCCTTTGAGTGAGCTGATACCGGCTACAACGCCTATATCCGTACTG	amplify left flanking region of *purF* for deletion
oNS408	purF_del_L_R	GTTCAATTAGTTGTTGTACAGATCGATGATACCGACGATGCCACACATGC	amplify left flanking region of *purF* for deletion
oNS409	purF_del_R_F	GCATGTGTGGCATCGTCGGTATCATCGATCTGTACAACAACTAATTGAAC	amplify right flanking region of *purF* for deletion
oNS410	purF_del_R_R	TTCCCTTGTCCAGATAGCCCAGTAGCCTCTCGAGCCCTGCGTAGTG	amplify right flanking region of *purF* for deletion

bioRxiv preprint doi: https://doi.org/10.1101/2025.02.13.638044; this version posted February 14, 2025. The copyright holder for this preprint (which was not certified by peer review) is the author/funder, who has granted bioRxiv a license to display the preprint in perpetuity. It is made available under aCC-BY 4.0 International license.

oNS411	purD_del_L_F	TACCGCCTTTGAGTGAGCTGATAC CGCCTACGCCACTGACTCCGAG	amplify left flanking region of *purD* for deletion
oNS412	purD_del_L_R	GTCGTTTATTCCTGGTCTTCGCCG CCACTGCCAATGATCAAAACATTC AT	amplify left flanking region of *purD* for deletion
oNS413	purD_del_R_F	ATGAATGTTTTGATCATTGGCAGT GGCGGCGAAGACCAGGAATAAAC GAC	amplify right flanking region of *purD* for deletion
oNS414	purD_del_R_R	TTCCCTTGTCCAGATAGCCCAGTA GCCAATAGGTGTAGATCAGGCCC GC	amplify right flanking region of *purD* for deletion
**RT-PCR**			
oNS132	0834_qRT_F	ATCCCAACGAATACTGTGGCG	RT-PCR of *gsnA* (PSPTO_0834)
oNS133	0834_qRT_R	TGACACCTGCGATATCCAACC	RT-PCR of *gsnA* (PSPTO_0834)
oNS138	avrPtoB_qRT_F	CATACTCGTAACATGCTCCGC	RT-PCR of *avrPtoB* (PSPTO_3087)
oNS139	avrPtoB_qRT_R	TCGGGAAATGCTGGCGAAATA	RT-PCR of *avrPtoB* (PSPTO_3087)
oNS144	rpoD_qRT_F	TCCATCGCCAAGAAGTACACC	RT-PCR of *rpoD* (PSPTO_0537)
oNS145	rpoD_qRT_F	ATTCGAACTTGTCCACAGCCT	RT-PCR of *rpoD* (PSPTO_0537)
**Tn7 lux reporter constructs**			
oNS384	tn7_prom_seq_F	GGCATTTCACGGACTTTCATGG	colony PCR and sequencing for checking inserts in Tn7 lux reporter construct
oNS385	tn7_prom_seq_R	TGGCAGGTAAACACTATTATCACC A	colony PCR and sequencing for checking inserts in Tn7 lux reporter construct

bioRxiv preprint doi: https://doi.org/10.1101/2025.02.13.638044; this version posted February 14, 2025. The copyright holder for this preprint (which was not certified by peer review) is the author/funder, who has granted bioRxiv a license to display the preprint in perpetuity. It is made available under aCC-BY 4.0 International license.

oNS386	t0t1_tn7_prom_F	GCTTTTGAAGCTAATTCGATCATGCATGAGAGCTTGGACTCCTGTTGATAGA	amplify T0T1 terminator
oNS387	t0t1_tn7_prom_R	GCCGTTAATAATGAATGAAATTTTTTTAGTCATACGTATCCTCCAAGCCTGAGCTCCGGCGGATTTGTCCTACTCAG	amplify T0T1 terminator
oNS395	tn7_p0834_F	CTGAGTAGGACAAATCCGCCGGAGCCGTTCCATTCAAACCCTTCGC	amplify *gsnA* promoter
oNS396	tn7_p0834_R	GTCATACGTATCCTCCAAGCCTGAGAAGTTGATAATCACTCCATTTCTACCAGGA	amplify *gsnA* promoter
oNS397	tn7_pavrptoB_F	CTGAGTAGGACAAATCCGCCGGAGCGTGCTGCCCTCCGATACAC	amplify *avrPtoB* promoter
oNS398	tn7_pavrptoB_R	GTCATACGTATCCTCCAAGCCTGAGATGCTGATCCTCTCCGATCTTCTGC	amplify *avrPtoB* promoter
**Recombinant protein expression constructs**			
oNS207	pET28b_GA_F	GCTTGGATCCGGCTGCTAAC	amplify pET-28b plasmid backbone
oNS208	pET28b_GA_R	TGTATATCTCCTTCTTAAAGTTAAACAAAATTATTTCTAGAGGG	amplify pET-28b plasmid backbone
oNS209	pET28b_GA_seq_F	CGCGAAATGGAGTAATACGACT	colony PCR and sequencing for checking inserts in pET-28b
oNS210	pET28b_GA_seq_R	TCAAGACCCGTTTAGAGGCC	colony PCR and sequencing for checking inserts in pET-28b
oNS232	0834_pET28b_F	CCCTCTAGAAATAATTTTGTTTAACTTTAAGAAGGAGATATACAATGAAAGCACTGGGCTTAATGGATAAC	amplify *gsnA* (PSPTO_0834)
oNS233	0834_pET28b_R	GTTAGCAGCCGGATCCAAGCTTACTTTTCGAACTGCGGGTGGCTCCAG	amplify *gsnA* (PSPTO_0834)

bioRxiv preprint doi: https://doi.org/10.1101/2025.02.13.638044; this version posted February 14, 2025. The copyright holder for this preprint (which was not certified by peer review) is the author/funder, who has granted bioRxiv a license to display the preprint in perpetuity. It is made available under aCC-BY 4.0 International license.

		CTACCAAGATAAGCGAGATTTGG TGTGAATACG	
oNS234	0835_pET28b_F	CCCTCTAGAAATAATTTTGTTTAA CTTTAAGAAGGAGATATACAATG AAAGTTACTGTATTCAGTCAGATA TCAATTGATGG	amplify *gsnB* (PSPTO_0835)
oNS235	0835_pET28b_R	GTTAGCAGCCGGATCCAAGCTCA CTTTTCGAACTGCGGGTGGCTCCA GCTACCGTACGGGCTCCGATTGTT CGATT	amplify *gsnB* (PSPTO_0835)
oNS320	0836_pET28b_F	CCCTCTAGAAATAATTTTGTTTAA CTTTAAGAAGGAGATATACAATG CAGGCGATAGGGGCTGTTA	amplify *gsnC* (PSPTO_0836)
oNS322	0836_pET28b_R	GTTAGCAGCCGGATCCAAGCTTAC TTTTCGAACTGCGGGTGGCTCCAG CTACCTTTTTCTGCAAACCATTGC TTGATGTC	amplify *gsnC* (PSPTO_0836)
oNS417	pet28b_purF_F	CCCTCTAGAAATAATTTTGTTTAA CTTTAAGAAGGAGATATACAATG TGTGGCATCGTCGGT	amplify *purF* (PSPTO_3811)
oNS418	pet28b_purF_R	GTTAGCAGCCGGATCCAAGCTTAC TTTTCGAACTGCGGGTGGCTCCAG CTACCGTTGTTGTACAGATCGATG ATCGC	amplify *purF* (PSPTO_3811)
oNS368	GFP_F	CCCTCTAGAAATAATTTTGTTTAA CTTTAAGAAGGAGATATACAATG GCGCATCACCATCACCATC	amplify sfGFP
oNS369	GFP_R	GTTAGCAGCCGGATCCAAGCTCA CTTTTCGAACTGCGGGTGGCTCCA GCTACCTTTGTACAGTTCATCCAT ACCATGCG	amplify sfGFP

bioRxiv preprint doi: https://doi.org/10.1101/2025.02.13.638044; this version posted February 14, 2025. The copyright holder for this preprint (which was not certified by peer review) is the author/funder, who has granted bioRxiv a license to display the preprint in perpetuity. It is made available under aCC-BY 4.0 International license.

Table S6. Cryo-EM data collection, refinement and validation statistics

	Native WT (EMDB-19182) (PDB 8RI7)	*Δgsn* (EMDB-19181) (PDB 8RI6)	Synthetic galactosyrin (EMDB-19183) (PDB 8RI8)
**Data collection and processing**			
Magnification (nominal)	105k	105k	130k
Voltage (kV)	300	300	300
Detector	Gatan K3	Gatan K3	Falcon 4
Energy filter	BioQuantum 20 eV slit	BioQuantum 20 eV slit	Selectris X 10 eV slit
Electron exposure (e–/Å^2)	40	40	40
Defocus range (μm)	-0.5 to -2.1	-0.5 to -2.1	-0.5 to -2.1
Pixel size (Å)	0.829	0.831	0.921 with Super-resolution
Symmetry imposed	D2	D2	D2
Images (no.)	15,180	11,944	10,166
Final particle images (no.)	659,661	601,662	588,492
Map resolution (Å)	1.93	2.06	1.42
FSC threshold	0.143	0.143	0.143
**Refinement**			
Initial model used (PDB code)	De novo	De novo	De novo
Map sharpening *B* factor (Å^2)	-56.4061	-55.5941	-43.7367
Model composition			
Non-hydrogen atoms	33715	33662	33718
Protein residues	4064	4064	4064
Ligands	1	1	1
B factors (Å^2)			
Protein	5.44	14.73	13.79
R.m.s. deviations			
Bond lengths (Å)	0.006	0.003	0.008
Bond angles (°)	0.834	0.626	1.009

bioRxiv preprint doi: https://doi.org/10.1101/2025.02.13.638044; this version posted February 14, 2025. The copyright holder for this preprint (which was not certified by peer review) is the author/funder, who has granted bioRxiv a license to display the preprint in perpetuity. It is made available under aCC-BY 4.0 International license.

Validation			
MolProbity score	1.69	1.42	1.81
Clashscore	5.96	3.55	6.09
Poor rotamers (%)	1.29	1.03	1.90
Ramachandran plot			
Favored (%)	96.00	96.10	96.10
Allowed (%)	3.58	3.88	3.61
Disallowed (%)	0.42	0.02	0.30
